# Supplementary material for: Stabilization of Pin1 by USP34 promotes Ubc9 isomerization and protein sumoylation in glioma stem cells
Source: Nat Commun. 2024 Jan 2;15:40. doi: 10.1038/s41467-023-44349-x (PMC10762127; doi:10.1038/s41467-023-44349-x)

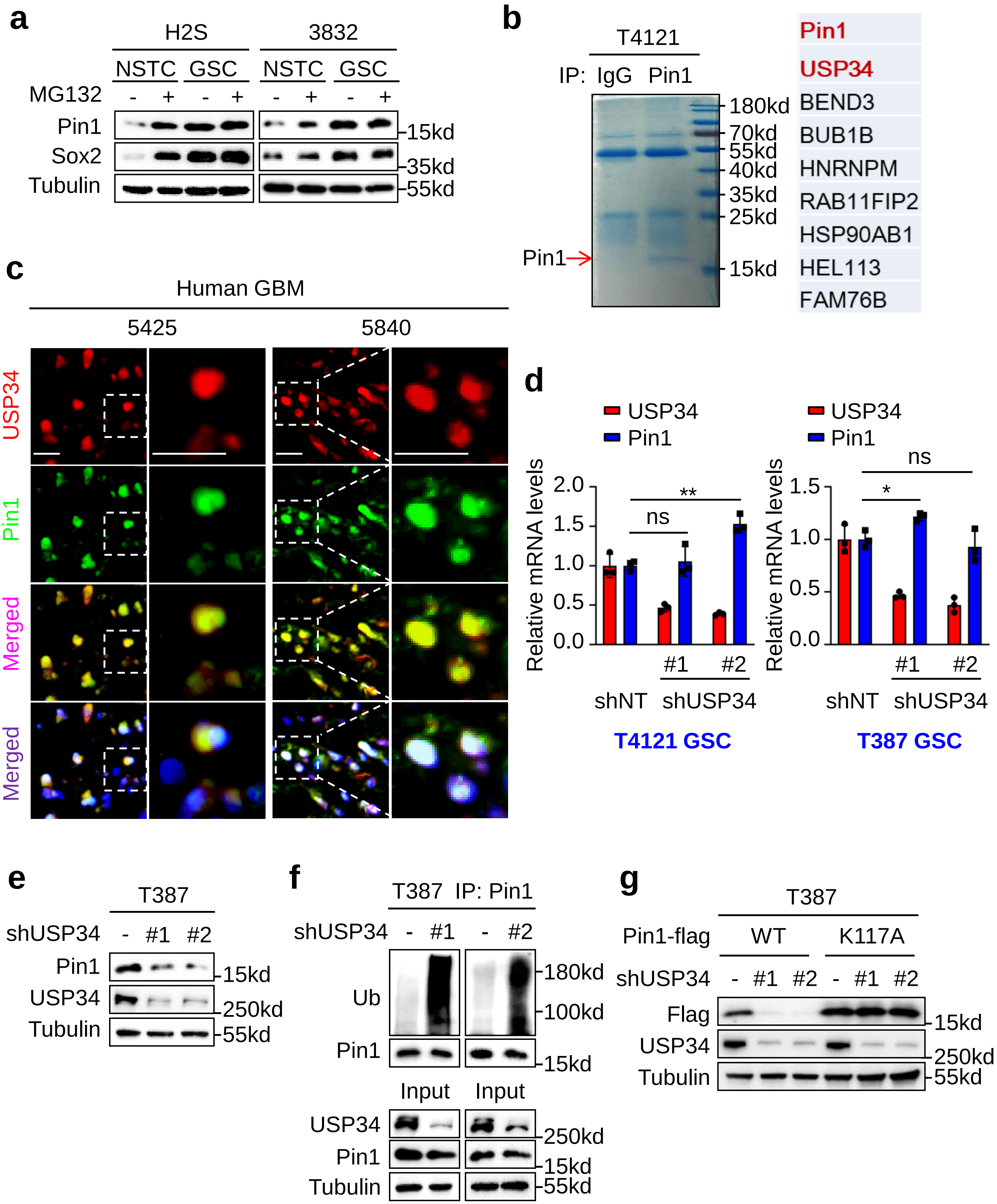

Supplementary Figure 1

Supplementary Fig. 1. Pin1 is deubiquitinated and stabilized by USP34 in GSCs

a Immunoblot analysis of Pin1 protein levels in GSCs and NSTCs treated with MG132 (10 μM) or DMSO for 6 hours.

b Coomassie blue staining (left) and mass spectrometry identification (right) of immunoprecipitation products containing the Pin1-interacting proteins. T4121 GSC lysate was subjected to immunoprecipitation with IgG or anti-Pin1 antibodies followed by gel electrophoresis and Coomassie blue staining. The heavy chain, light chain, and the band of Pin1 bait were cut off the gel, and the rest proteins were subjected to mass spectrometry analysis. The top 10 identified proteins were listed on the right.

c Representative images of immunofluorescent analysis of USP34 (red) and Pin1 (green) in human primary GBMs. Frozen sections of human GBMs were immunostained with antibodies against USP34 and Pin1, and counterstained with Hoechst to show nuclei (blue). Scale bar, 20 μm. The experiment was repeated for 5 times with similar results.

d Quantitative PCR analysis of Pin1 mRNA levels in GSCs after disruption of USP34. Data represent 3 biologically independent experiments. (T4121 GSC: shUSP34-2 vs. shNT, $P = 0.0022$; T387 GSC: shUSP34-1 vs. shNT, $P = 0.0139$; *, $P < 0.05$; **, $P < 0.01$; mean ± s.d.; two-tailed unpaired t-test).

e Immunoblot analysis of USP34 and Pin1 protein levels in T387 GSCs expressing shUSP34 or shNT.

f Immunoblot analysis of the polyubiquitination of endogenous Pin1 proteins in T387 GSCs expressing shUSP34. Cells were harvested 72 hours post-lentiviral infection. MG132 (10 μM) was added to cell culture 6 hours before harvest for the ubiquitination assay. Cell lysate was subjected to immunoprecipitation with anti-Pin1 antibodies followed by immunoblot with anti-Ub antibodies.

g Immunoblot analysis of the protein levels of the wild type Pin1 (WT) and the Pin1-K117A mutant in T387 GSCs expressing shUSP34. Ectopic flag-tagged Pin1 proteins were expressed in GSCs through lentiviral infection. Cells were further infected with shUSP34 lentiviruses and harvested 72 hours post infection.

The blotting experiments were repeated at least three times with biological replicates (**a**, **e-g**). Source data are provided as a Source Data file.

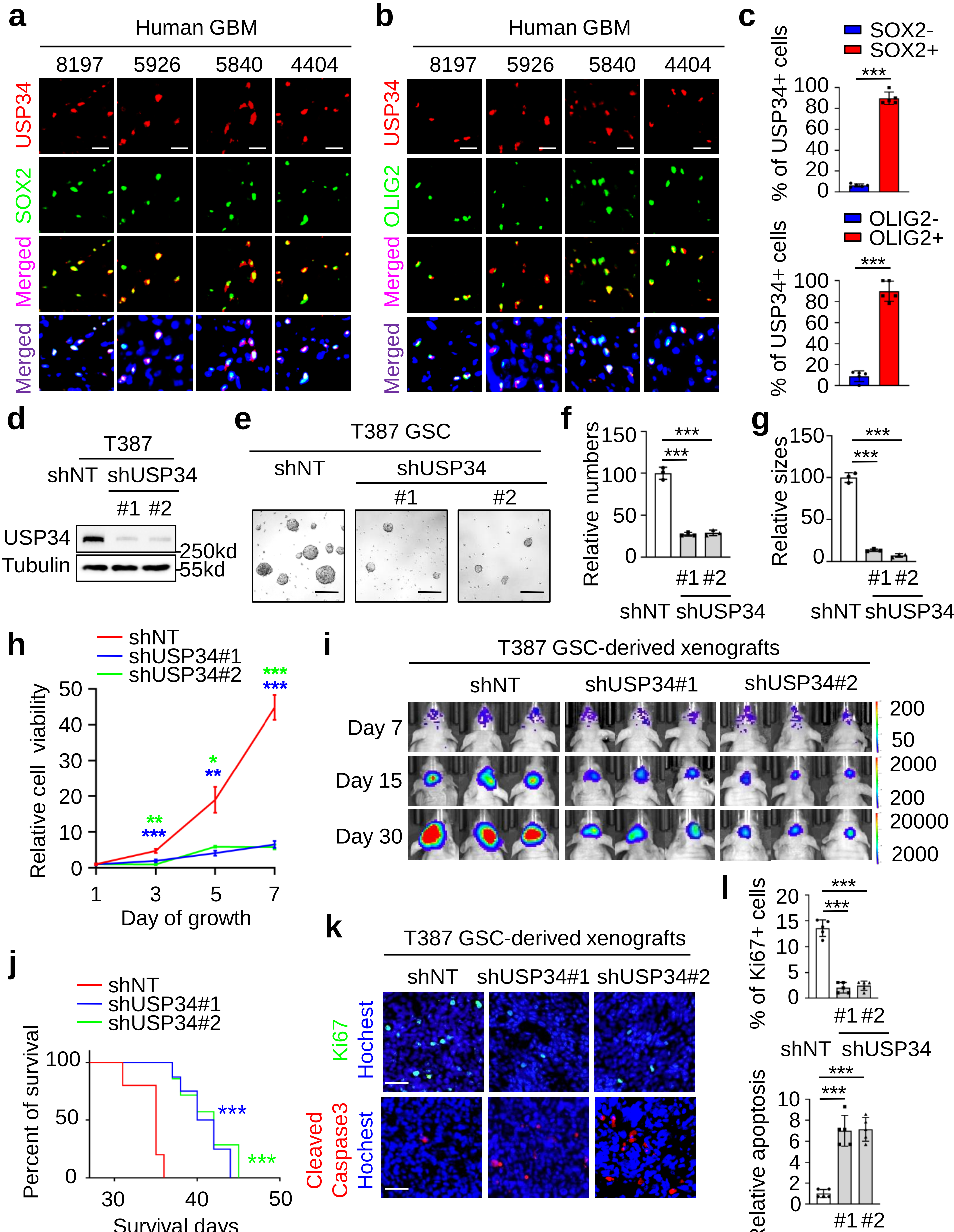

Supplementary Figure 2

Supplementary Fig. 2. USP34 is preferentially expressed in GSCs and promotes GSC maintenance and GBM tumor growth

a, b Representative immunofluorescent staining of USP34 (red) and SOX2 (**a**) or OLIG2 (**b**) (green) in human primary GBMs. Scale bar, 20 μm.

c Statistical quantification of immunofluorescent analysis from (**a** and **b**). (n = 5 biological replicates; ***, P < 0.001; mean ± s.d.; two-tailed unpaired t-test)

d Immunoblot of USP34 in T387 GSCs expressing shUSP34 or shNT.

e Representative images of tumorsphere formation of T387 GSCs expressing shUSP34 or shNT. Twenty-four hours after lentiviral infection, 2,000 cells were planted in each well of 96-well plates and cultured for 5 days. Scale bar, 50 μm.

f, g Statistical quantifications of tumorsphere numbers (**f**) and sizes (**g**) of T387 GSCs expressing shUSP34 or shNT. Three random 20× fields were used for calculation. (n = 3 biological replicates; ***, P < 0.001; mean ± s.d.; two tailed unpaired t-test).

h Cell proliferation of T387 GSCs expressing shUSP34 or shNT. Forty-eight hours post lentiviral infection, 2,000 cells were planted in each well of 96-well plates and cell viability was determined. (n = 4 biologically independent experiments; Day 3: shUSP34-2 vs. shNT, P = 0.0011; Day 5: shUSP34-1 vs. shNT, P = 0.0062; shUSP34-2 vs. shNT, P = 0.0108; *, P < 0.05; **, P < 0.01; ***, P < 0.001; mean ± s.d.; two-way ANOVA).

i *In vivo* bioluminescent imaging analysis of intracranial growth of GBM xenografts derived from T387 GSCs expressing shUSP34 or shNT.

j Kaplan-Meier survival curves of tumor-bearing mice in (**i**). (shNT, n = 5 mice; shUSP34#1, n = 8 mice; shUSP34#2, n = 7 mice; ***, P < 0.001; two-tailed log-rank test).

k, l Immunofluorescent analysis (**k**) and statistical quantifications (**l**) of Ki67 (green) and cleaved caspase-3 (red) in GBM xenografts. Scale bar, 40 μm (n = 5 tumors for each group; ***, P < 0.001; mean ± s.d.; two-tailed unpaired t-test).

The blotting experiments were repeated at least three times with biological replicates (**d**). Source data are provided as a Source Data file.

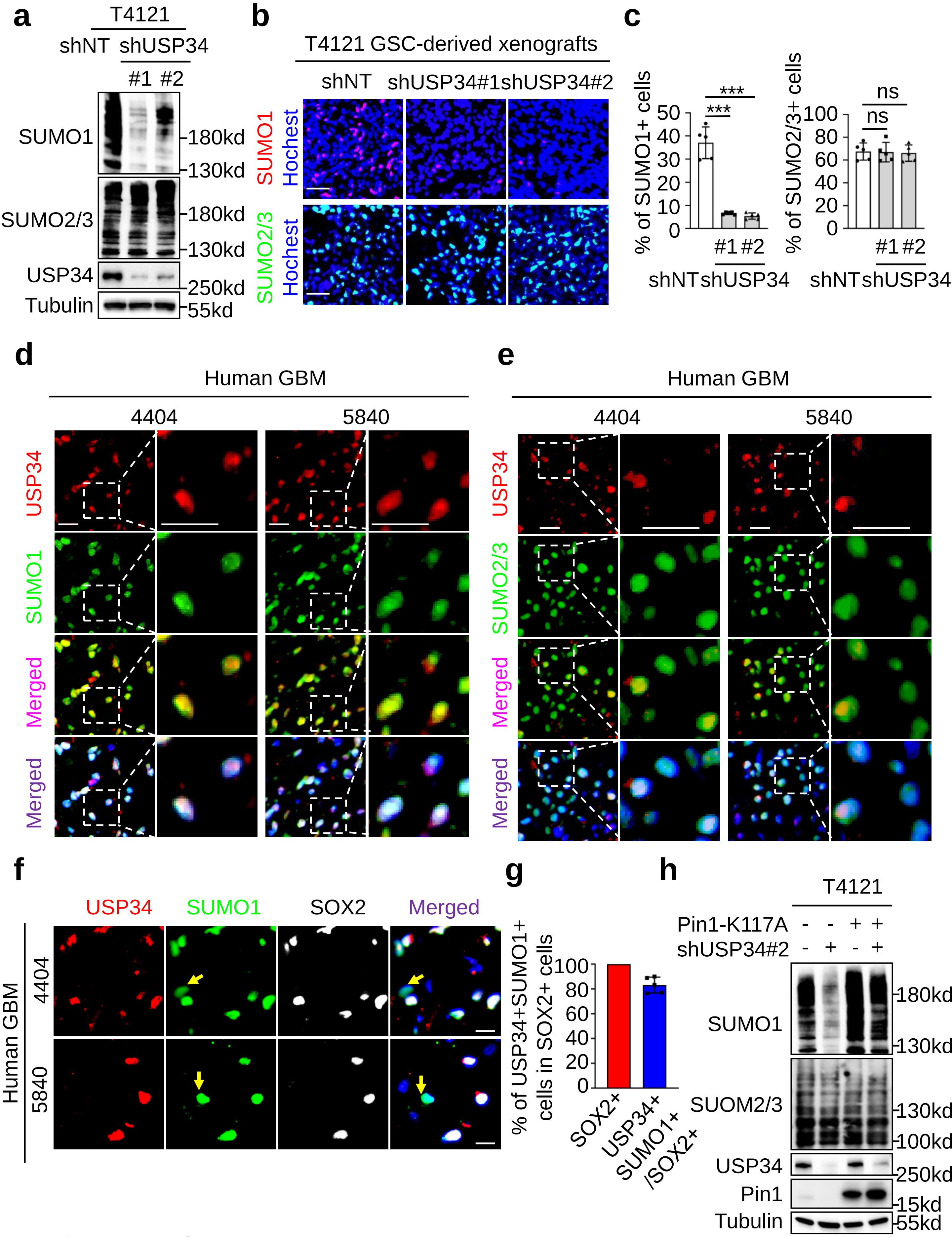

Supplementary Figure 3

Supplementary Fig. 3. USP34 promotes Pin1-mediated SUMO1-modified protein sumoylation in GSCs

a Immunoblot analysis of global protein sumoylation status in T4121 GSCs expressing shUSP34.

b, c Immunofluorescent analysis (**b**) and statistical quantifications (**c**) of protein sumoylation with SUMO1 (red) and SUMO2/3 (green) modifiers in xenografts derived from T4121 GSCs expressing shUSP34 or shNT. Scale bar, 40 µm (n = 5 tumors for each group; ***, $P < 0.001$; mean ± s.d.; two-tailed unpaired t-test).

d, e Representative images of immunofluorescent analyses of USP34 (red) and SUMO1 (**d**) or SUMO2/3 (**e**) (green) in human primary GBMs. Frozen sections of human GBMs were immunostained with antibodies against USP34 and SUMO1 or SUMO2/3, and counterstained with Hoechst to show nuclei (blue). Scale bar, 20 µm. (n = 5 biological replicates; mean ± s.d.)

f, g Representative images (**f**) and statistical quantification (**g**) of immunofluorescent analyses of USP34 (red), SUMO1 (green), and SOX2 (white) in human primary GBMs. The percentage of SOX2+ GSCs stained with both USP34 and SUMO1 was quantified. Very few SOX2- NSTCs that had SUMO1 staining showed no expression of USP34 (yellow arrows). Scale bar, 10 µm. (n = 5 biological replicates; mean ± s.d.)

h Immunoblot analysis of global protein sumoylation status in T4121 GSCs expressing shUSP34 and the ectopic Pin1-K117A mutant. GSCs were transduced with the ubiquitination-deficient Pin1-K117A mutant through lentiviral infection. The cells were further infected with shUSP34 lentivirus and the cell lysates were subjected to immunoblot analysis with indicated antibodies. The Pin1-K117A protein was stable even though the endogenous USP34 was reduced by shUSP34. Meanwhile, whereas disruption of USP34 reduced SUMO1- but not SUMO2/3-modified sumoylation, ectopic expression of the stable Pin1-K117 protein restored SUMO1-modified sumoylation in GSCs.

The blotting experiments were repeated at least three times with biological replicates (**a**, **h**).

Source data are provided as a Source Data file.

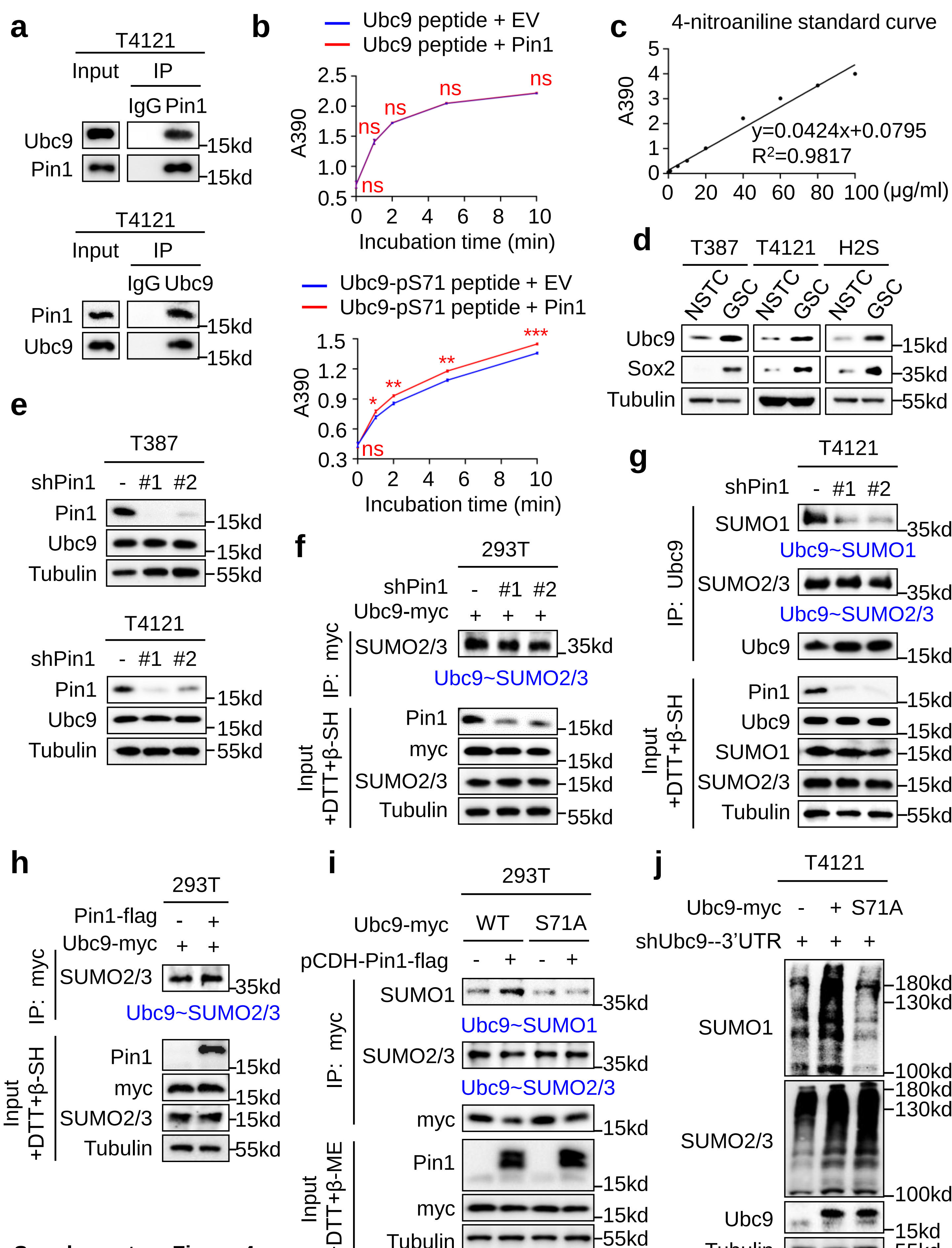

Supplementary Figure 4

Supplementary Fig. 4. Pin1 isomerizes Ubc9 to facilitate the formation of Ubc9-SUMO1 thioester

a Co-immunoprecipitation to determine the interaction between Pin1 and Ubc9 in T4121 GSCs.

b Cis-trans isomerization assay to determine the Pin1-catalyzed isomerization of the Ubc9 peptides with or without phosphorylation at S71. The Ubc9-pS71 and the control Ubc9 oligopeptides were incubated with control elution (EV) or flag-tagged Pin1 purified from 293T cells. The 4-nitroaniline released from the Ubc9 peptides was measured at 390 nm, and its change reflected the difference in isomerization of the prolyl bond. Data represent three independent experiments. (Lower panel: 1 min, $P = 0.0364$; 2 min, $P = 0.0074$; 5 min, $P = 0.0019$; *, $P < 0.05$; **, $P < 0.01$; ***, $P < 0.001$; means ± s.d.; two-way ANOVA)

c Standard curve of the absorbance at 390 nm (A390) of 4-nitroaniline for spectrophotometry, which was used for calculating 4-nitroaniline concentration for isomerization assays.

d Immunoblot analysis of Ubc9 in GSCs and NSTCs.

e Immunoblot analysis of Ubc9 and Pin1 in GSCs expressing shPin1 or shNT.

f Immunoblot analysis of the Ubc9-SUMO2/3 thioester (Ubc9~SUMO1) formation in 293T cells after disruption of Pin1. Immunoprecipitated Ubc9-SUMO2/3 conjugate was detected by anti-SUMO2/3 antibodies.

g Immunoblot analysis of the Ubc9-SUMO1 thioester (Ubc9~SUMO1) and Ubc9-SUMO2/3 thioester (Ubc9~SUMO2/3) formation in T4121 GSCs after disruption of Pin1.

h Immunoblot analysis of the Ubc9-SUMO2/3 thioester (Ubc9~SUMO1) formation in 293T cells overexpressing ectopic Pin1.

i Immunoblot analysis of the Ubc9-SUMO1 thioester (Ubc9~SUMO1) and Ubc9-SUMO2/3 thioester (Ubc9~SUMO2/3) formation in 293T cells overexpressing ectopic Pin1 along with wild type or the Ubc9-S71A mutant.

j Immunoblot analysis of global protein sumoylation status in T4121 GSCs expressing ectopic Ubc9. GSCs transduced with the wild type Ubc9 or the Ubc9-S71A mutant were further infected with lentiviruses carrying shUbc9-3'UTR. Cell lysates were subjected to immunoblot analysis with indicated antibodies.

The blotting experiments were repeated at least three times with biological replicates (**a**, **d-j**). Source data are provided as a Source Data file.

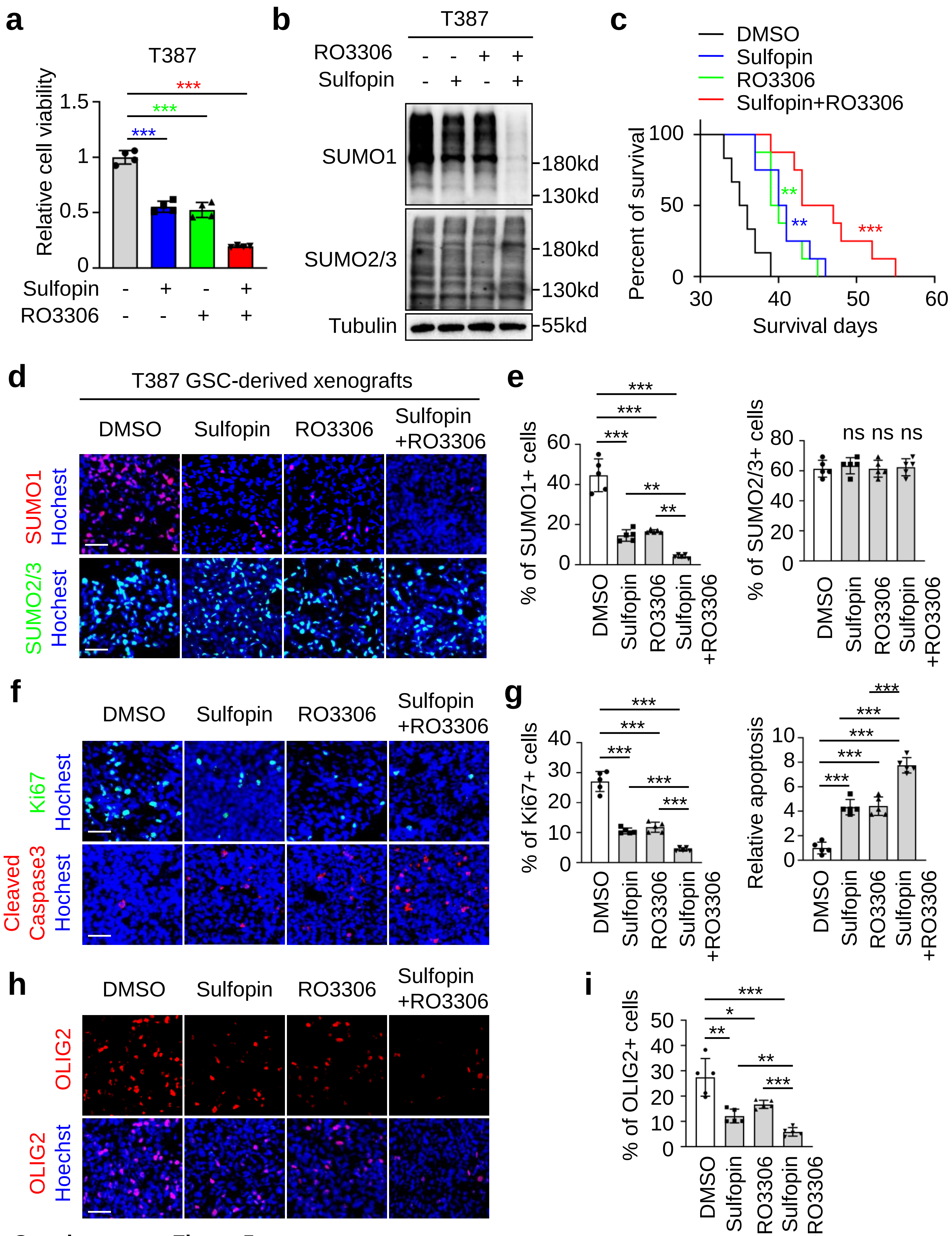

Supplementary Figure 5

Supplementary Fig. 5. Simultaneous inhibition of Pin1 and CDK1 suppresses SUMO1-modified sumoylation in GSCs and mitigates GBM growth

a Cell viability assays of T387 GSCs treated with the indicated drugs for 24 hours. GSCs were planted into 96-well plates at 10,000 cells per well. (n = 4 biological replicates; ***, $P < 0.001$; mean ± s.d.; one-way ANOVA).

b Immunoblot analysis of global protein sumoylation status in T387 GSCs treated with the indicated drugs for 24 hours.

c Kaplan-Meier survival curves of mice bearing GBMs derived from T387 GSCs with the indicated treatments. (DMSO, n = 6 mice; sulfopin, n = 8 mice; RO3306, n = 8 mice; sulfopin plus RO3306, n = 8 mice; sulfopin vs. DMSO, P = 0.0012; RO3306 vs. DMSO, P = 0.0021; **, $P < 0.01$; ***, $P < 0.001$; two-tailed log-rank test).

d, e Immunofluorescent analysis (**d**) and statistical quantifications (**e**) of the SUMO1-modified (red) and SUMO2/3-modified (green) protein sumoylation in T387 GSC-derived xenografts with the indicated treatments. Scale bar, 40 μm. (n = 5 tumors for each group; For % of SUMO1+ cells: sulfopin plus RO3306 vs. sulfopin, P = 0.0083; sulfopin plus RO3306 vs. RO3306, P = 0.0018; **, $P < 0.01$; ***, $P < 0.001$; mean ± s.d.; one-way ANOVA).

f, g Immunofluorescent analysis (**f**) and statistical quantifications (**g**) of Ki67 (green) and cleaved caspase-3 (red) in T387 GSC-derived xenografts with the indicated treatments. Scale bar, 40 μm. (n = 5 tumors for each group; ***, $P < 0.001$; mean ± s.d.; one-way ANOVA).

h, i Immunofluorescent analysis (**h**) and statistical quantifications (**i**) of OLIG2 in T387 GSC-derived xenografts with the indicated treatments. Scale bar, 40 μm. (n = 5 tumors for each group; sulfopin vs. DMSO, P = 0.0026; RO3306 vs. DMSO, P = 0.0138; sulfopin plus RO3306 vs. sulfopin, P = 0.0023; *, $P < 0.05$; **, $P < 0.01$; ***, $P < 0.001$; mean ± s.d.; two-tailed unpaired t-test).

The blotting experiments were repeated at least three times with biological replicates (**b**). Source data are provided as a Source Data file.

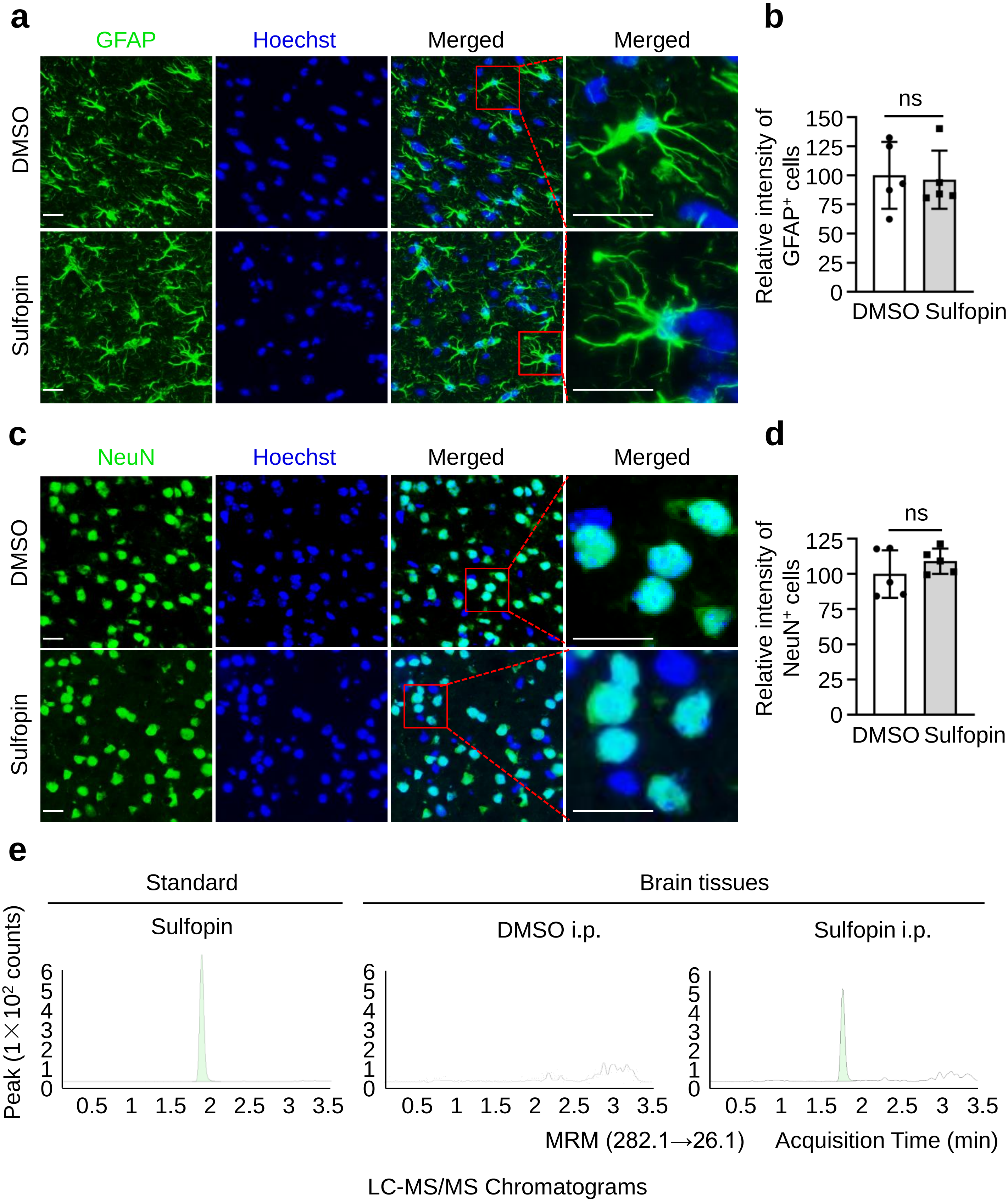

Supplementary Figure 6

Supplementary Fig. 6. Sulfopin is not toxic to normal brain cells

a, b Representative images (**a**) and statistical quantification (**b**) of the GFAP+ astrocytes in normal brain regions in mice bearing GSC-derived GBM tumors after treatment with DMSO or sulfopin. Sulfopin treatment did not affect the intensity or the morphology of normal astrocytes. Scale bar, 20 μm. (n = 5 brains for each group; mean ± s.d.; two-tailed unpaired t-test).

c, d Representative images (**c**) and statistical quantification (**d**) of the NeuN+ neurons in normal brain regions in mice bearing GSC-derived GBM tumors after treatment with DMSO or sulfopin. Sulfopin treatment did not affect the intensity or the morphology of normal neurons. (n = 5 brains for each group; mean ± s.d.; two-tailed unpaired t-test).

e Representative LS-MS/MS chromatograms of the detection of sulfopin in mouse brains. Mouse brain tissue was ground and mixed with sulfopin to generate Standard (200 ng/mL) for LC-MS/MS analysis (left). For analysis of the delivery of sulfopin into brains, mice were treated with DMSO or sulfopin (20 mg/kg) through intraperitoneal injection and mouse brains were harvested 5 minutes post treatment. The brains were then ground and subjected to LS-MS/MS analysis. No sulfopin was detected in the brains from mice treated with DMSO (middle). Meanwhile, clear sulfopin signals were detected in the brains from mice treated with sulfopin (right). The experiment was repeated for 5 times with similar results.

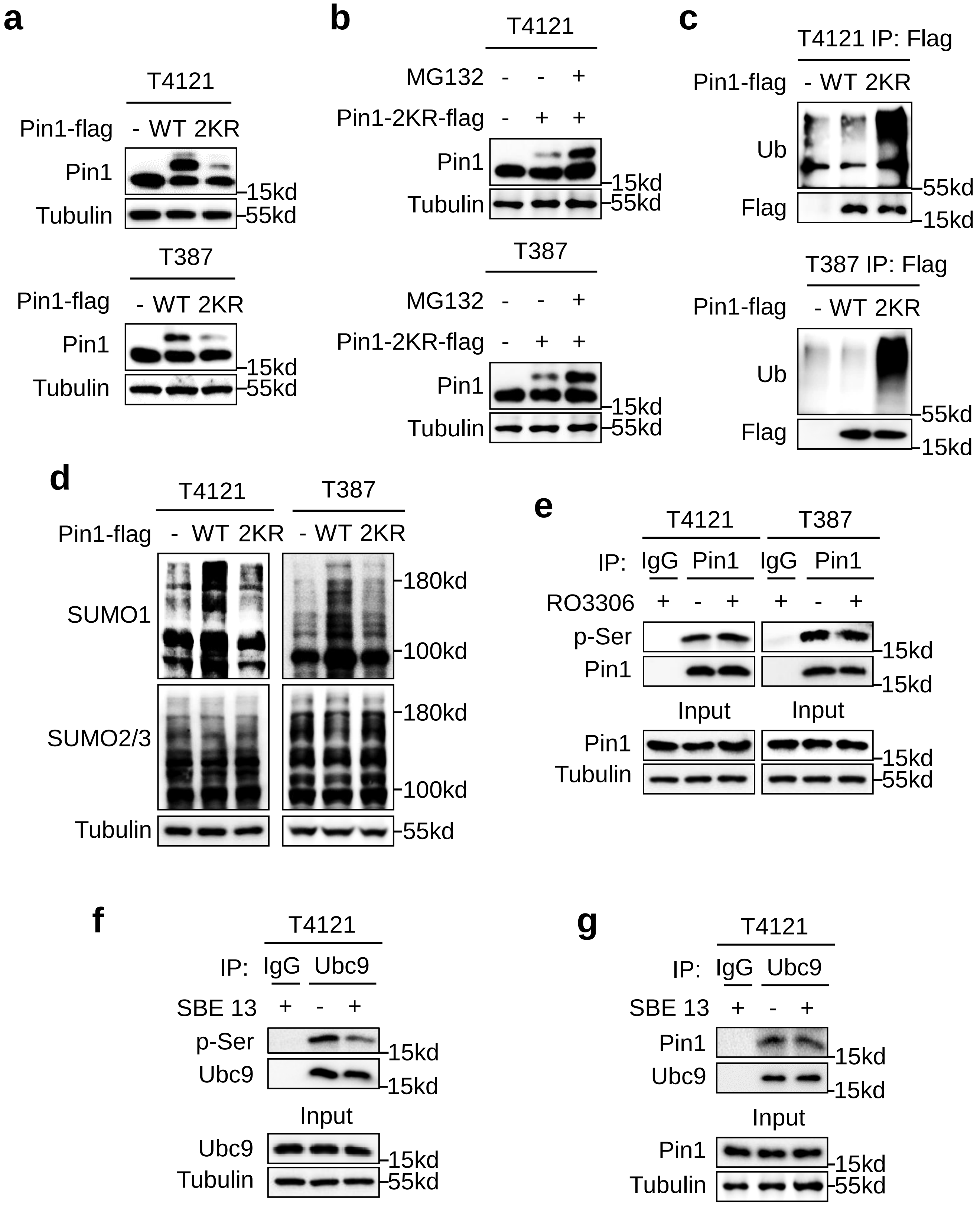

Supplementary Figure 7

Supplementary Fig. 7. Potential modifications on Pin1 and Ubc9

a Immunoblot analysis of the protein levels of the wildtype Pin1 (Pin1-WT) and the sumoylation deficient Pin1 mutant (Pin1-2KR) in which the K6 and K63 residues were substituted with arginine. Pin1-WT or Pin1-2KR was introduced into GSCs through lentiviral infection and cells were lysed 72 hours post infection.

b Immunoblot analysis of Pin1-2KR protein levels in GSCs treated with MG132 (10 μM) or DMSO for 6 hours.

c Immunoblot analysis of the poly-ubiquitination of the ectopic Pin1-WT and Pin1-2KR proteins in GSCs. Pin1-WT or Pin1-2KR was introduced into GSCs through lentiviral infection and cells were lysed 72 hours post infection. GSCs were treated with MG132 (10 μM) for 6 hours before harvest for ubiquitination assays. Cell lysate was subjected to immunoprecipitation with anti-Flag antibodies followed by immunoblot with anti-Ub antibodies.

d Immunoblot analysis of global protein sumoylation status in T4121 and T387 GSCs expressing ectopic Pin1-WT or Pin1-2KR proteins.

e Immunoblot analysis of the serine phosphorylation (pSer) status of Pin1 in GSCs treated with RO3306 (20 μM) or DMSO for 12 hours. Cell lysates were subjected to immunoprecipitation with IgG or anti-Pin1 antibodies followed by immunoblot with anti-pan-pSer antibodies.

f Immunoblot analysis of the serine phosphorylation (pSer) status of Ubc9 in GSCs treated with SBE 13 HCl (20 μM) or DMSO for 12 hours. Cell lysates were subjected to immunoprecipitation with IgG or anti-Ubc9 antibodies followed by immunoblot with anti-pan-pSer antibodies.

g Co-Immunoprecipitation to determine the interaction between Ubc9 and Pin1 in GSCs treated with SBE 13 HCl (20 μM) or DMSO for 9 hours. MG132 (10 μM) was added into cell culture 6 hours before harvest to obtain enough Pin1 proteins after Plk1 inhibition. Cell lysates were subjected to immunoprecipitation with IgG or anti-Ubc9 antibodies followed by immunoblot with anti-Pin1 antibodies.

The blotting experiments were repeated at least three times with biological replicates (**a-g**). Source data are provided as a Source Data file.

Source Data - Supplementary Figure 1

a

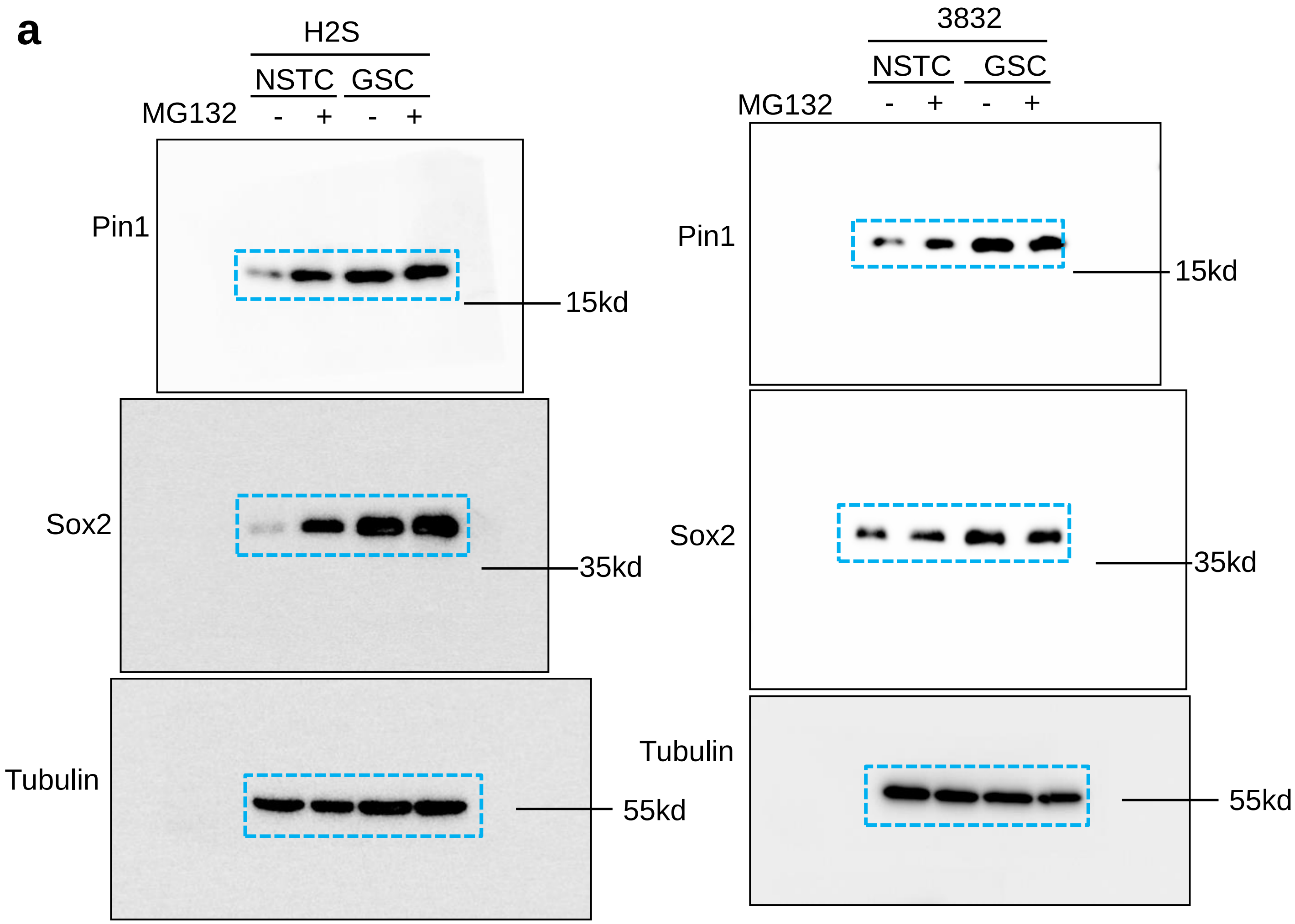

Source Data - Supplementary Figure 1

e

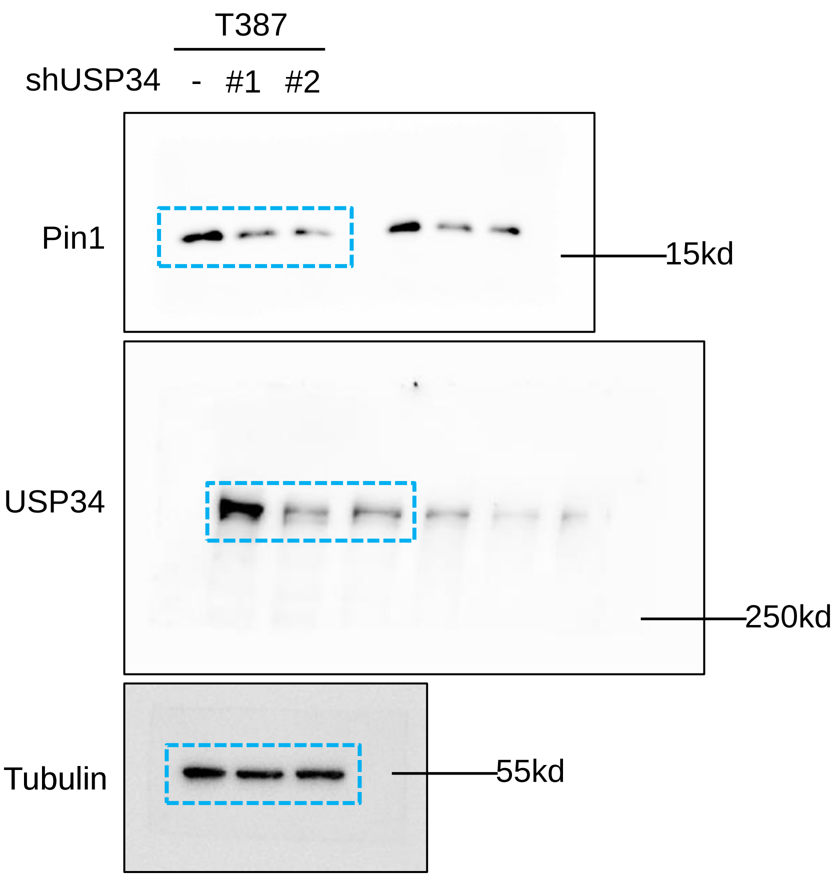

Source Data - Supplementary Figure 1

f

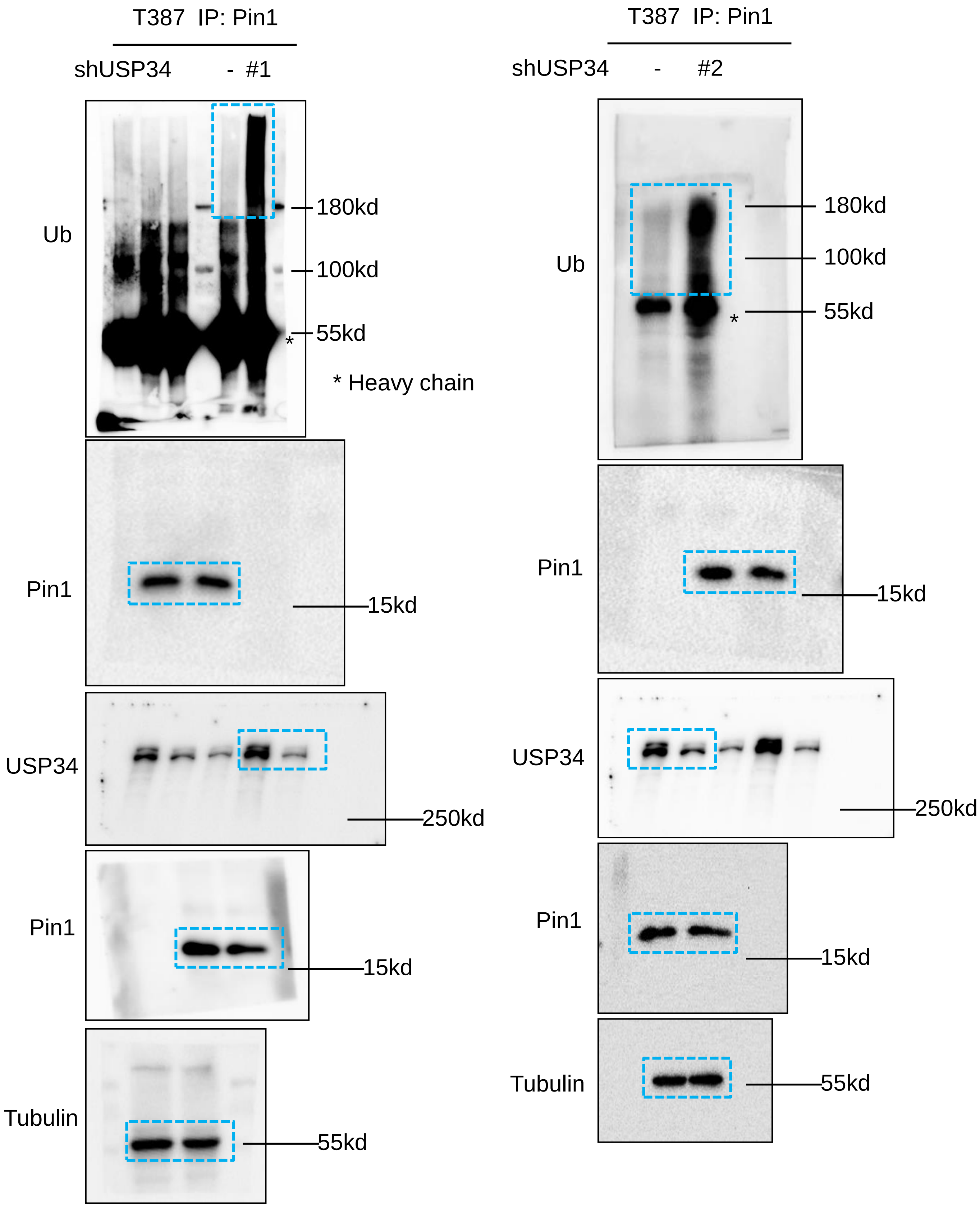

Source Data - Supplementary Figure 1

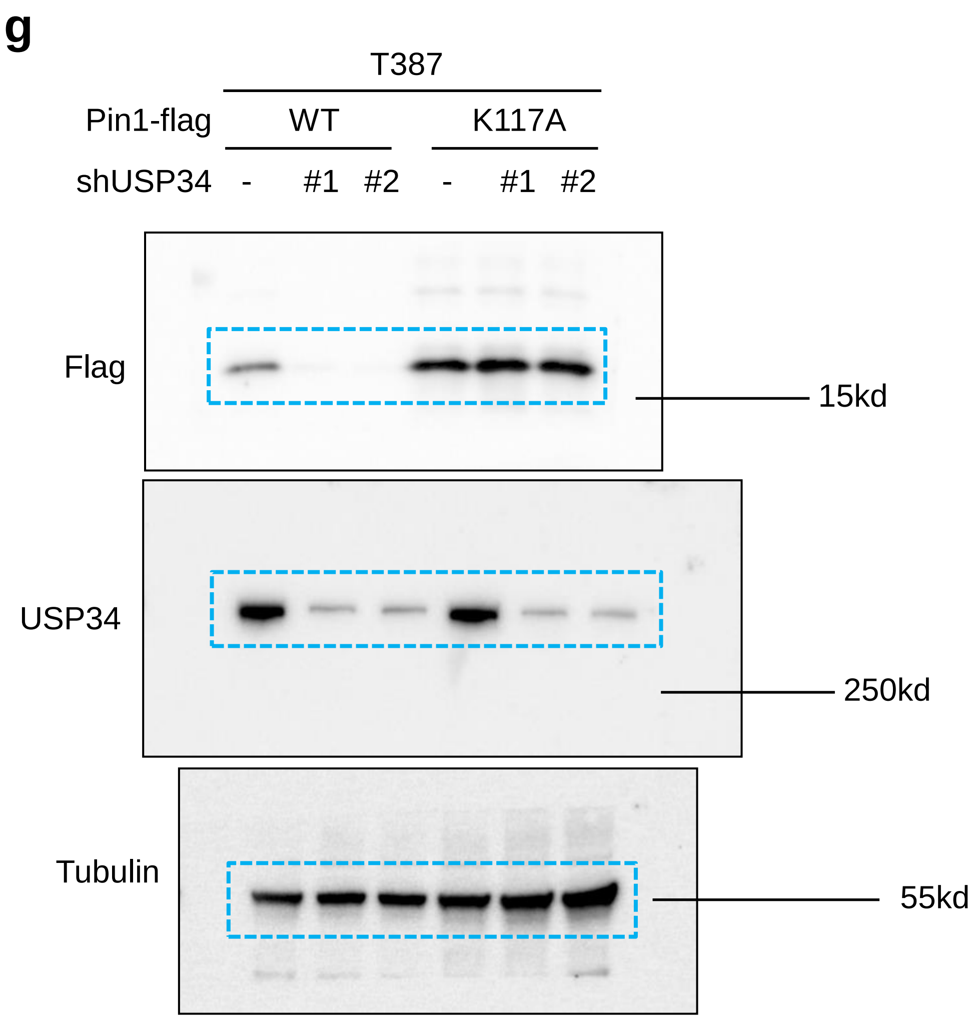

Source Data - Supplementary Figure 2

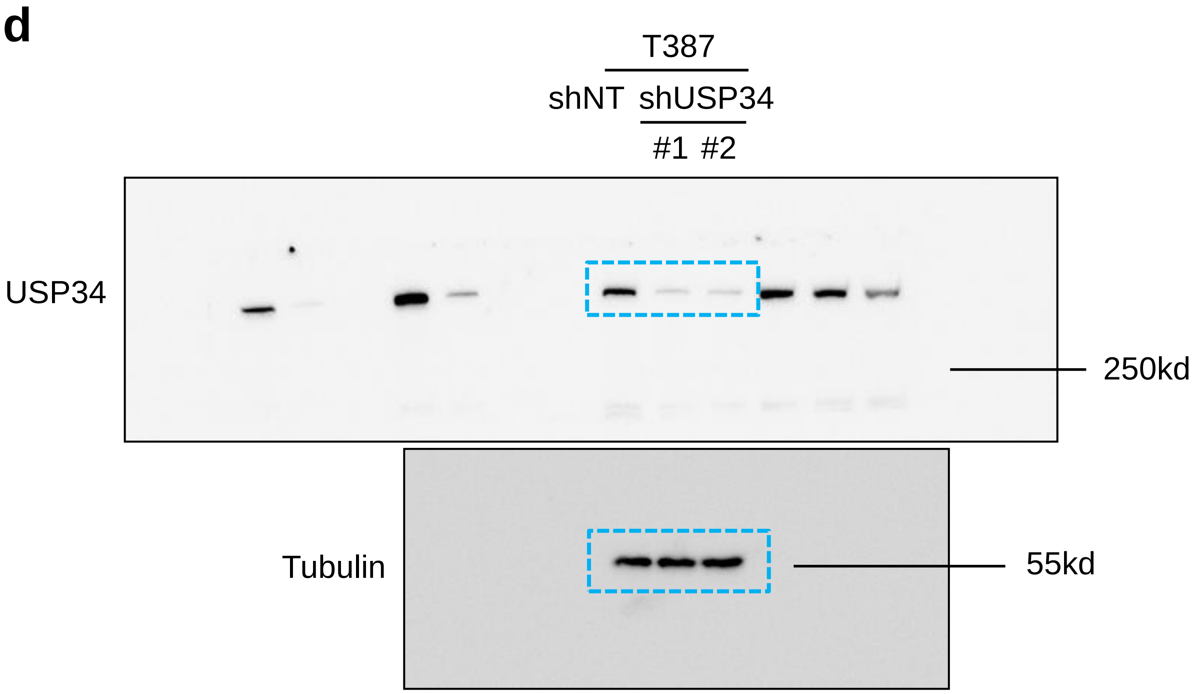

Source Data - Supplementary Figure 3

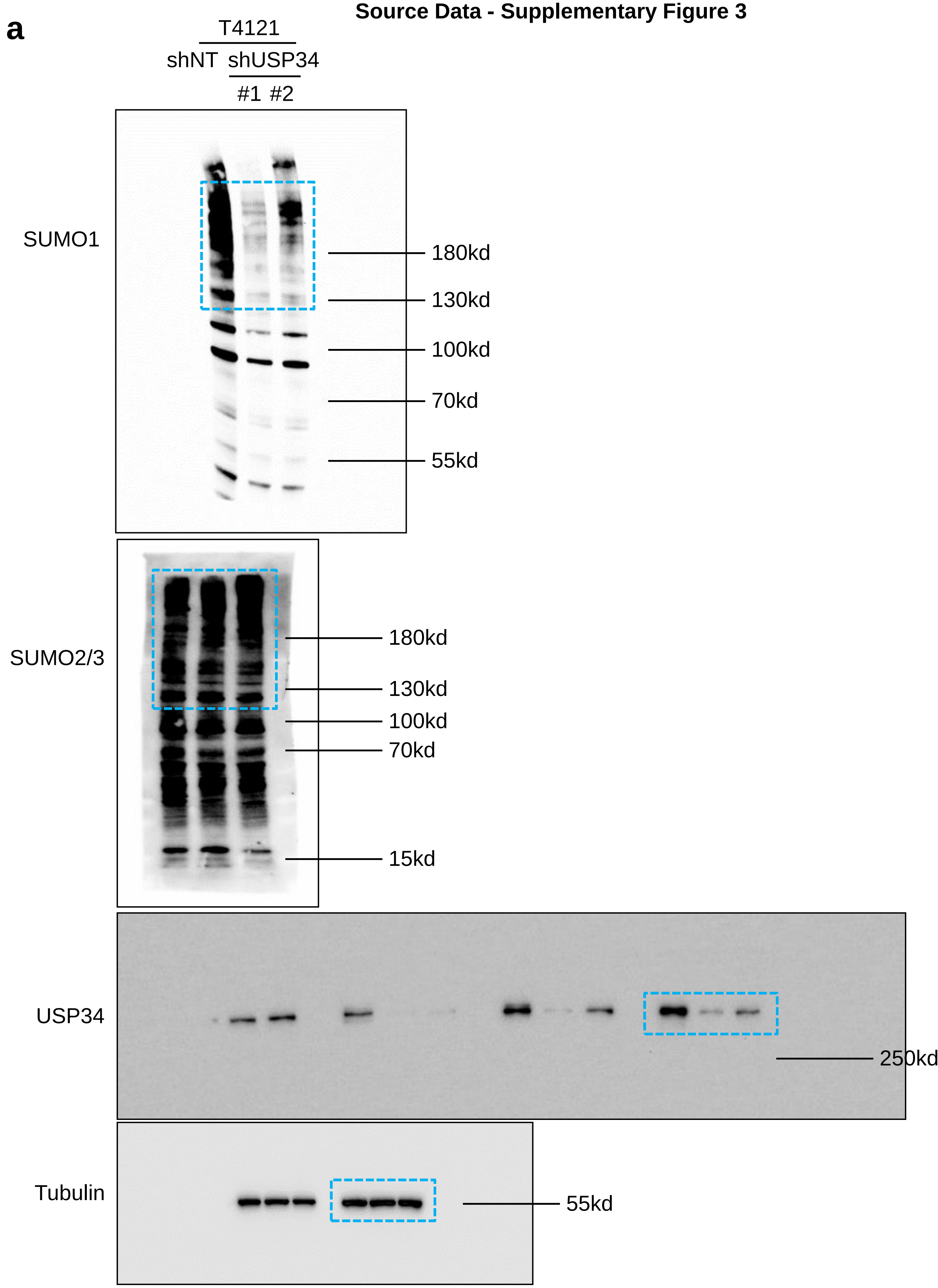

Source Data - Supplementary Figure 3

h

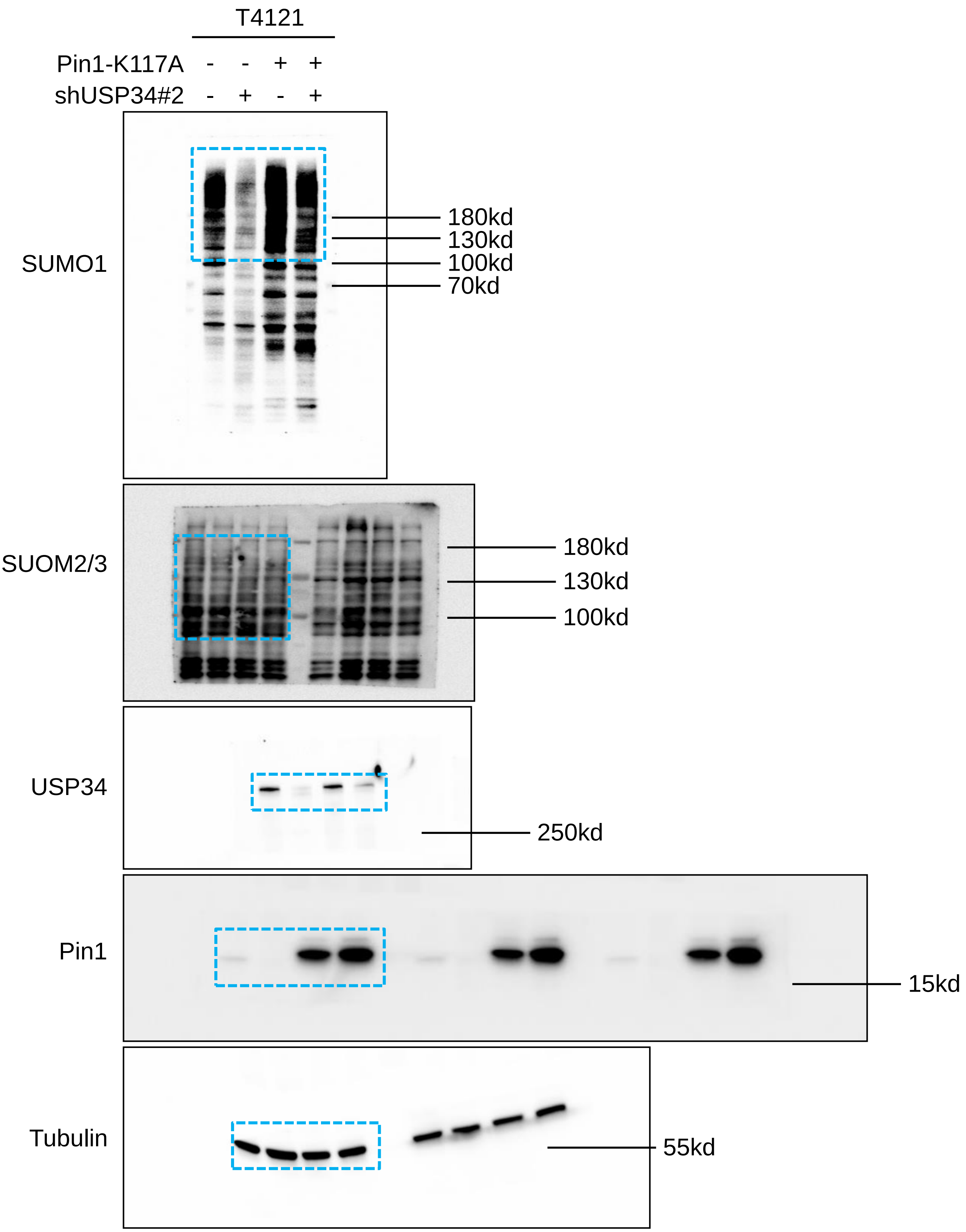

Source Data - Supplementary Figure 4

a

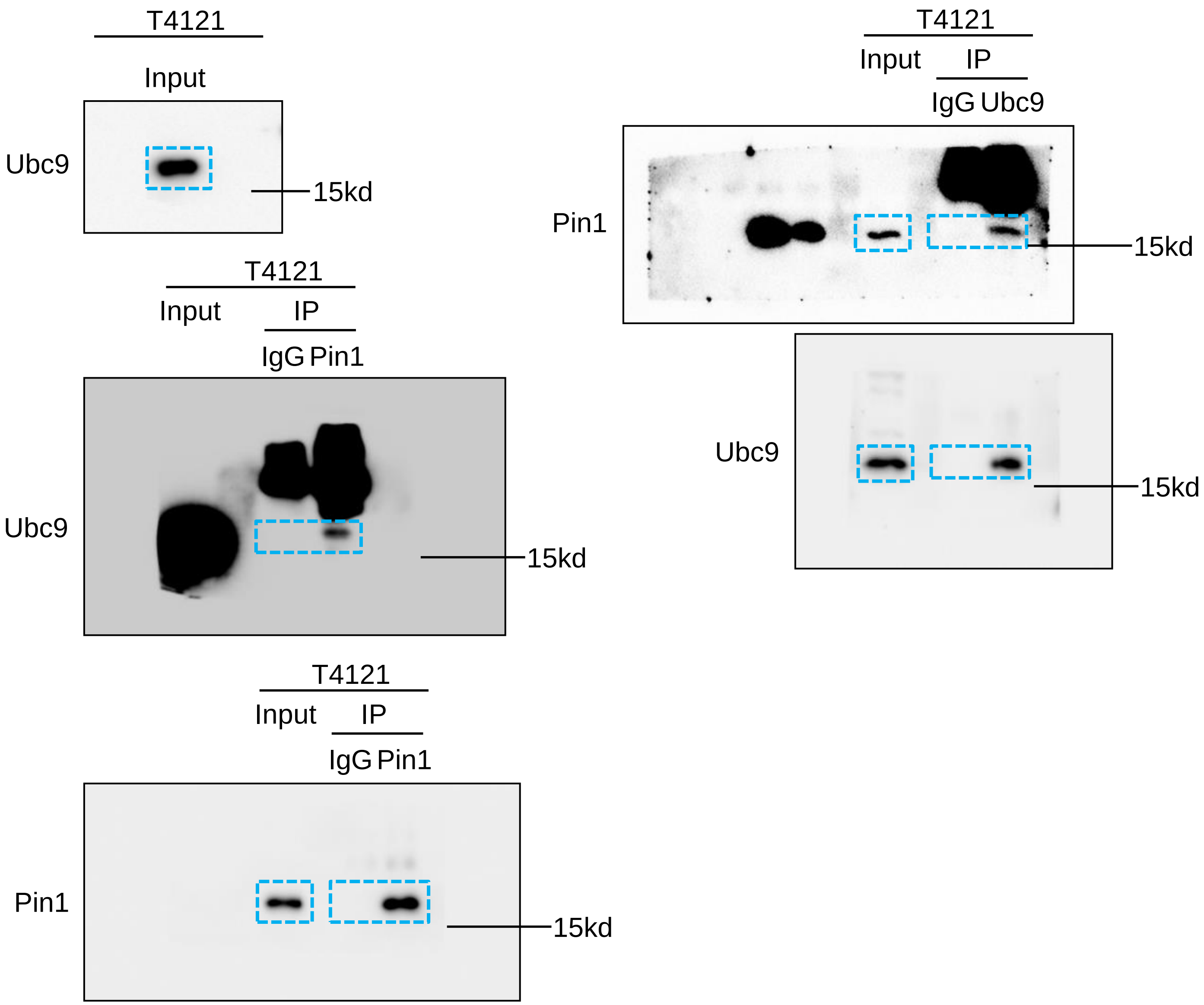

Source Data - Supplementary Figure 4

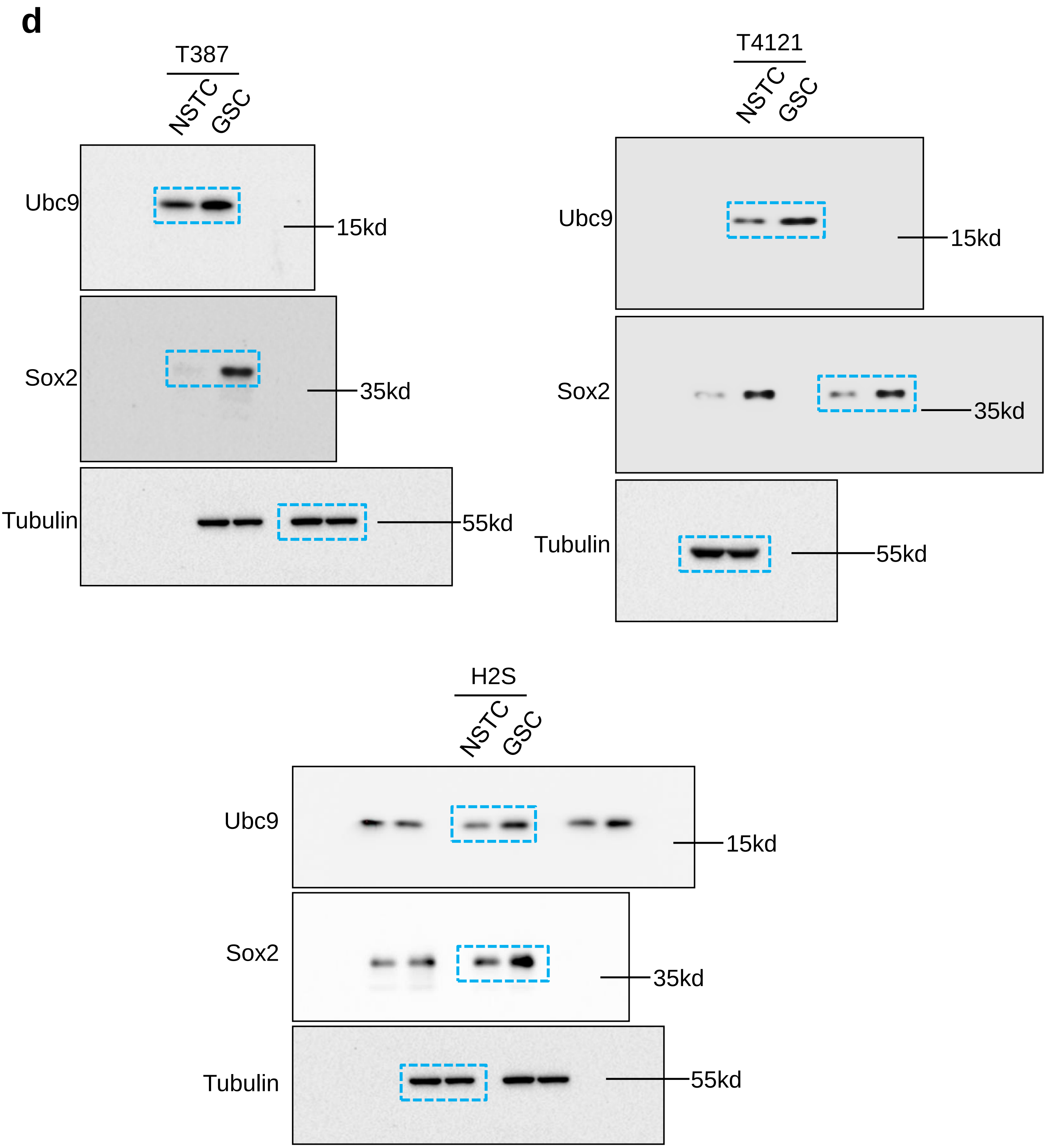

Source Data - Supplementary Figure 4

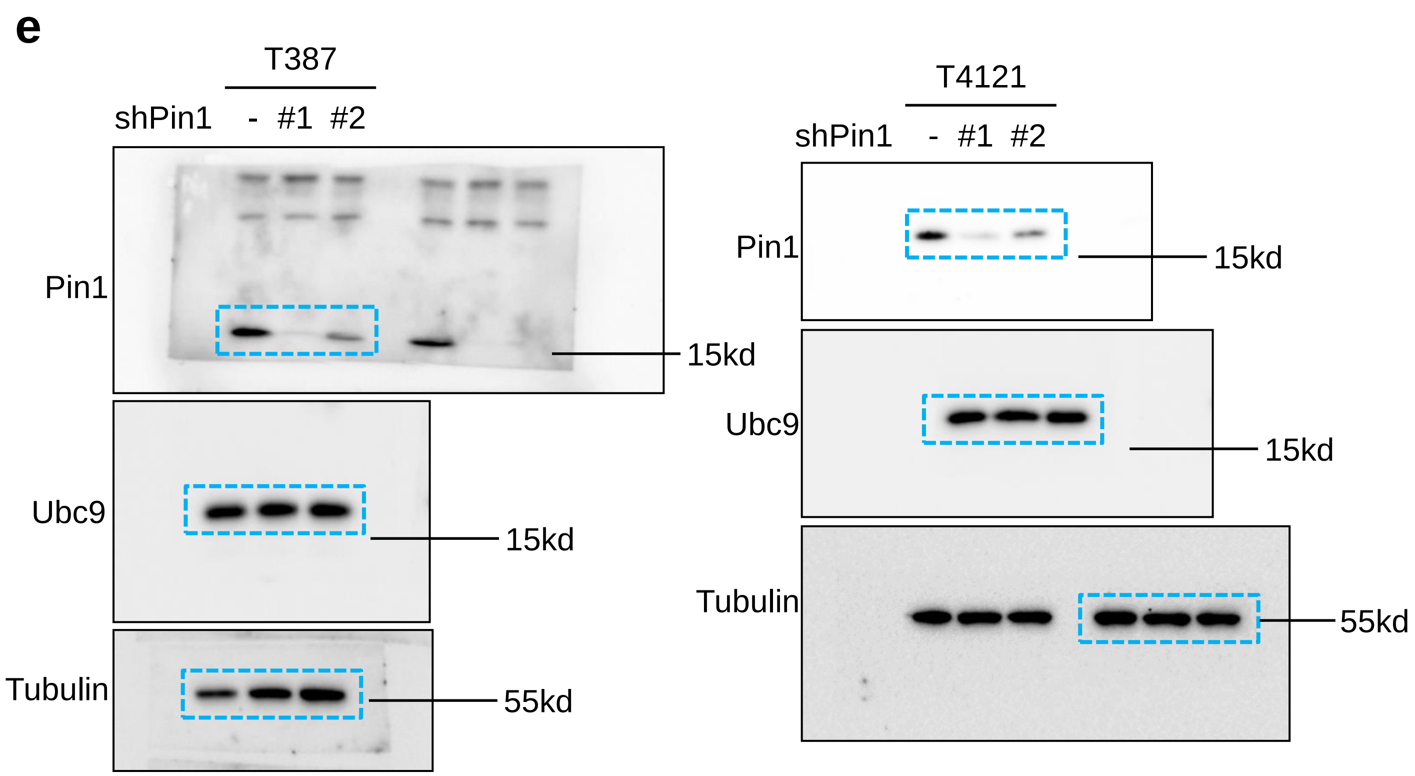

Source Data - Supplementary Figure 4

f

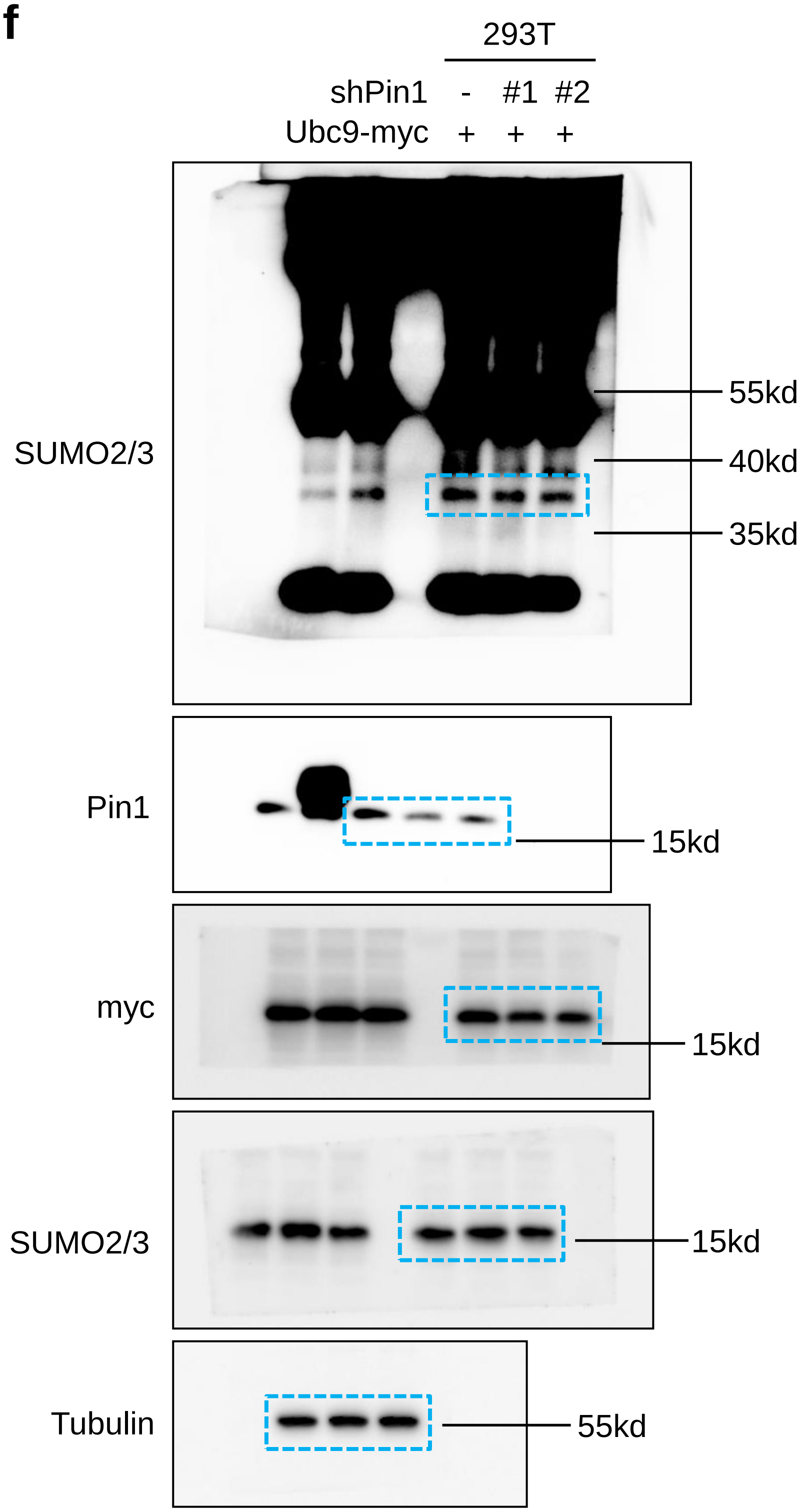

Source Data - Supplementary Figure 4

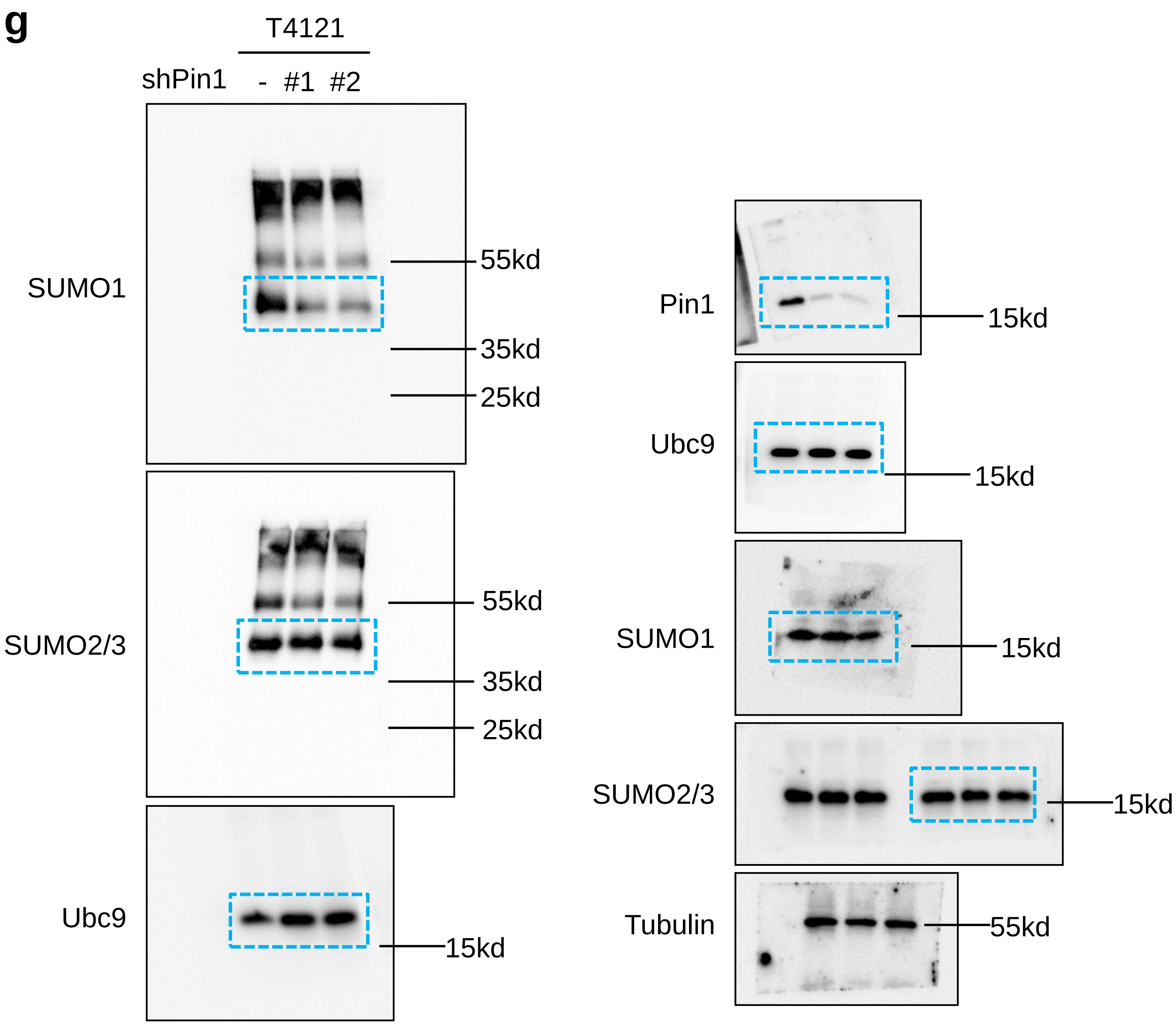

Source Data - Supplementary Figure 4

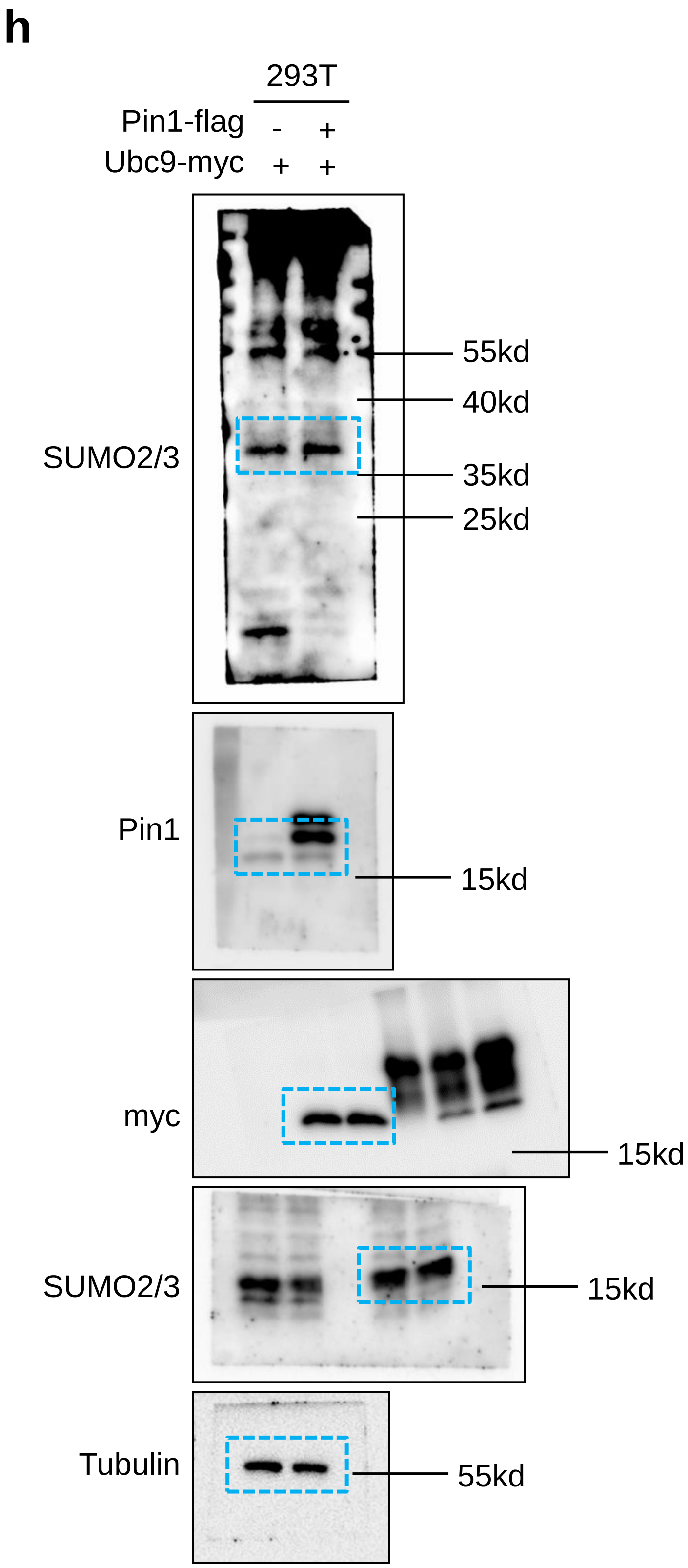

Source Data - Supplementary Figure 4

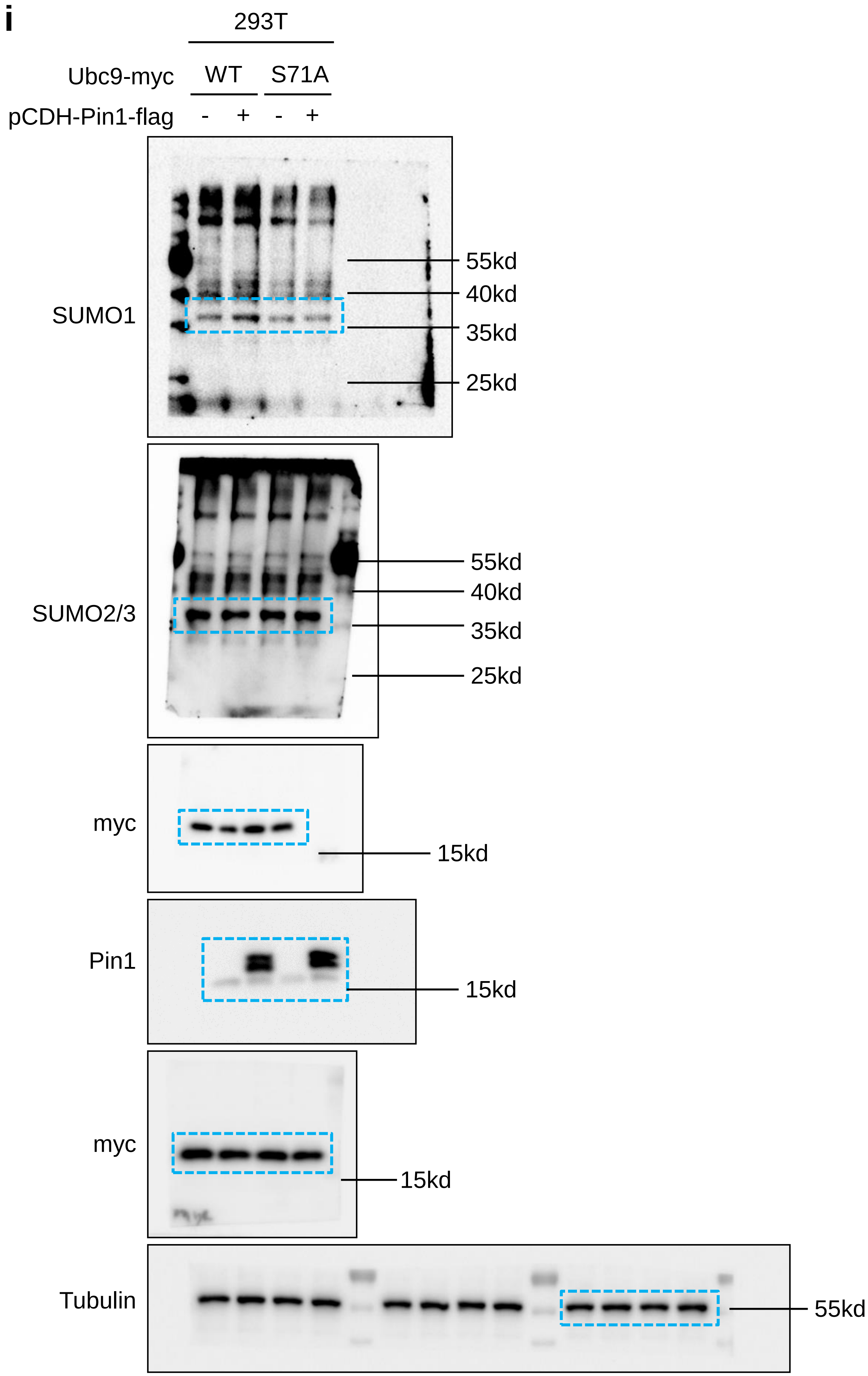

Source Data - Supplementary Figure 4

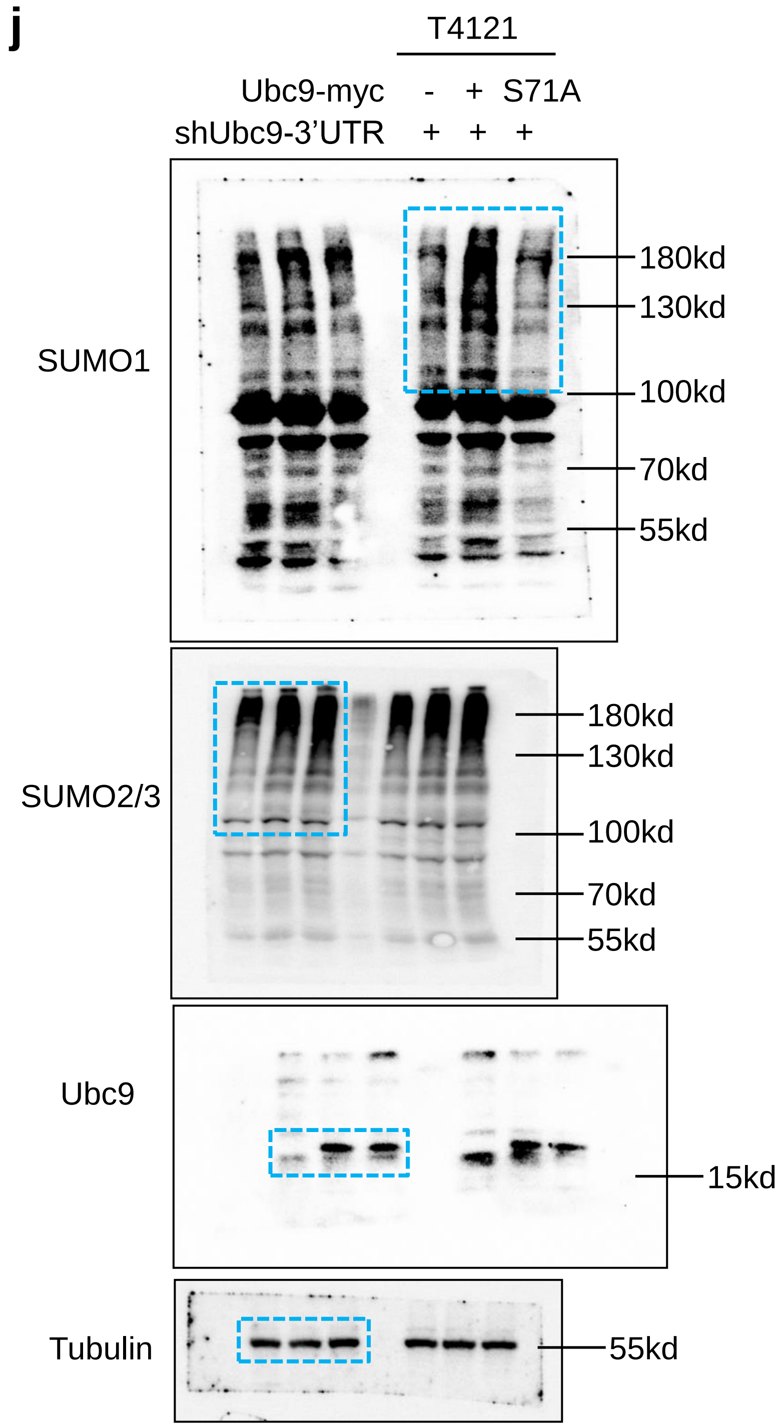

Source Data - Supplementary Figure 5

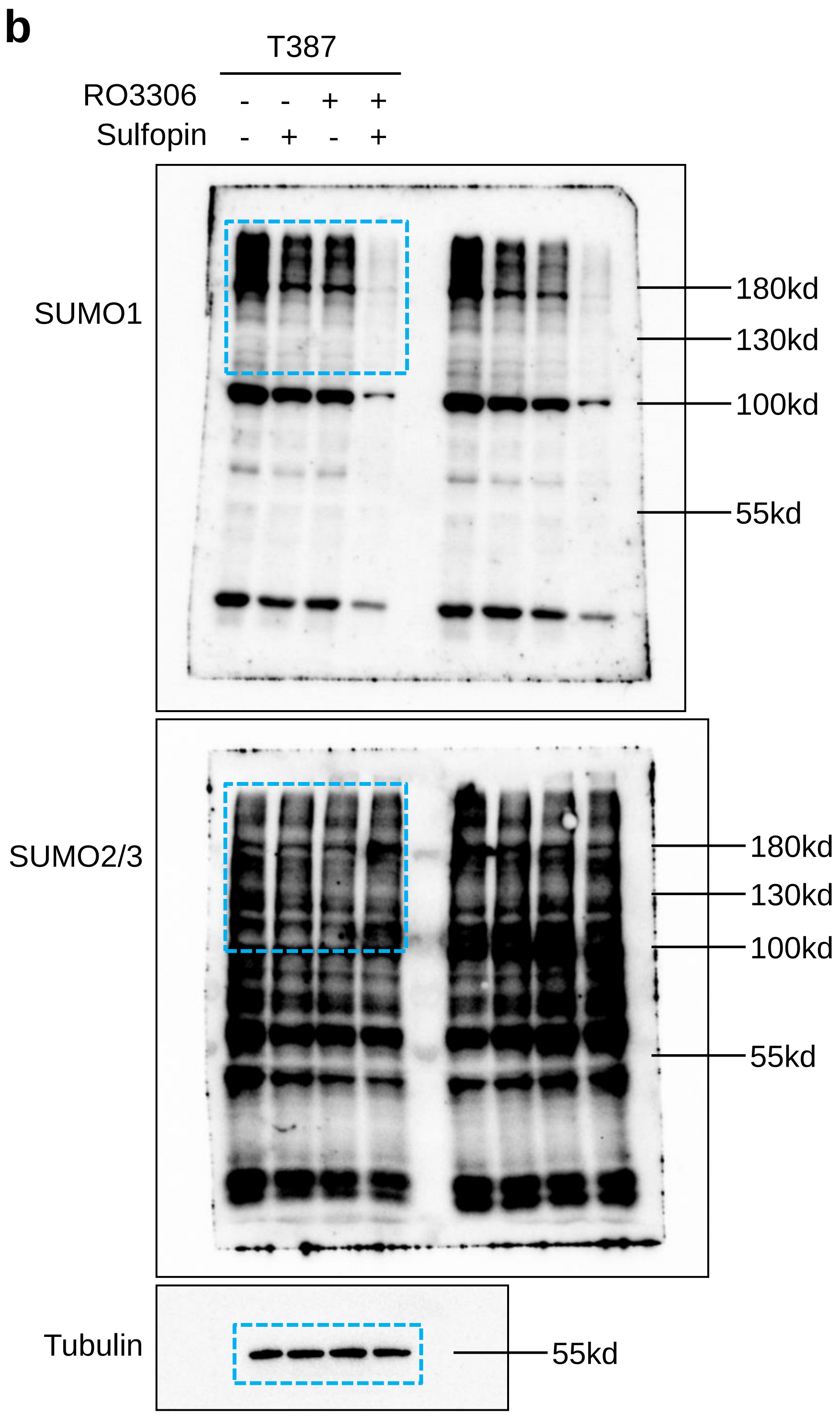

Source Data - Supplementary Figure 7

a

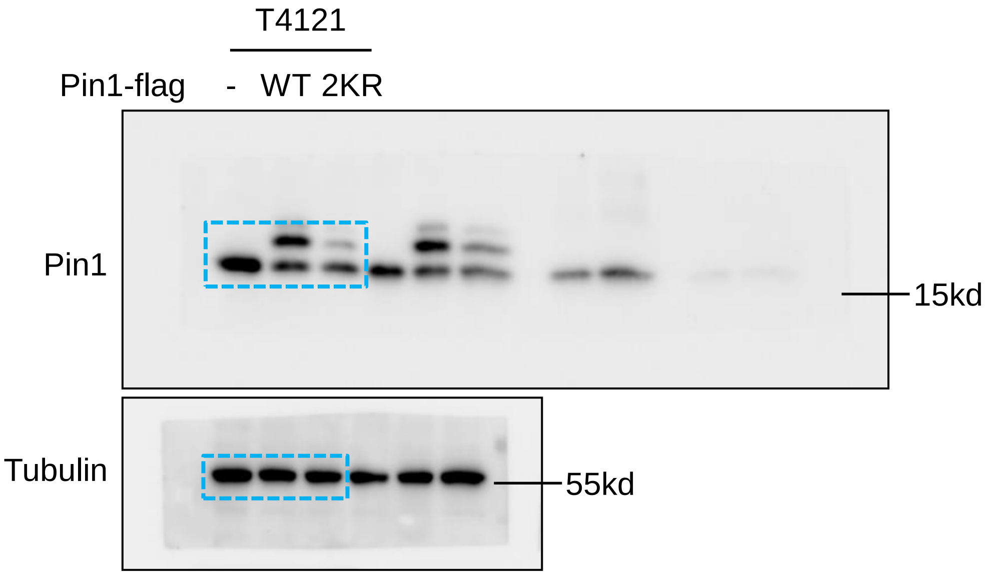

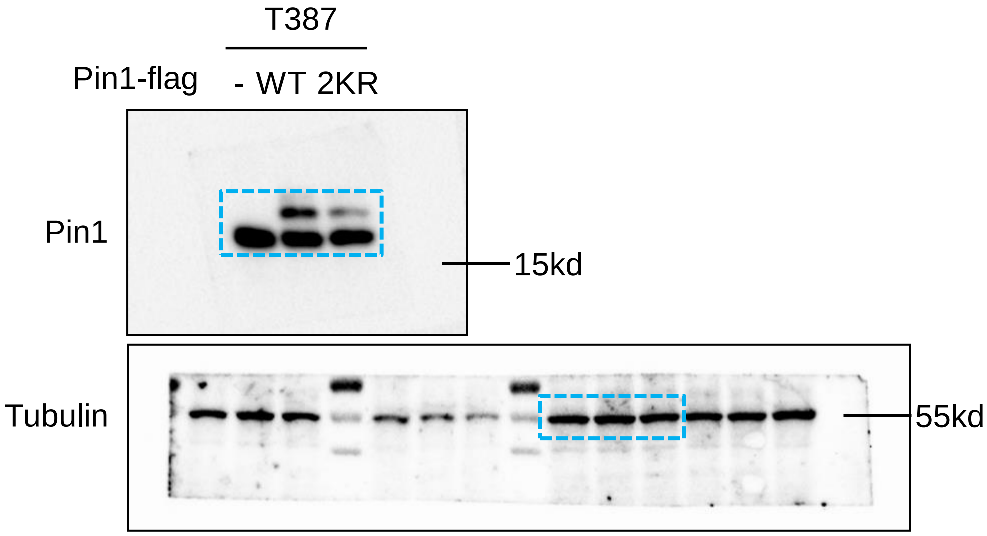

Source Data - Supplementary Figure 7

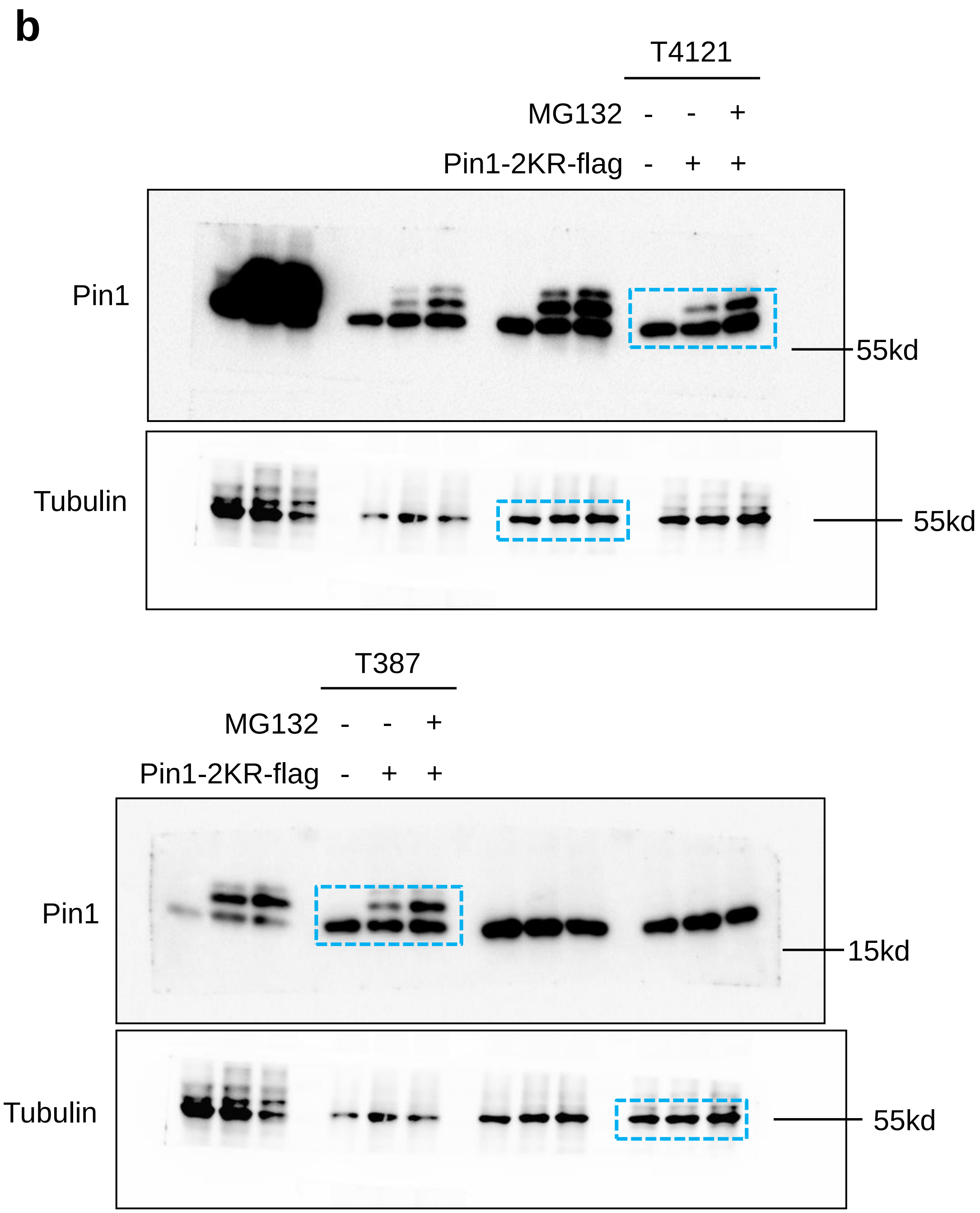

Source Data - Supplementary Figure 7

c

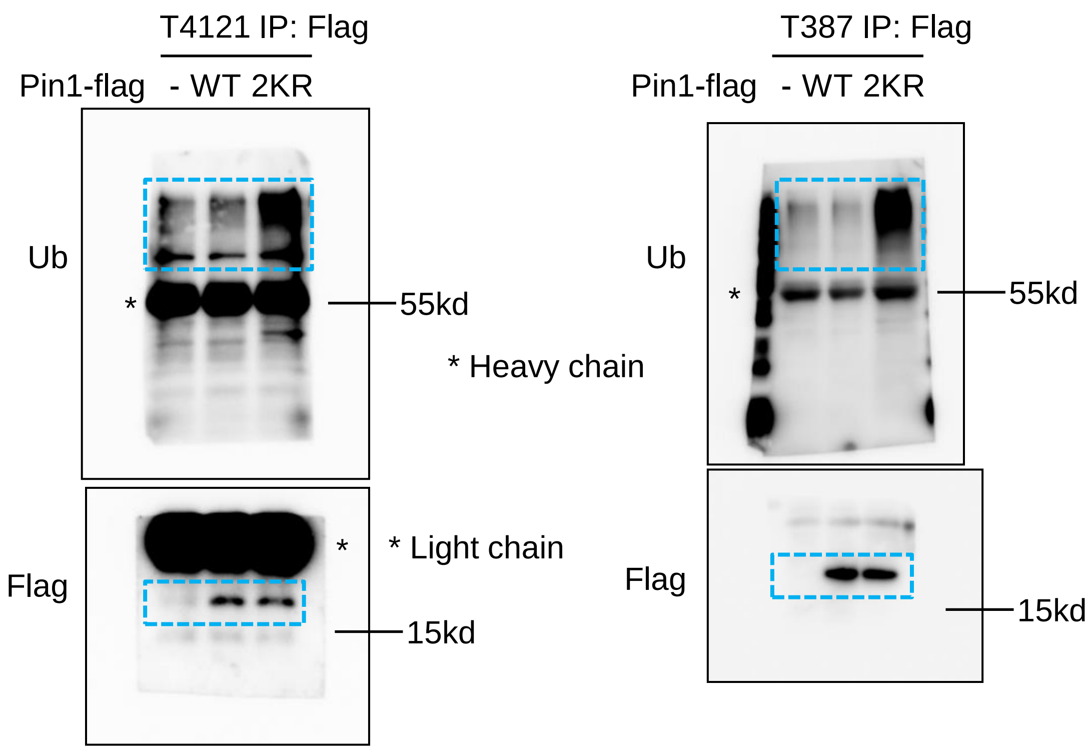

Source Data - Supplementary Figure 7

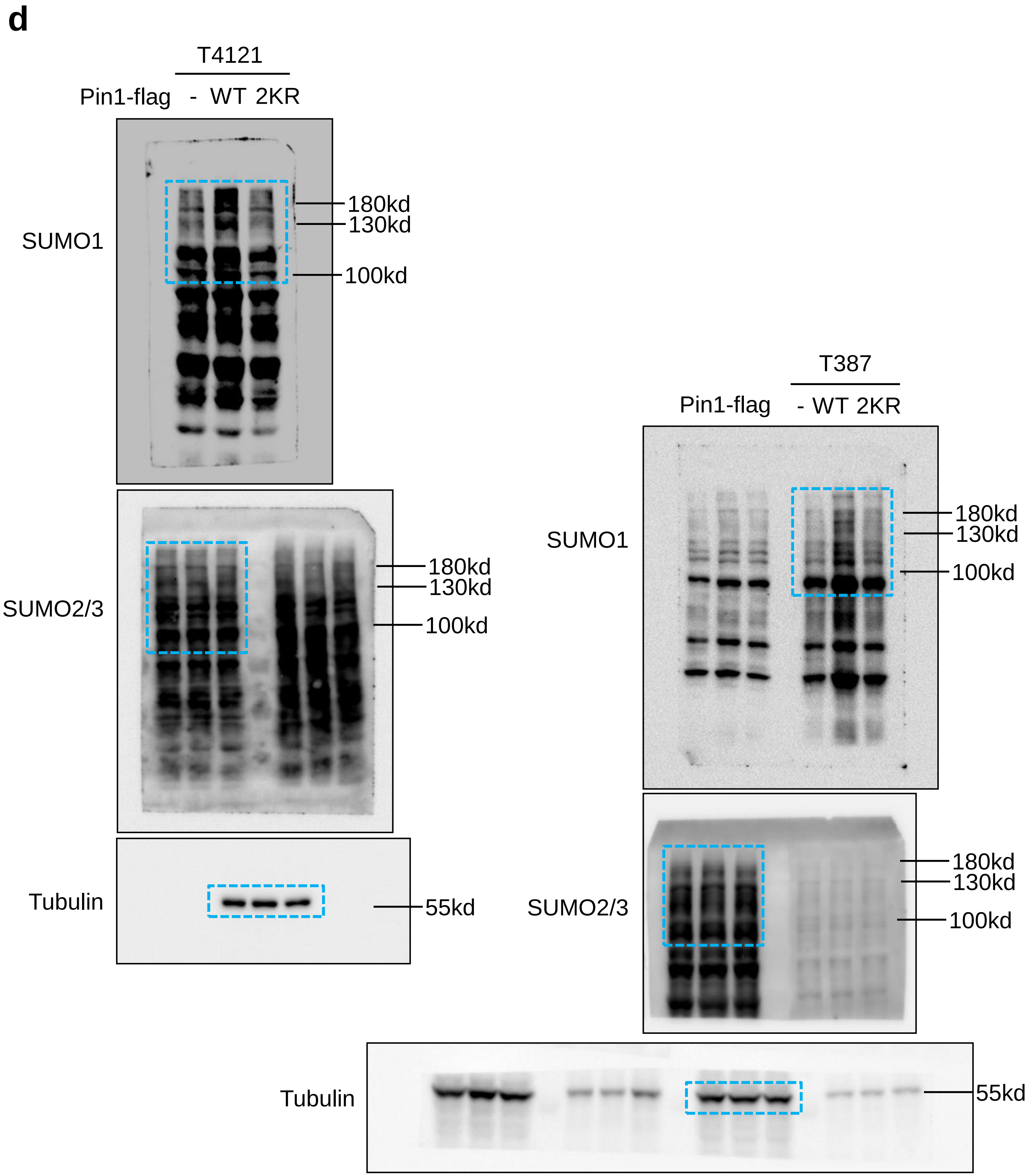

Source Data - Supplementary Figure 7

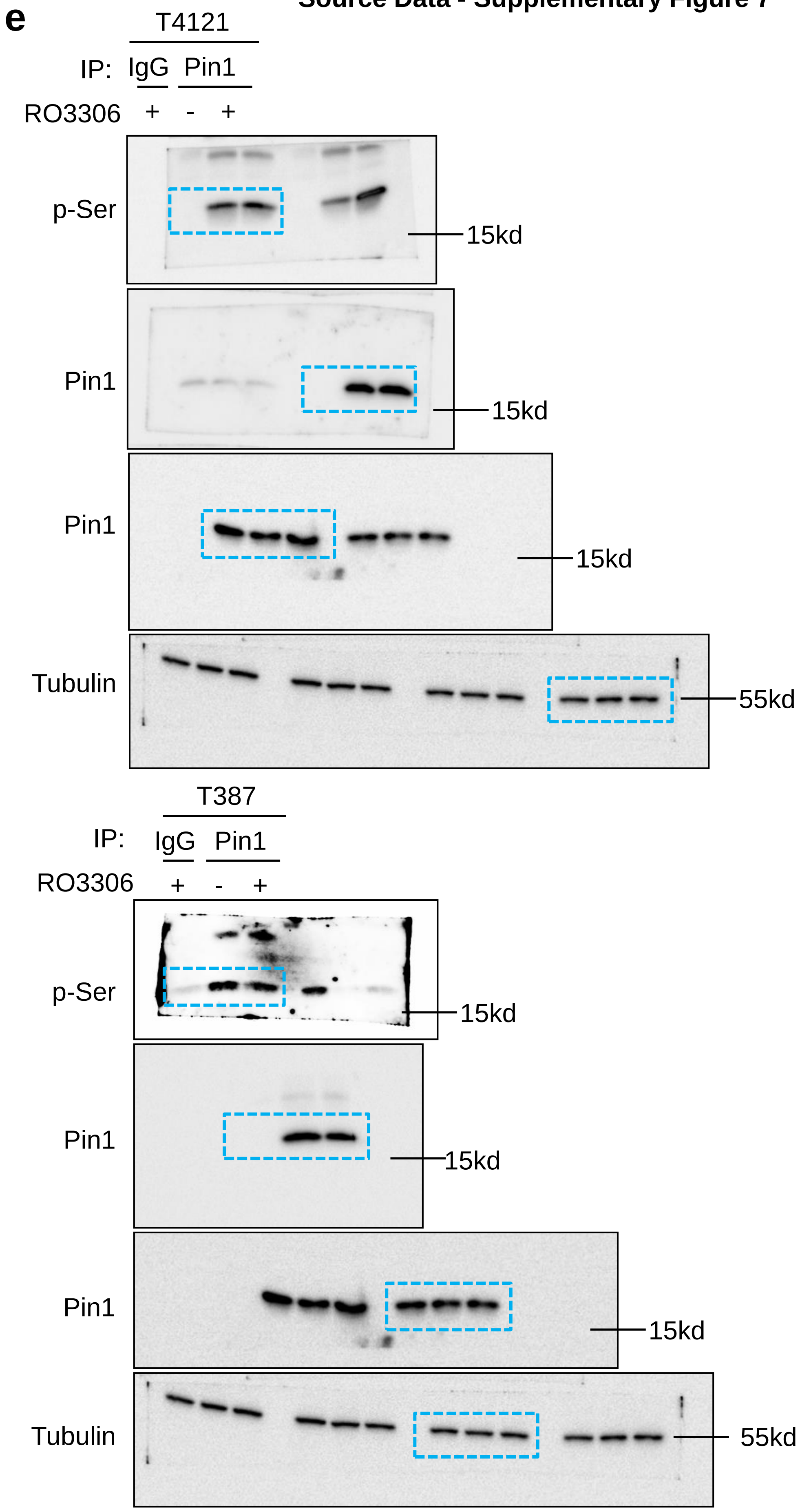

Source Data - Supplementary Figure 7

f

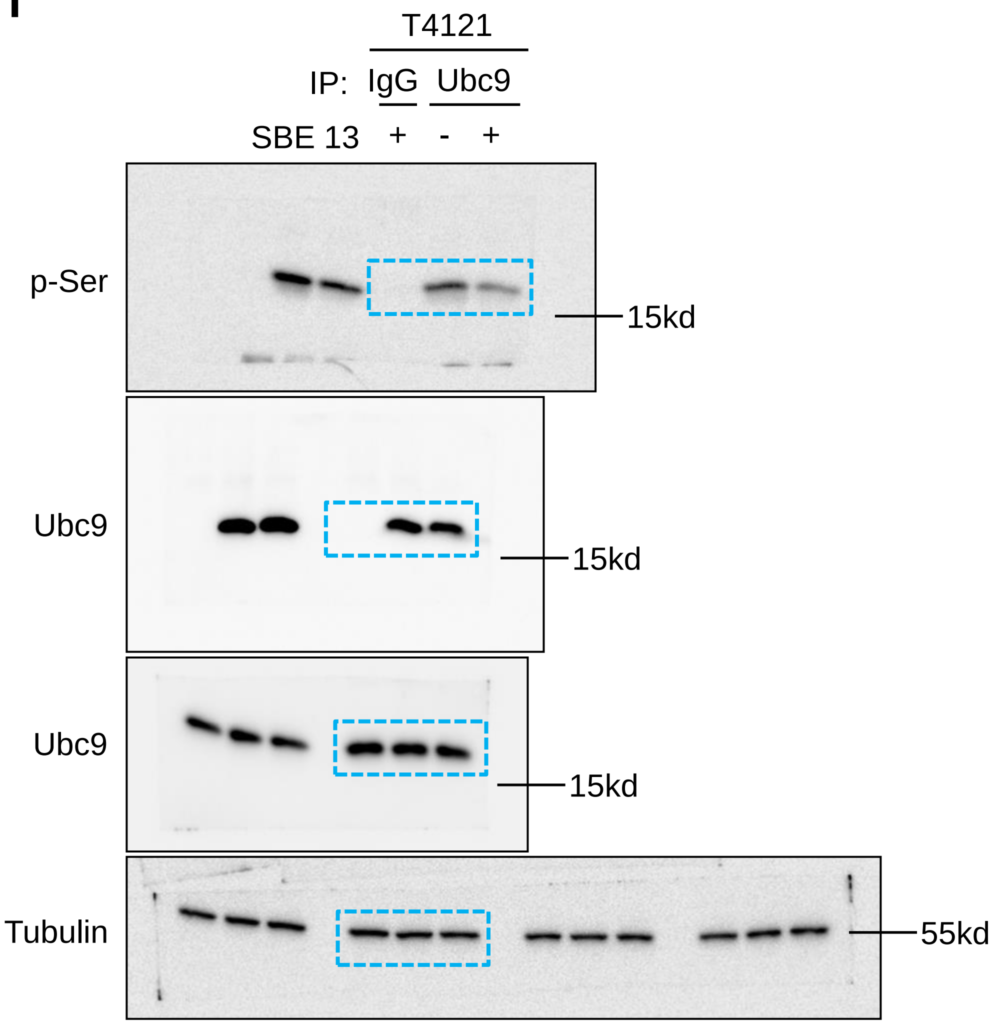

Source Data - Supplementary Figure 7

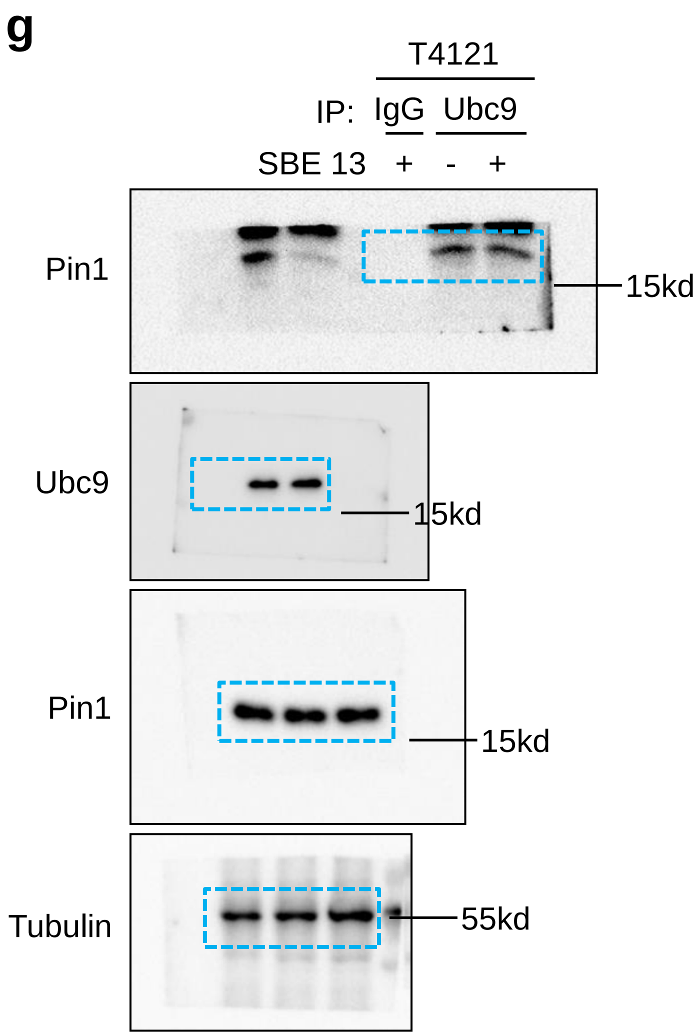

Supplement: Supplementary file 1 — Supplementary Information [file 41467_2023_44349_MOESM1_ESM.pdf]
