# Supplementary material for: Stabilization of Pin1 by USP34 promotes Ubc9 isomerization and protein sumoylation in glioma stem cells
Source: Nat Commun. 2024 Jan 2;15:40. doi: 10.1038/s41467-023-44349-x (PMC10762127; doi:10.1038/s41467-023-44349-x)
Supplement: Supplementary file 3 — Reporting Summary [file 41467_2023_44349_MOESM3_ESM.pdf]

nature portfolio

Corresponding author(s): Wenchao Zhou

Last updated by author(s): Nov 15, 2023

Reporting Summary

Nature Portfolio wishes to improve the reproducibility of the work that we publish. This form provides structure for consistency and transparency in reporting. For further information on Nature Portfolio policies, see our Editorial Policies and the Editorial Policy Checklist.

Statistics

For all statistical analyses, confirm that the following items are present in the figure legend, table legend, main text, or Methods section.

n/a	Confirmed	
☐	☒	The exact sample size (*n*) for each experimental group/condition, given as a discrete number and unit of measurement
☐	☒	A statement on whether measurements were taken from distinct samples or whether the same sample was measured repeatedly
☐	☒	The statistical test(s) used AND whether they are one- or two-sided<br>*Only common tests should be described solely by name; describe more complex techniques in the Methods section.*
☒	☐	A description of all covariates tested
☐	☒	A description of any assumptions or corrections, such as tests of normality and adjustment for multiple comparisons
☐	☒	A full description of the statistical parameters including central tendency (e.g. means) or other basic estimates (e.g. regression coefficient) AND variation (e.g. standard deviation) or associated estimates of uncertainty (e.g. confidence intervals)
☐	☒	For null hypothesis testing, the test statistic (e.g. *F*, *t*, *r*) with confidence intervals, effect sizes, degrees of freedom and *P* value noted<br>*Give P values as exact values whenever suitable.*
☒	☐	For Bayesian analysis, information on the choice of priors and Markov chain Monte Carlo settings
☒	☐	For hierarchical and complex designs, identification of the appropriate level for tests and full reporting of outcomes
☒	☐	Estimates of effect sizes (e.g. Cohen's *d*, Pearson's *r*), indicating how they were calculated

Our web collection on statistics for biologists contains articles on many of the points above.

Software and code

Policy information about availability of computer code

Data collection	Q-Exactive-plus mass spectrometer and Easy-nLC 1000 liquid chromatograph (Thermo Scientific) were used to collect proteomics data.<br>Western blot images were acquired by using Bio-Rad ChemiDocTM Imaging System.<br>qRT-PCR data was collected by using ROCHE LightCycler 96 Real Time PCR instrument.<br>Bioluminescent images were acquired by using IVIS Spectrum (PerkinElmer).<br>BIOTEK Synergy H1 was used to detect the absorbance of the released pNA (4-nitroaniline) at 390 nm.
Data analysis	MS/MS spectra were searched using MASCOT engine (Matrix Science, London, UK; version 2.2) against a nonredundant International Protein Index arabidopsis sequence database v3.85 (released at September 2011; 39679 sequences) from the European Bioinformatics Institute (http://www.ebi.ac.uk/).<br>LightCycler® 96 SW 1.1 software was used to analyze qRT-PCR data.<br>Bioluminescent images was analyzed by using Living Image® 4.5.5 (64-bit).<br>Data analysis,statistical testing and visualization were conducted in Prism 6 software (GraphPad Software).

For manuscripts utilizing custom algorithms or software that are central to the research but not yet described in published literature, software must be made available to editors and reviewers. We strongly encourage code deposition in a community repository (e.g. GitHub). See the Nature Portfolio guidelines for submitting code & software for further information.

Data

Policy information about availability of data

All manuscripts must include a data availability statement. This statement should provide the following information, where applicable:

- Accession codes, unique identifiers, or web links for publicly available datasets
- A description of any restrictions on data availability
- For clinical datasets or third party data, please ensure that the statement adheres to our policy

The mass spectrometry proteomics data generated in this study have been deposited to the ProteomeXchange Consortium via the PRIDE partner repository with the dataset identifier PXD041043 (https://proteomecentral.proteomexchange.org/cgi/GetDataset?ID=PXD041043). The datasets generated during and/or analyzed during the current study are available are provided within the Article, Supplementary information or Source Data file. All other data supporting the results of this study can be obtained from the corresponding author upon reasonable request.

Research involving human participants, their data, or biological material

Policy information about studies with human participants or human data. See also policy information about sex, gender (identity/presentation), and sexual orientation and race, ethnicity and racism.

Reporting on sex and gender	Our findings apply to both sexes. Gender was not considered in our study design. Disaggregated sex and gender information has not been collected.
Reporting on race, ethnicity, or other socially relevant groupings	There is no socially constructed or socially relevant categorization variables used in the manuscript.
Population characteristics	Tumor tissue samples from patients diagnosed with GBM were used for immunofluorescence experiments. These GBM patients include 3 males and 3 females aged 43-66 years. This research was not age-specific or sex-specific.
Recruitment	The GBM patients were admitted to the First Affiliated Hospital of the University of Science and Technology of China for surgery during 2020 to 2021 because of the self-reported neurological symptoms followed by the diagnosis of GBM in the hospital. The surgically removed tumor tissues were applied in this study.
Ethics oversight	The study was approved by the Medical Research Ethics Committee of The First Affiliated Hospital of the University of Science and Technology of China.

Note that full information on the approval of the study protocol must also be provided in the manuscript.

Field-specific reporting

Please select the one below that is the best fit for your research. If you are not sure, read the appropriate sections before making your selection.

☒ Life sciences ☐ Behavioural & social sciences ☐ Ecological, evolutionary & environmental sciences

For a reference copy of the document with all sections, see nature.com/documents/nr-reporting-summary-flat.pdf

Life sciences study design

All studies must disclose on these points even when the disclosure is negative.

Sample size	No sample-size calculation was performed. The sample sizes were chosen based on previous publications using similar sample sizes in the GBM and GSC field from laboratories of Dr. Shideng Bao, Dr. Jeremy N Rich, and other researchers. The sample sizes were enough for the detection of biological outcomes, and statistical significance was determined through performing biologically independent repeats.
Data exclusions	Data were not excluded from analysis.
Replication	The number of independent biological repeats performed for each experiment are indicated through the manuscript main content, methods, and figure legends. Animal experiments were performed on at least n=5 mice per group for each experiment. All attempts of replication were successful and gave similar results.
Randomization	Mice were randomly allocated to control group or treatment groups. For in vitro experiments, randomization was not required as the samples are derived from defined groups. All samples were treated in the same way to decrease the variability.
Blinding	Data collection and analysis were not blinded to the operator. Because the experiments are mostly designed and performed by the same investigator, blinding is not possible.

Reporting for specific materials, systems and methods

We require information from authors about some types of materials, experimental systems and methods used in many studies. Here, indicate whether each material, system or method listed is relevant to your study. If you are not sure if a list item applies to your research, read the appropriate section before selecting a response.

Materials & experimental systems

n/a	Involved in the study
☐	☒ Antibodies
☐	☒ Eukaryotic cell lines
☒	☐ Palaeontology and archaeology
☐	☒ Animals and other organisms
☒	☐ Clinical data
☒	☐ Dual use research of concern
☒	☐ Plants

Methods

n/a	Involved in the study
☒	☐ ChIP-seq
☒	☐ Flow cytometry
☒	☐ MRI-based neuroimaging

Antibodies

Antibodies used

Antibodies for western blot:
Pin1 (Invitrogen, cat: PAS-80902, lot: 35C36A01, 1:1000; Proteintech, cat: 10495-1-AP, lot: 00106419, 1:1000)
Ubc9 (Abcam, cat: ab33044, lot: 1015896-1, 1:1000)
USP34 (Santa cruz biotechnology, cat: sc-100631, lot: #L1321, clone name: 3H9, 1:500; Bethyl Laboratories, cat: A300-824A, lot: #1, 1:1000)
HA (Sigma, cat: 11867423001, clone name: 3F10, 1:2500)
tubulin (Sigma-Aldrich, cat: T9026, clone name: DM1A, 1:5000)
Flag (Sigma-Aldrich, cat: F1804, lot: 102618326, clone name: M2, 1:2500)
CDK1 (Proteintech, cat: 19532-1-AP, 1:1000)
Plk1 (Santa cruz biotechnology, cat: sc-17783, lot: #D1320, clone name: F-8, 1:1000)
ubiquitin (Proteintech, cat: 10201-2-AP, lot: 00103247, 1:1000)
pSer (SIGMA-Aldrich, cat: 05-1000X, clone name: 4A4, 1:1000; Santa cruz biotechnology, cat: sc-81514, lot: #H0123, clone name: 16B4, 1:500)
SUMO1 (Invitrogen, cat: 33-2400, clone name: 21C7, lot: VC298935, 1:1000; Cell Signaling Technology, cat: 4930S, lot: 5, 1:1000)
SUMO2/3 (MBL, cat: M114-3, lot: 028, clone name: 1E7,1:1000)
SOX2 (Cell Signaling Technology, cat: 3579, lot: 8, clone name: D6D9, 1:2000)
Myc tag (Proteintech, cat: 16286-1-AP, lot: 00132370, 1:1000)
goat anti-rabbit IgG (H+L) secondary antibody, HRP (Thermo, cat: 31460, XF348801, 1:5000)
goat anti-mouse IgG (H+L) secondary antibody, HRP (Thermo, cat: 31430, lot: XH363702, 1:5000)
goat anti-rat IgG (H+L) secondary antibody, HRP (Thermo, cat: 31470, 1:5000)
Antibodies for immunoprecipitation:
Pin1 (Invitrogen, cat: PAS-80902, lot: 35C36A01)
Ubc9 (Abcam, cat: ab33044, lot: 1015896-1)
normal rabbit IgG (CST, #2729)
Anti-DYKDDDDK-Tag Mouse Antibody (Agarose Conjugated) (Abmart, cat: M20018M, lot: 344672)
Anti-MYC Tag mAb conjugated protein A agarose (Abmart, cat: M20030M, lot: 354168)
Antibodies for Immunofluorescent staining:
Pin1 (Invitrogen, cat: PAS-80902, lot: 35C36A01, 1:200)
USP34 (Santa cruz biotechnology, cat: sc-100631, lot: #L1321, clone name: 3H9, 1:50; Bethyl Laboratories, cat: A300-824A, lot: #1, 1:200)
SOX2 (R&D Systems, cat: AF2018, lot: K0Y0419101, 1:300; Cell Signaling Technology, cat: 3579, lot: 8, clone name: D6D9, 1:300)
Olig2 (R&D Systems, cat: AF2418, lot: LPA0719071, 1:300)
SUMO1 (Cell Signaling Technology, cat: 4940, lot: 5, 1:200)
SUMO2/3 (MBL, cat: M114-3, lot: 028, clone name: 1E7, 1:200)
cleaved caspase-3 (Cell Signaling Technology, cat: 9661S, lot: 22, clone name: 5A1E, 1:300)
Ki67 (abcam, cat: ab15580, lot: GR3452679-1, 1:300)
GFAP (BioLegend, cat: 840001, lot: B292266, 1:300)
NeuN (abcam, cat: ab177487, lot: 1002496-1, clone name: EPR12763, 1:500)
Donkey anti-rabbit Alexa Fluor 488 (invitrogen, cat: A-21206, lot: 2376850, 1:1000)
Donkey anti-rabbit Alexa Fluor 568 (invitrogen, cat: A-10042, lot: 2044343, 1:1000)
Donkey anti-mouse Alexa Fluor 488 (invitrogen, cat: A-21202, lot: 2428531, 1:1000)
Donkey anti-mouse Alexa Fluor 568 (invitrogen, cat: A-10037, lot: 2300930, 1:1000)
Donkey anti-goat Alexa Fluor 488 (invitrogen, cat: A-11055, lot: 2134018, 1:1000)
Donkey anti-goat Alexa Fluor 568 (invitrogen, cat: A-11057, lot: 2160061, 1:1000)

Validation

All antibodies are commercially available and have been validated for the indicated application by the manufacturers.

Eukaryotic cell lines

Policy information about cell lines and Sex and Gender in Research

Cell line source(s)	T387, T4121, H2S and T3832 GSCs were kind gifts from Dr. Jeremy Rich (University of Pittsburgh).<br>Human HEK293T was purchased from ATCC.
Authentication	Cell lines were authenticated by STR profiling.
Mycoplasma contamination	Cells were routinely tested and confirmed negative for mycoplasma by using PCR detection method.
Commonly misidentified lines<br>(See ICLAC register)	No commonly misidentified cell lines were used in this study.

Animals and other research organisms

Policy information about studies involving animals; ARRIVE guidelines recommended for reporting animal research, and Sex and Gender in Research

Laboratory animals	6-8 weeks old nude mice (Shanghai SLAC Laboratory Animal) were used for the orthotopic GBM xenograft studies.
Wild animals	Wild animals were not used in this study.
Reporting on sex	Our findings apply to both sexes. Sex was not considered in study design.
Field-collected samples	This study did not involve samples collected from the field.
Ethics oversight	All animal protocols were approved by the Animal Research Ethics Committee of the University of Science and Technology of China (USTC), and all animal experiments were performed in accordance to the USTC guidelines for the use of laboratory animals.<br>Collection and use of human GBM specimens were approved by the Medical Research Ethics Committee of The First Affiliated Hospital of the University of Science and Technology of China.

Note that full information on the approval of the study protocol must also be provided in the manuscript.

Plants

Seed stocks	Not applicable
Novel plant genotypes	Not applicable
Authentication	Not applicable
