# Supplementary figures and images for: Stabilization of Pin1 by USP34 promotes Ubc9 isomerization and protein sumoylation in glioma stem cells
Source: Nat Commun. 2024 Jan 2;15:40. doi: 10.1038/s41467-023-44349-x (PMC10762127; doi:10.1038/s41467-023-44349-x)

**a**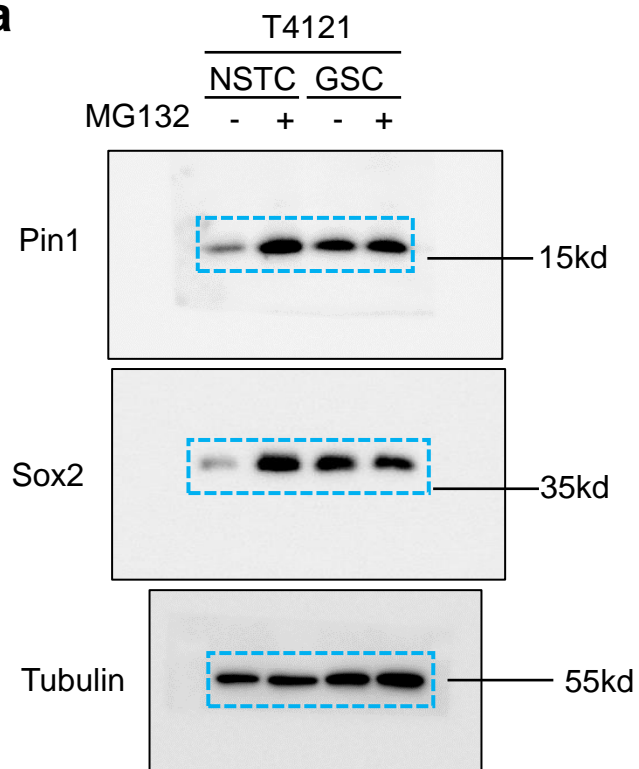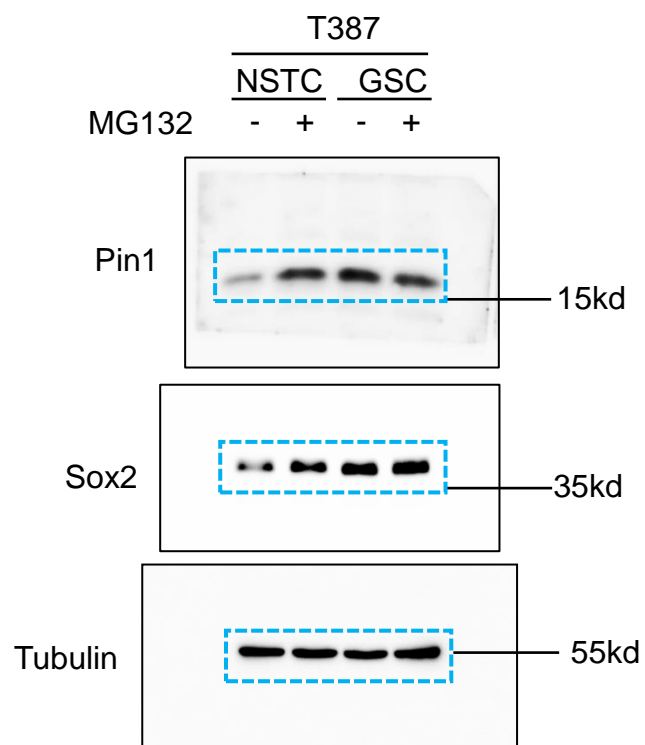**b**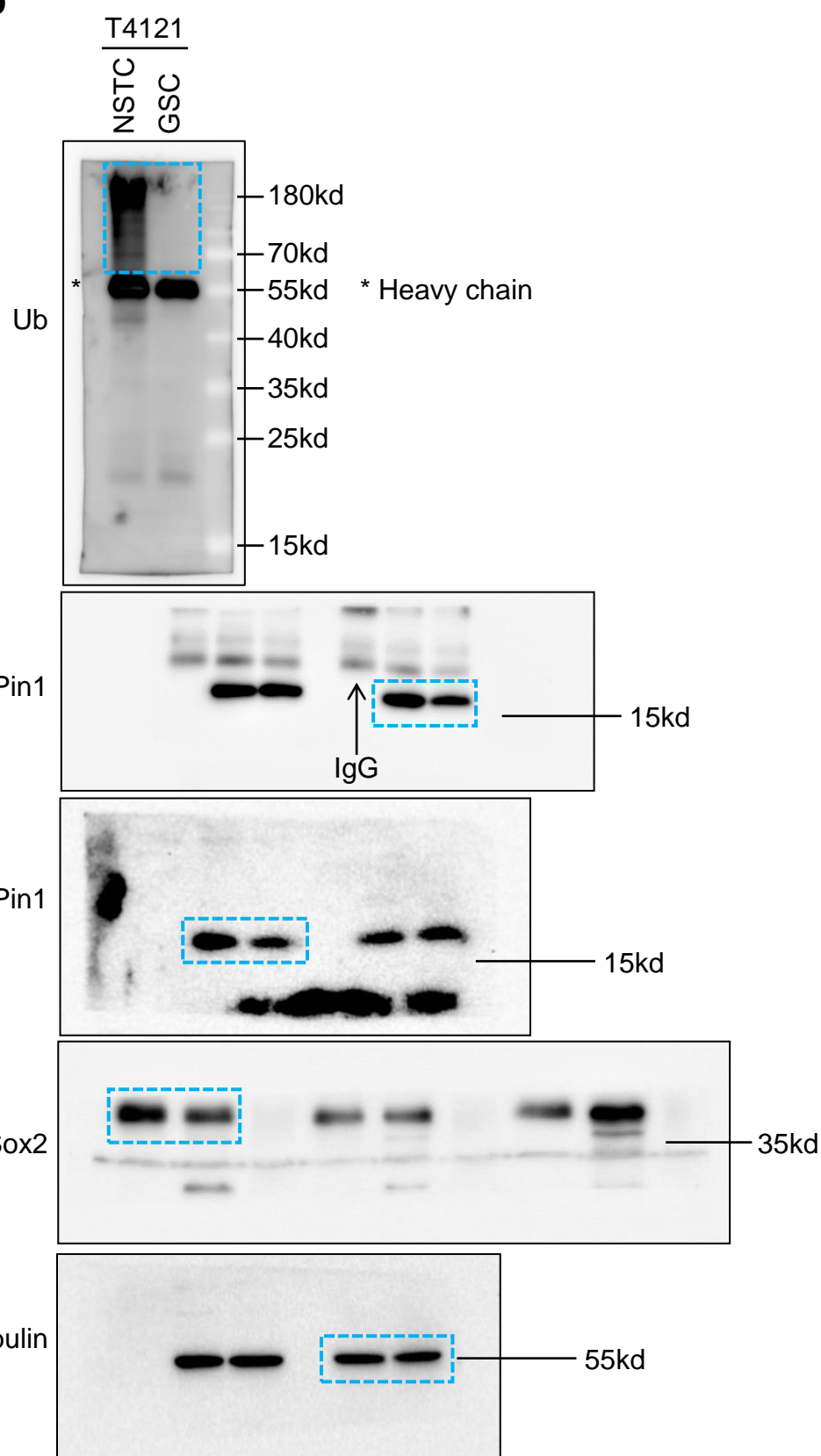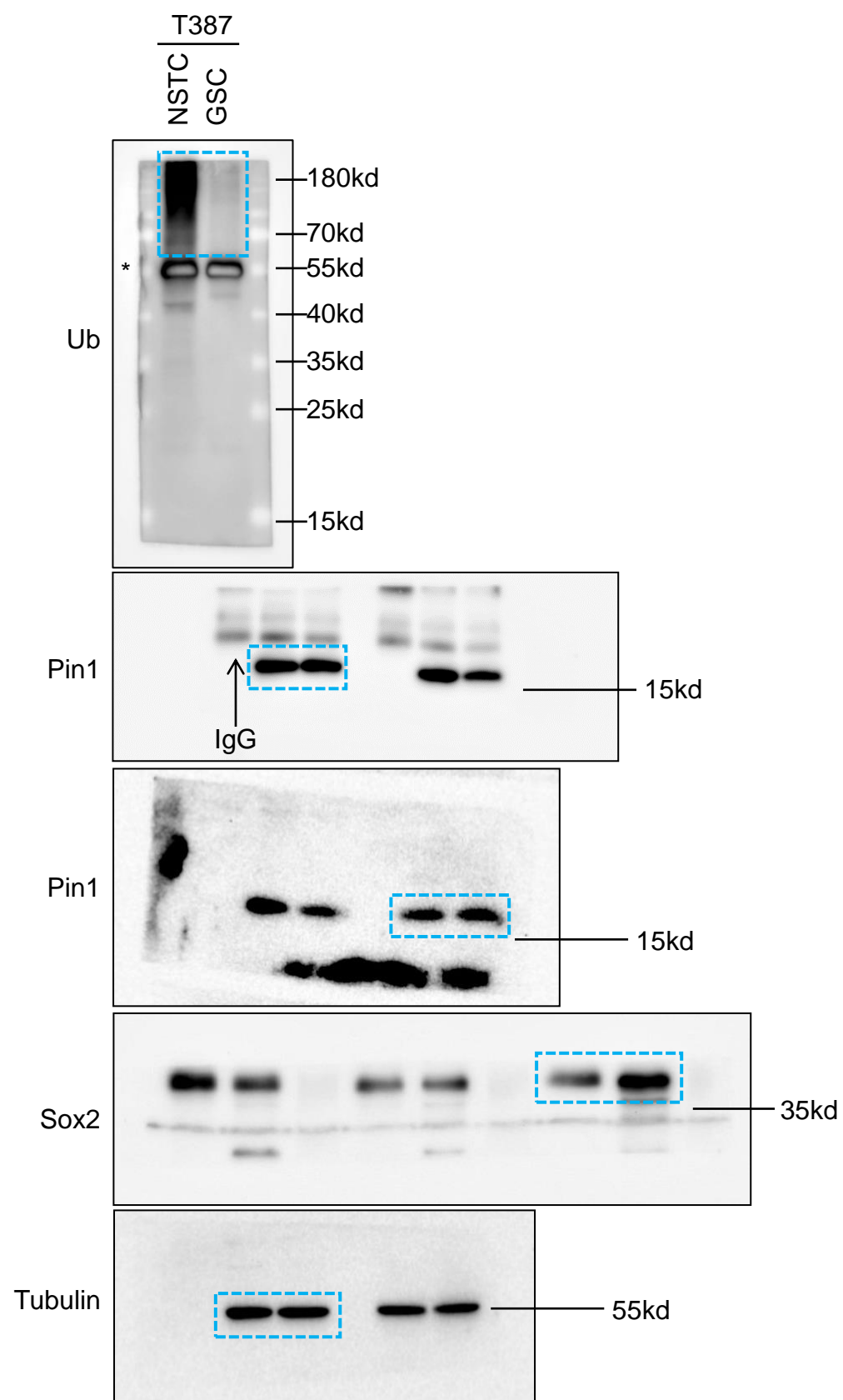

**d**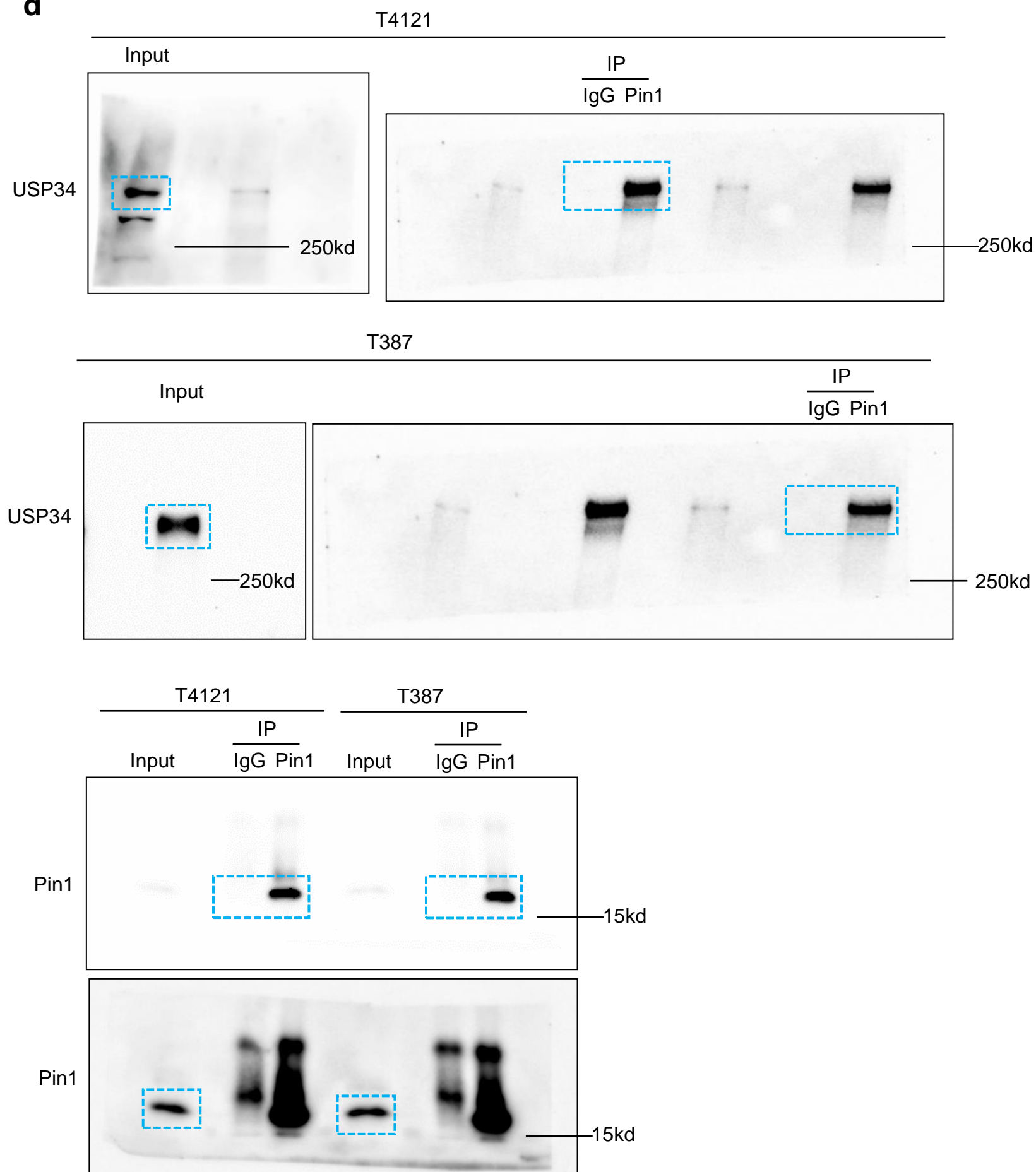

**g**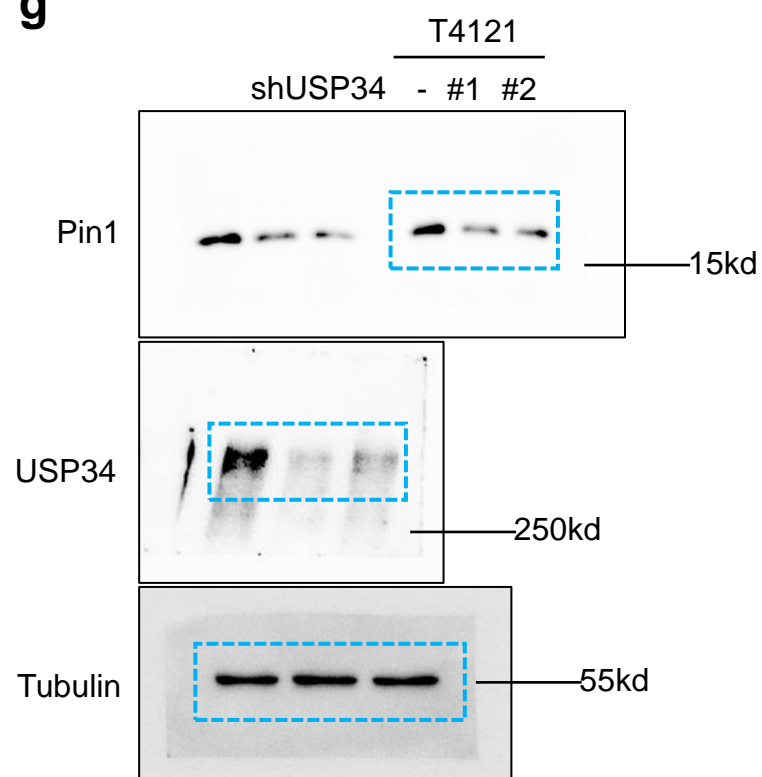**h**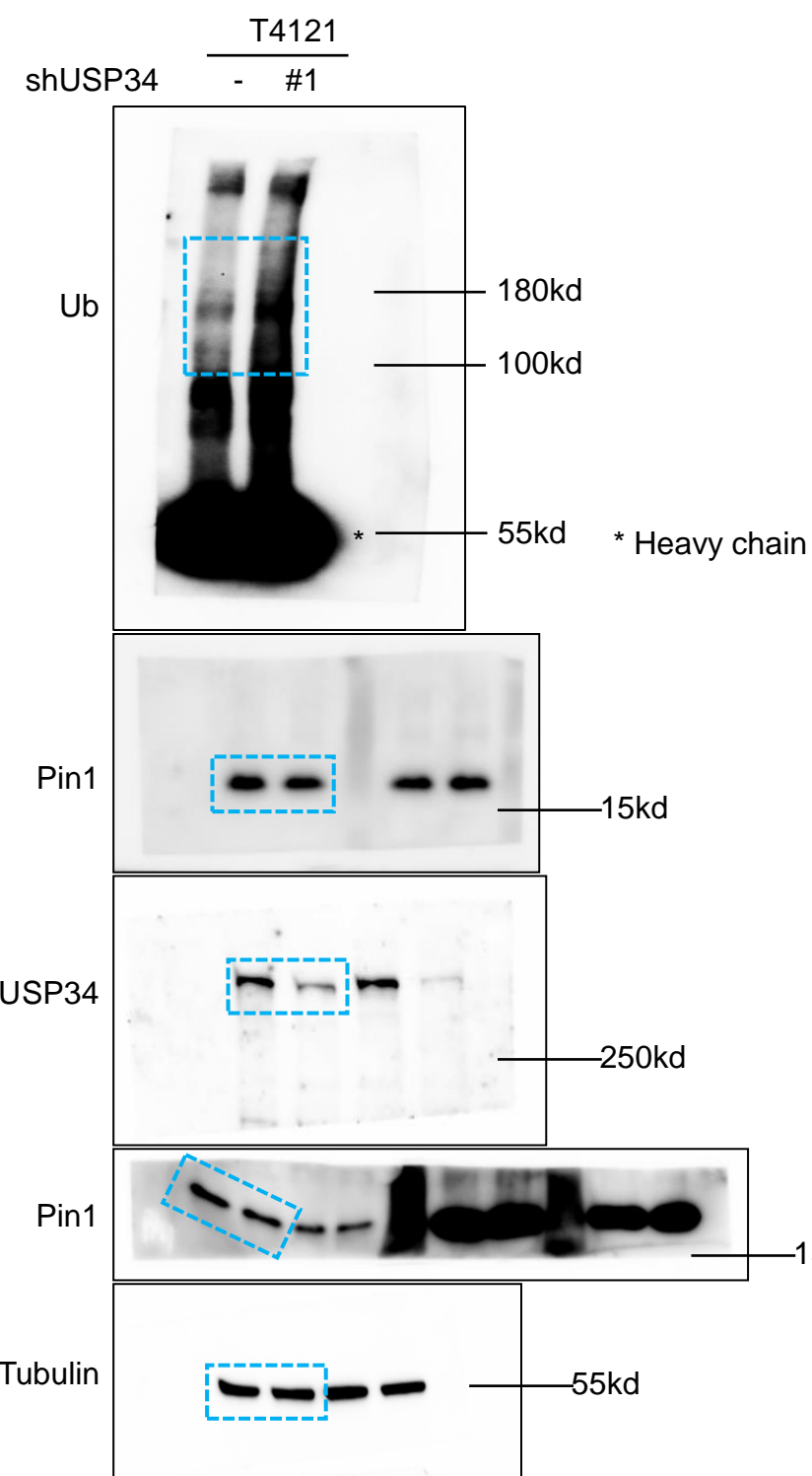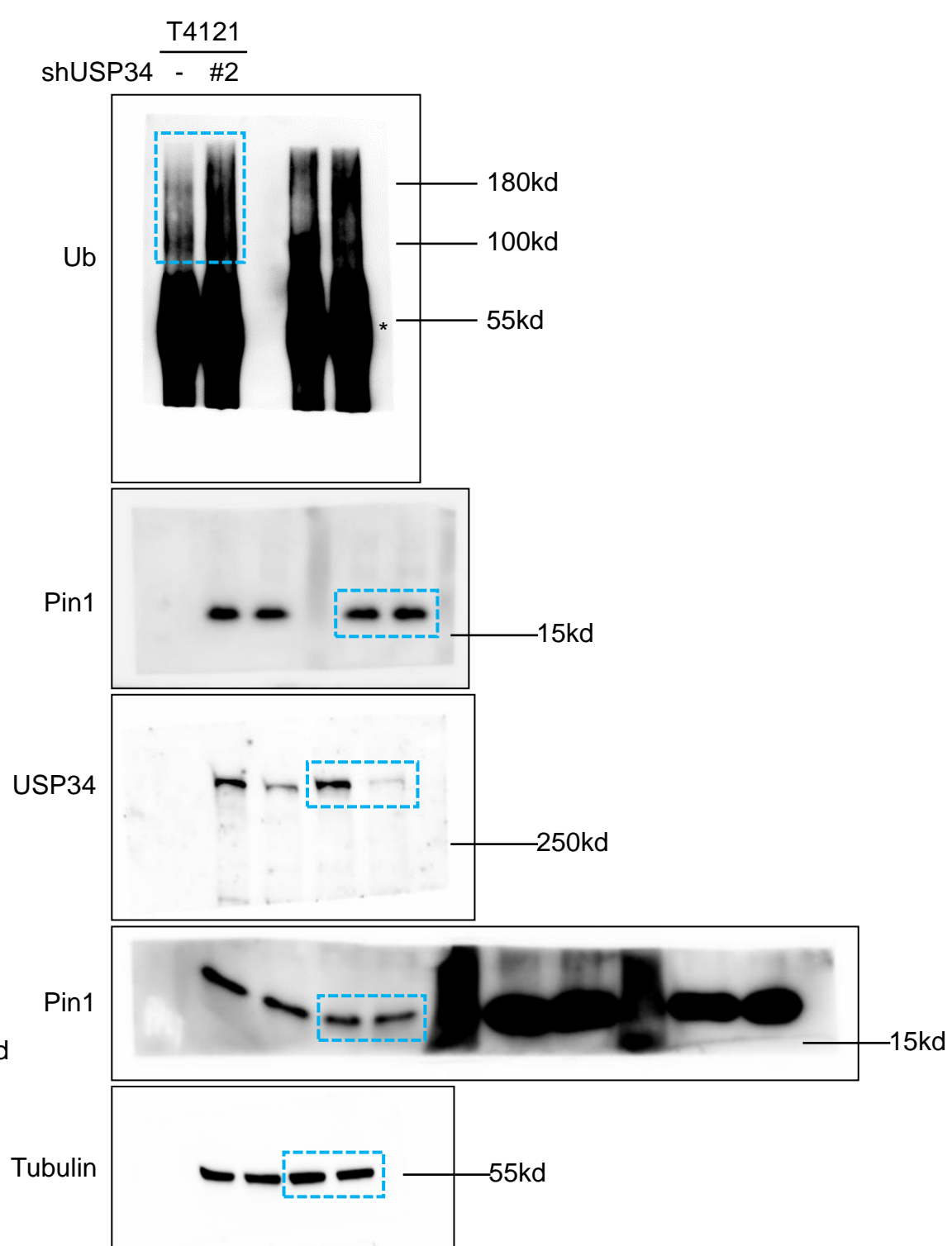

**j**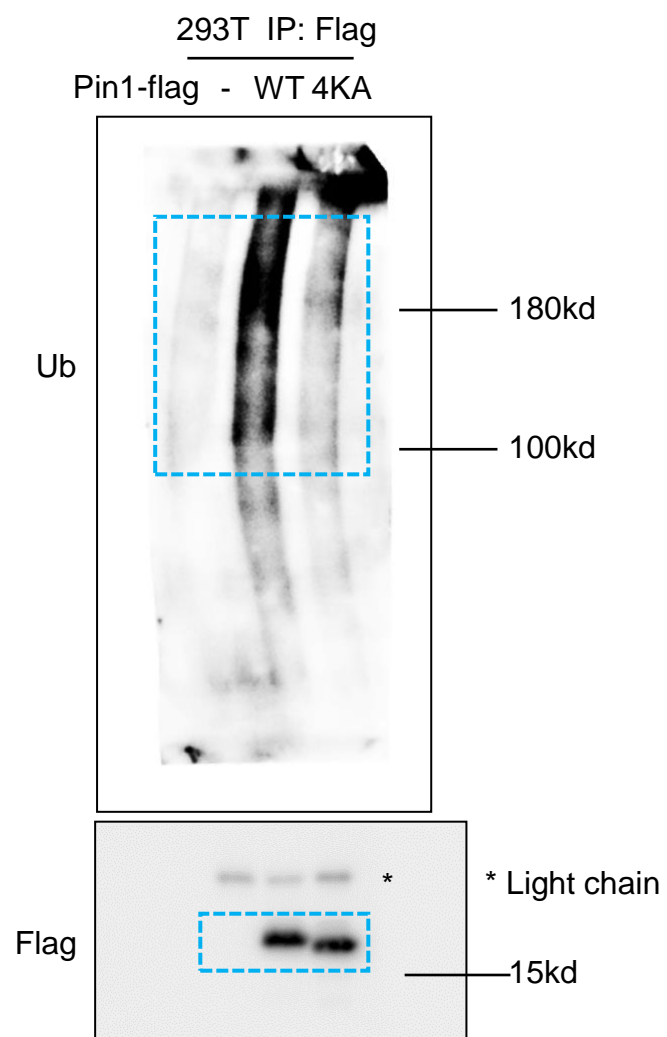**k**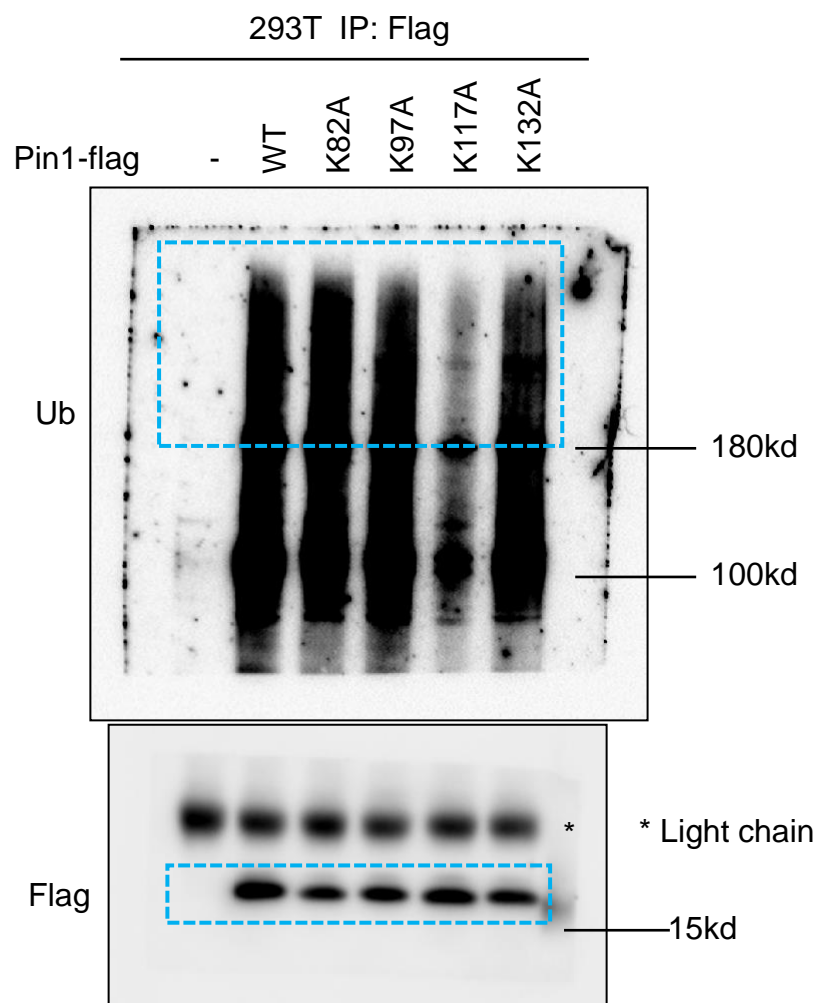**l**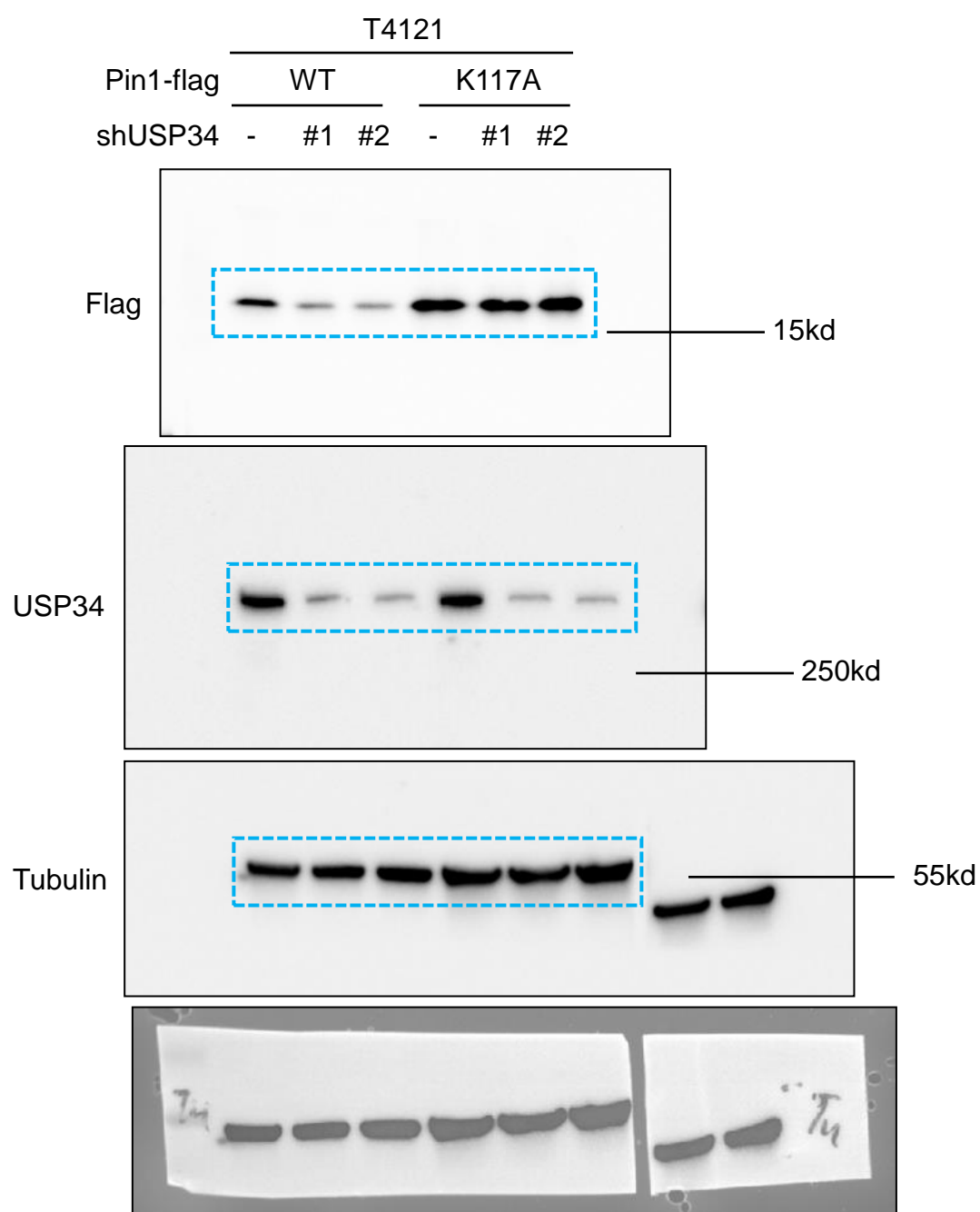

**a**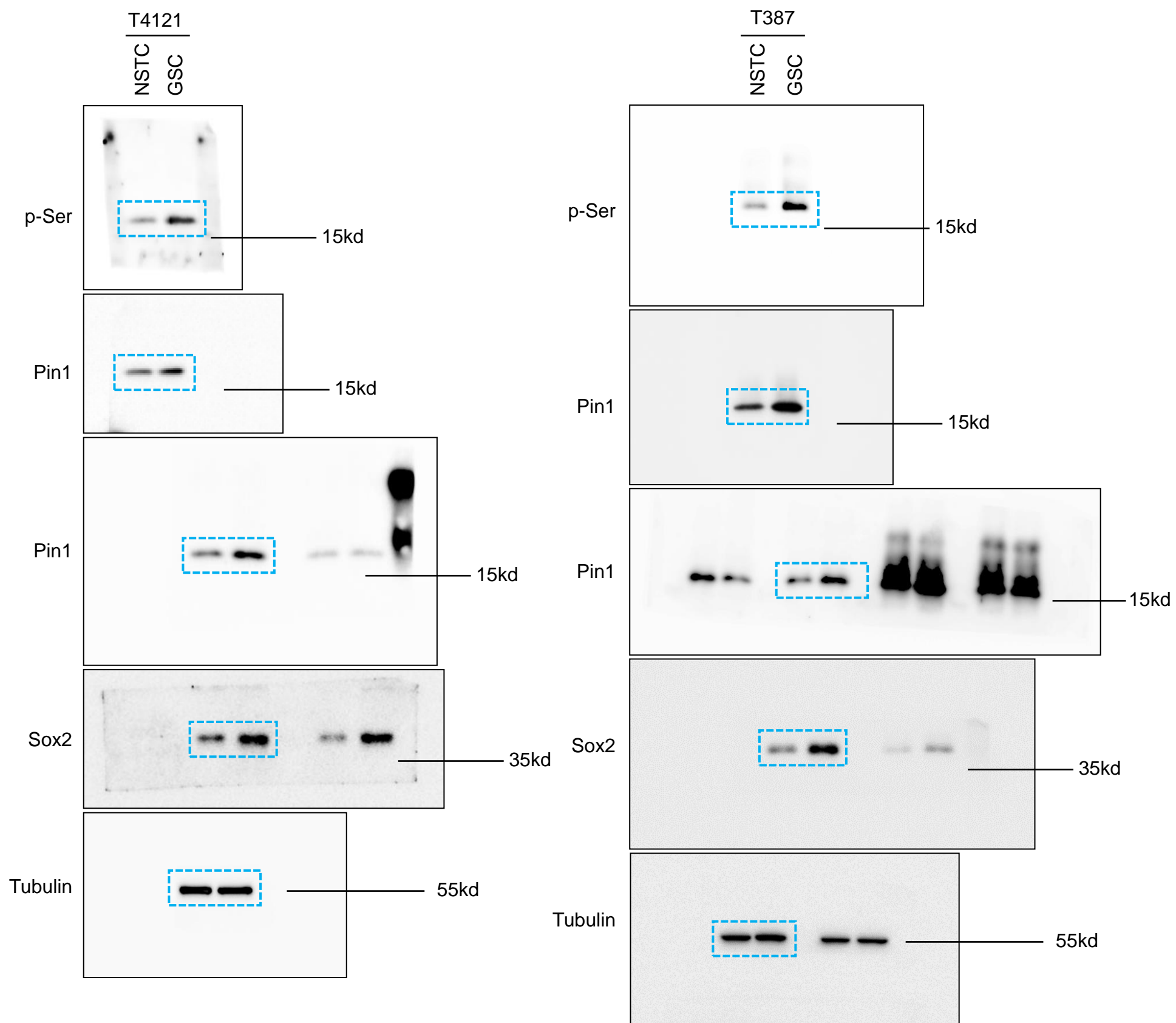

**b**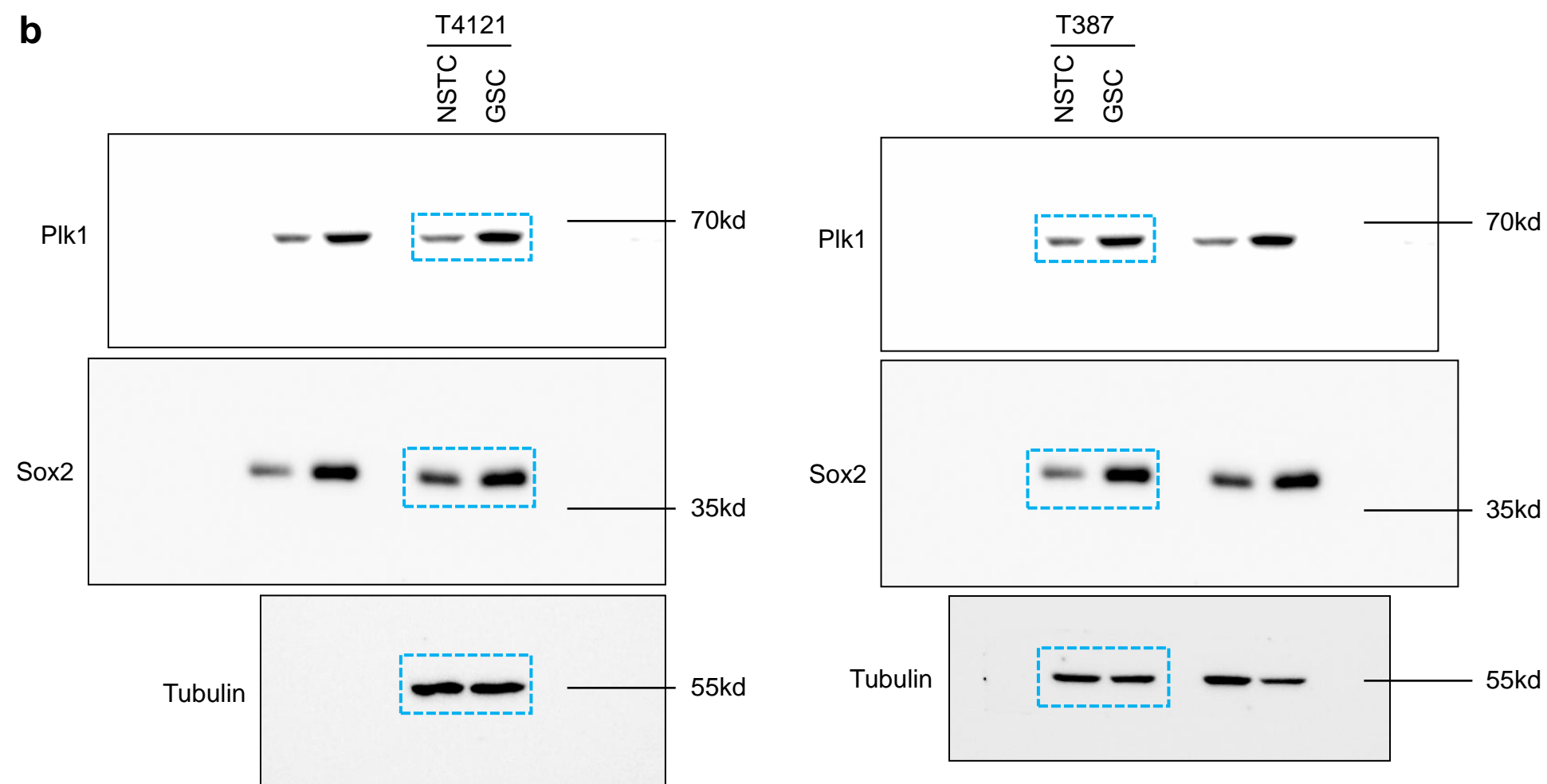**c**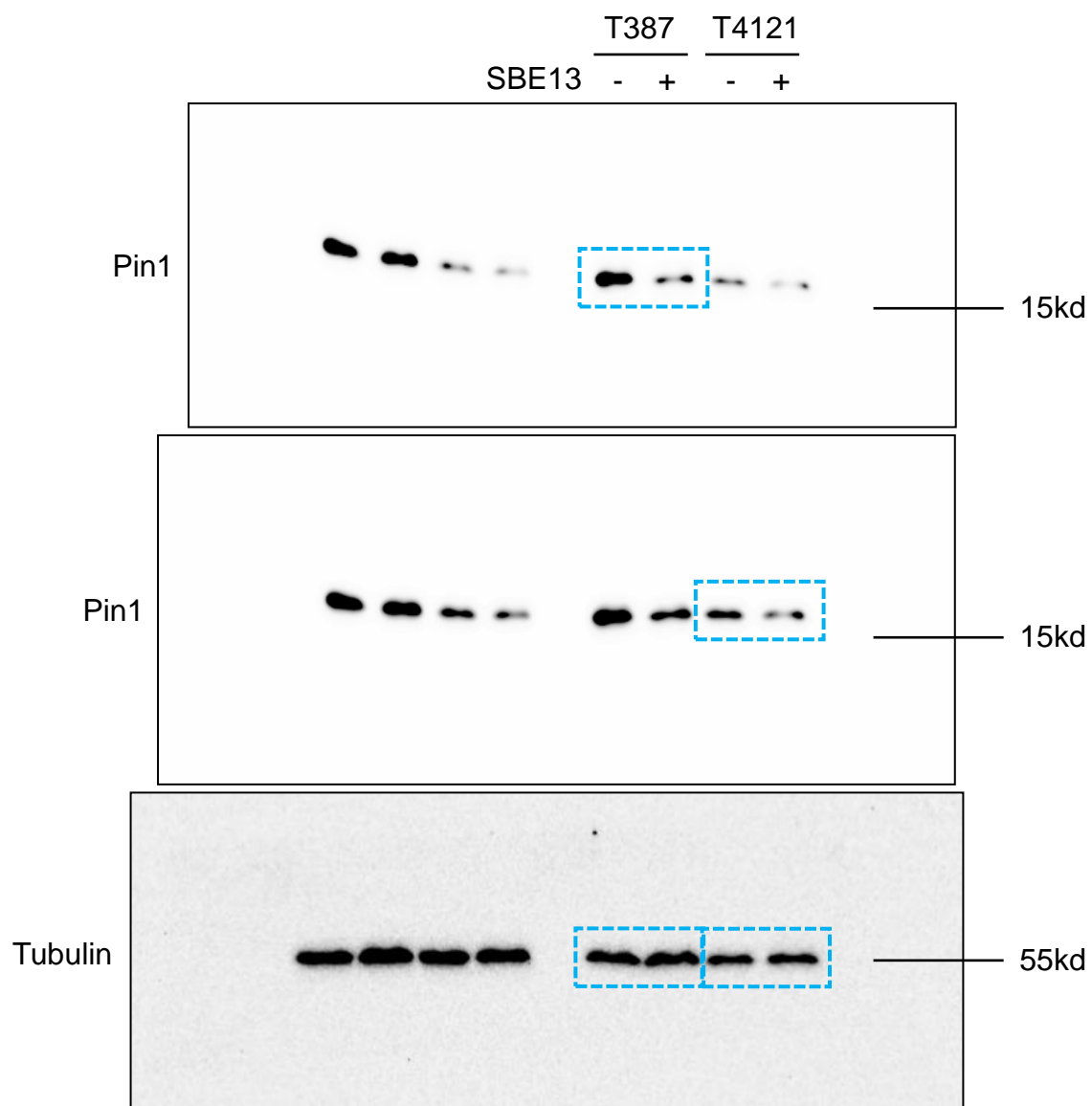

d

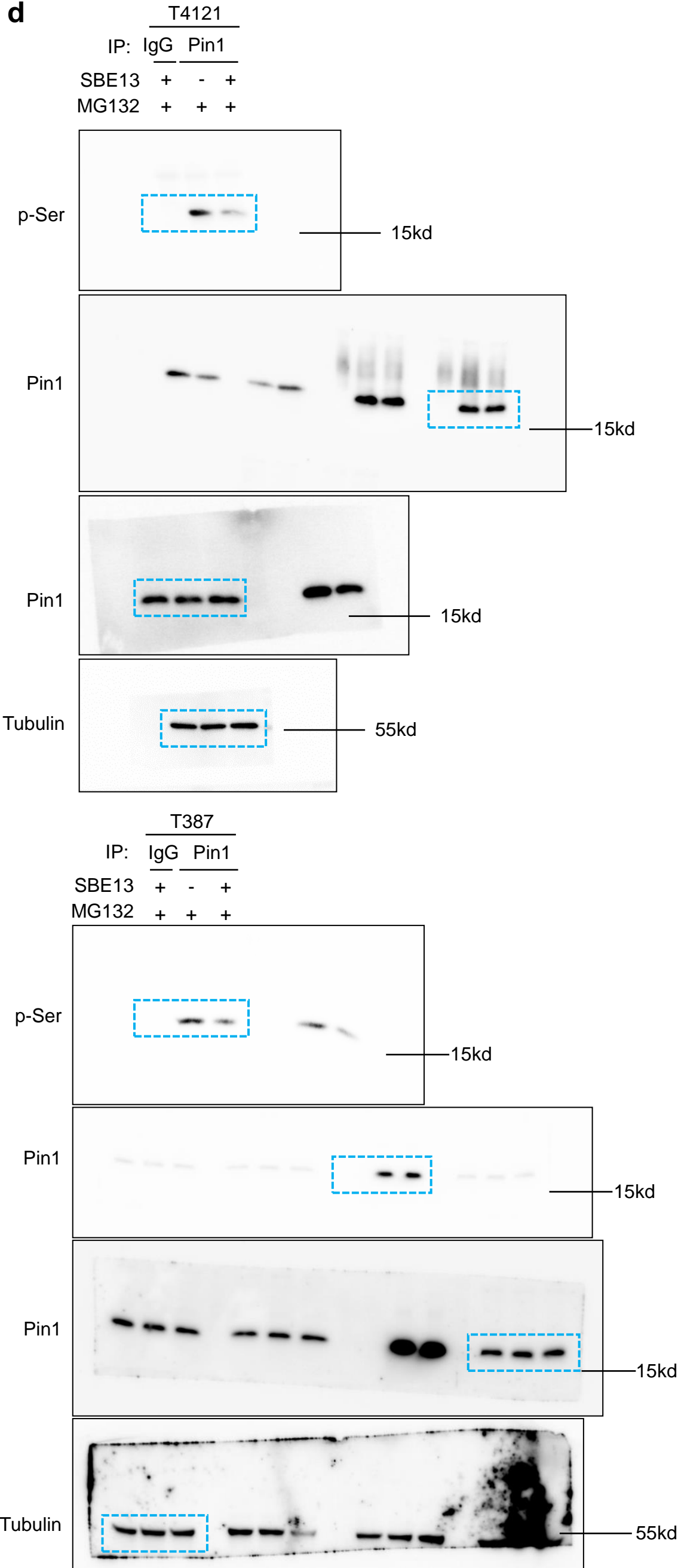

e

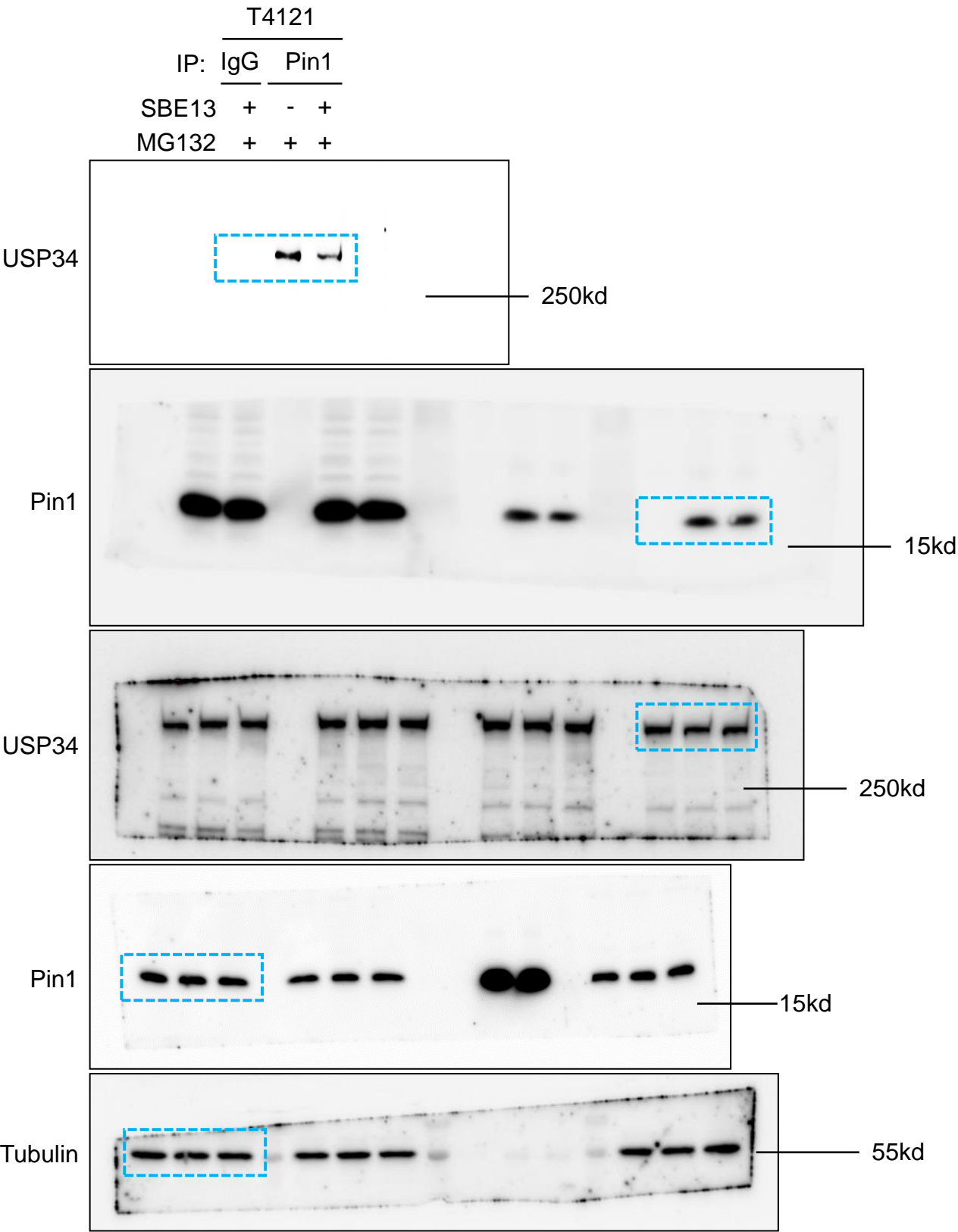

e

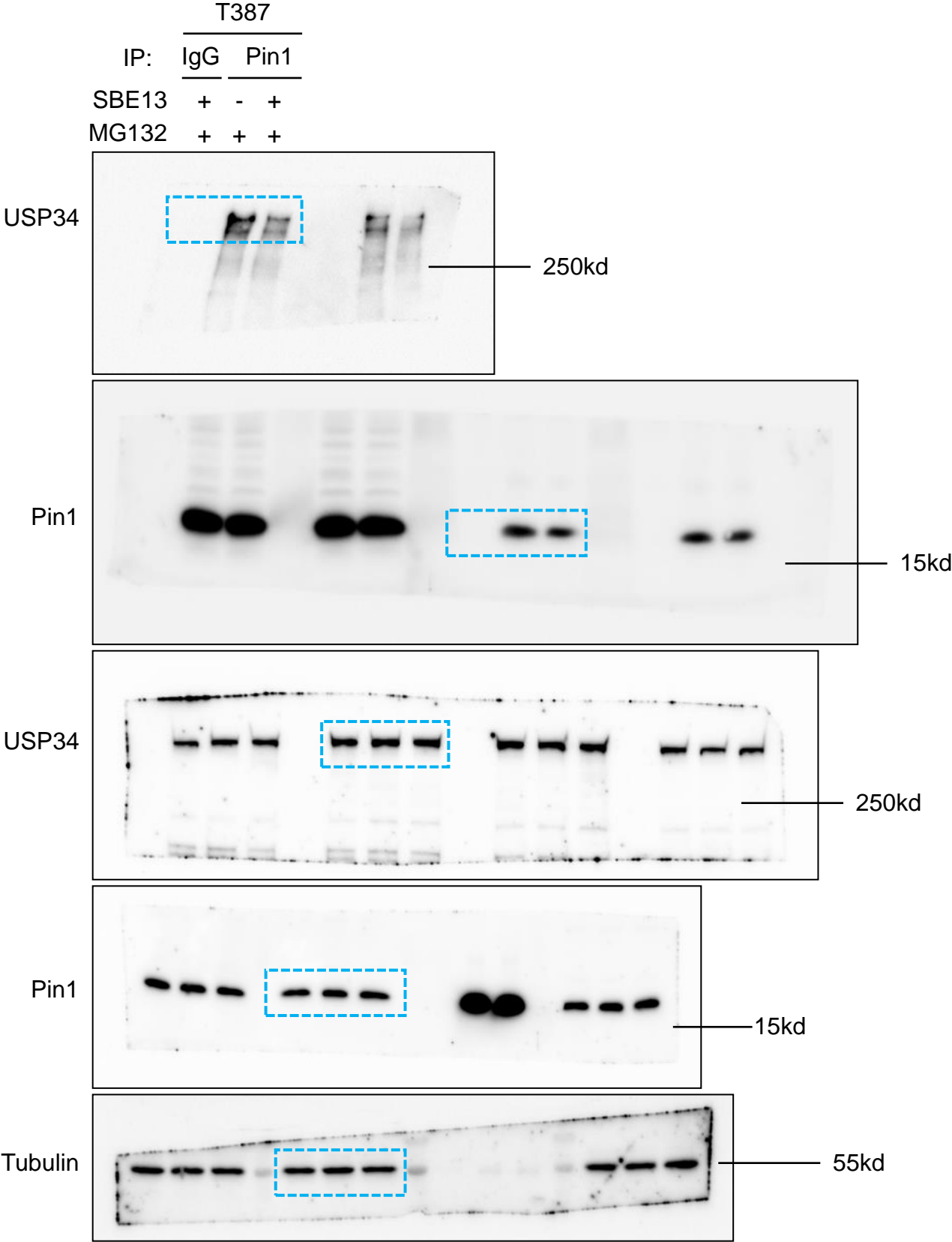

**f**

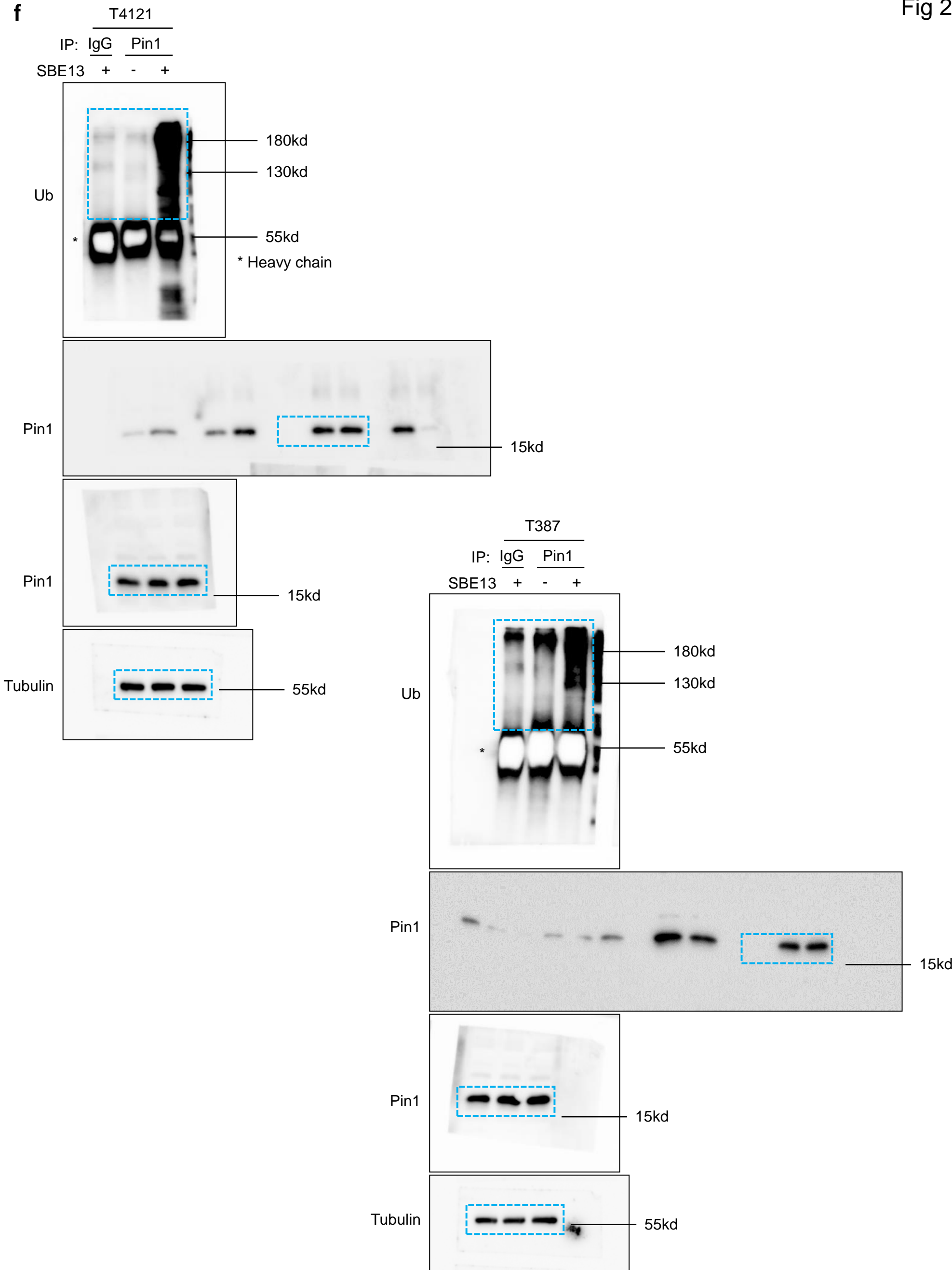

**g**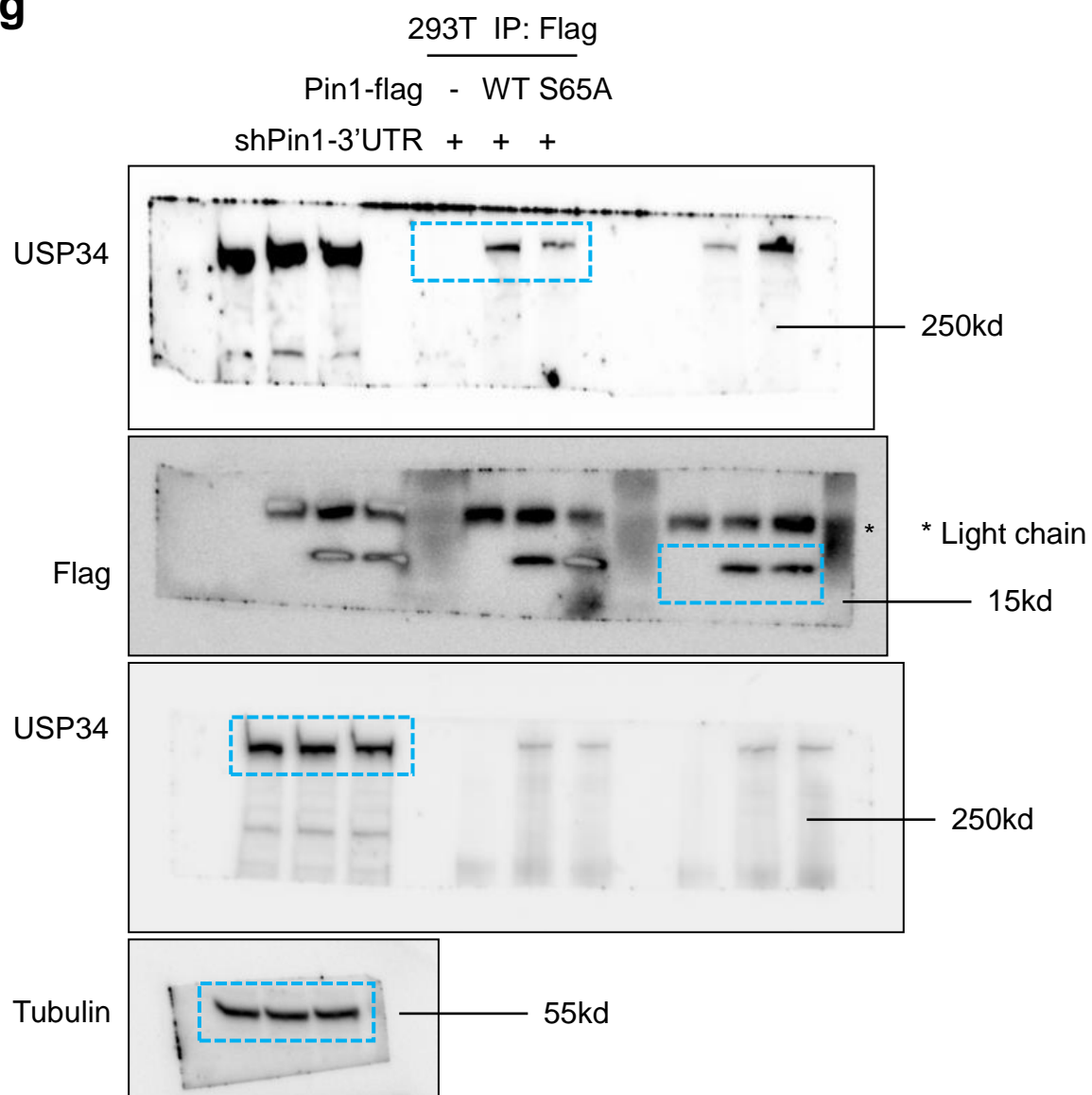**h**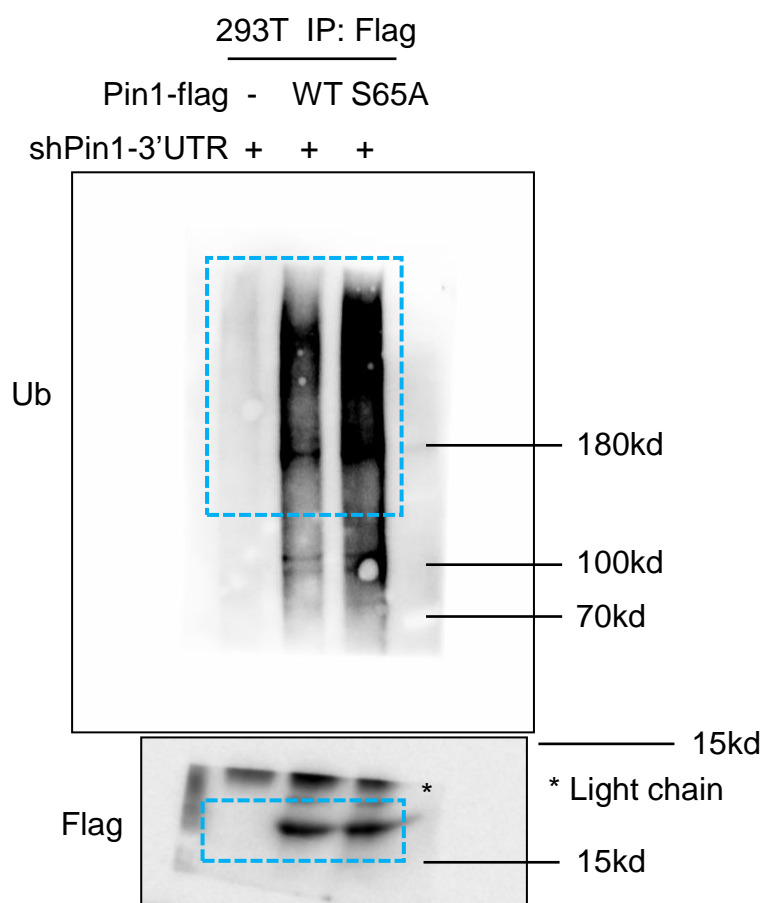

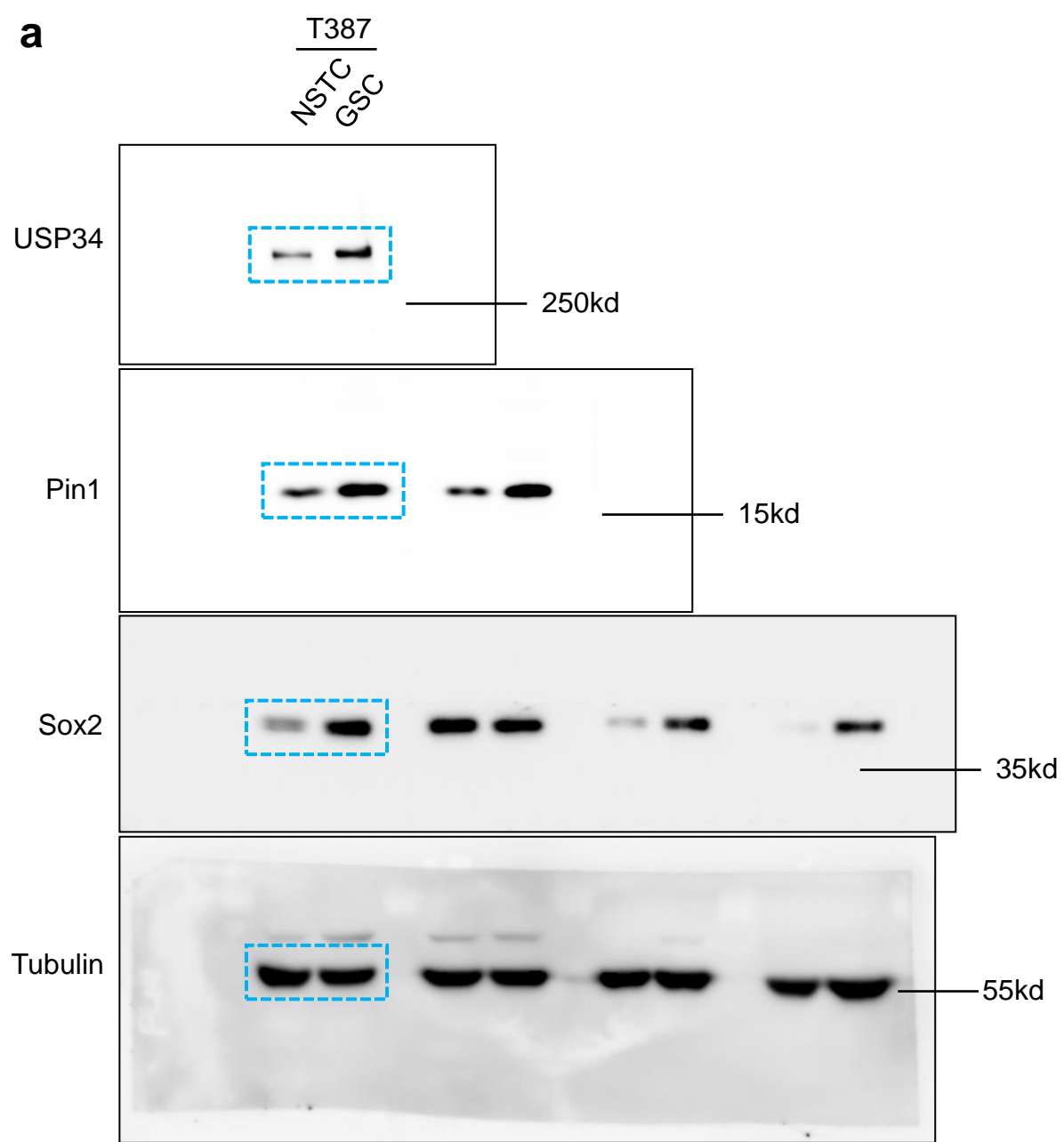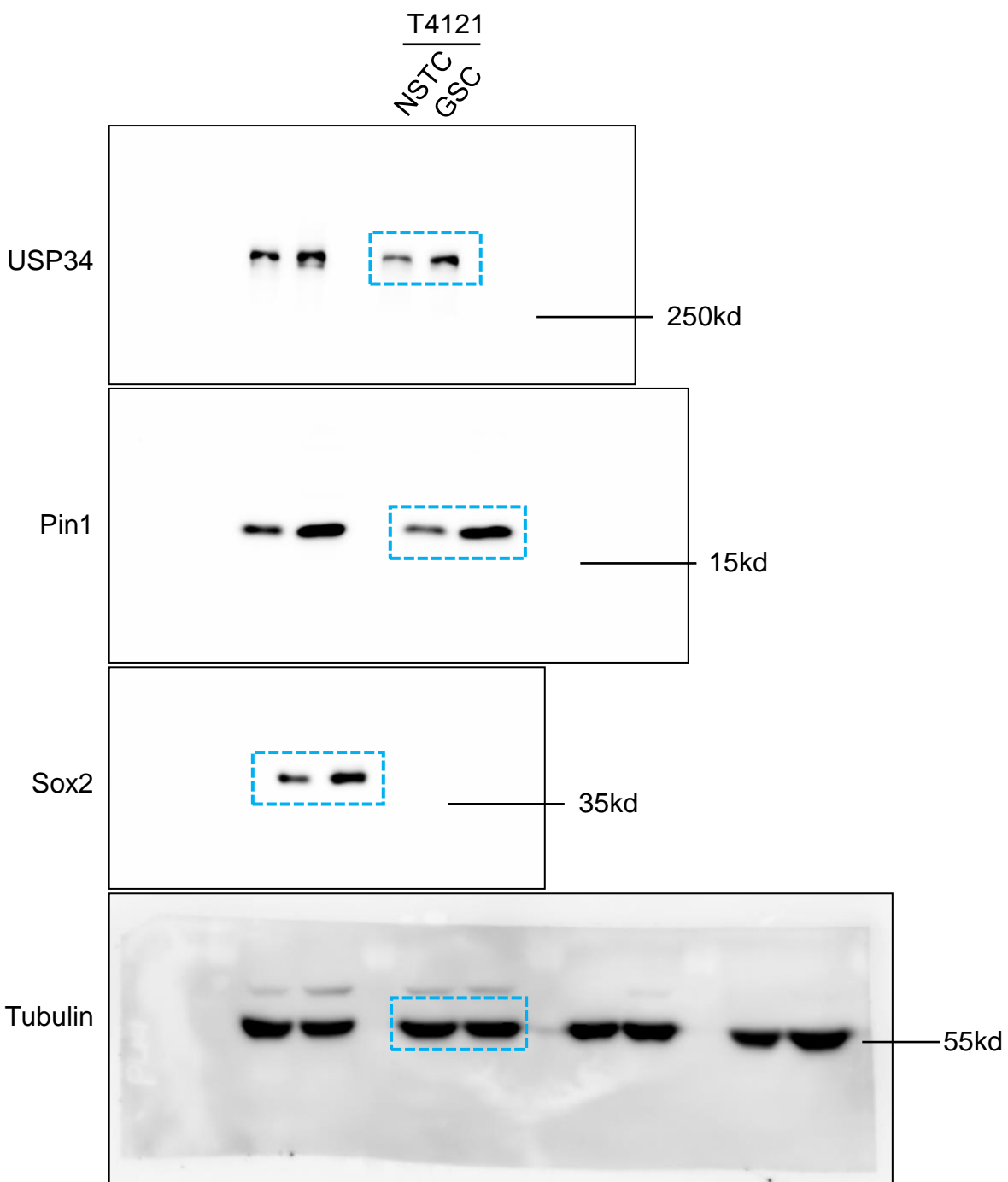

**a**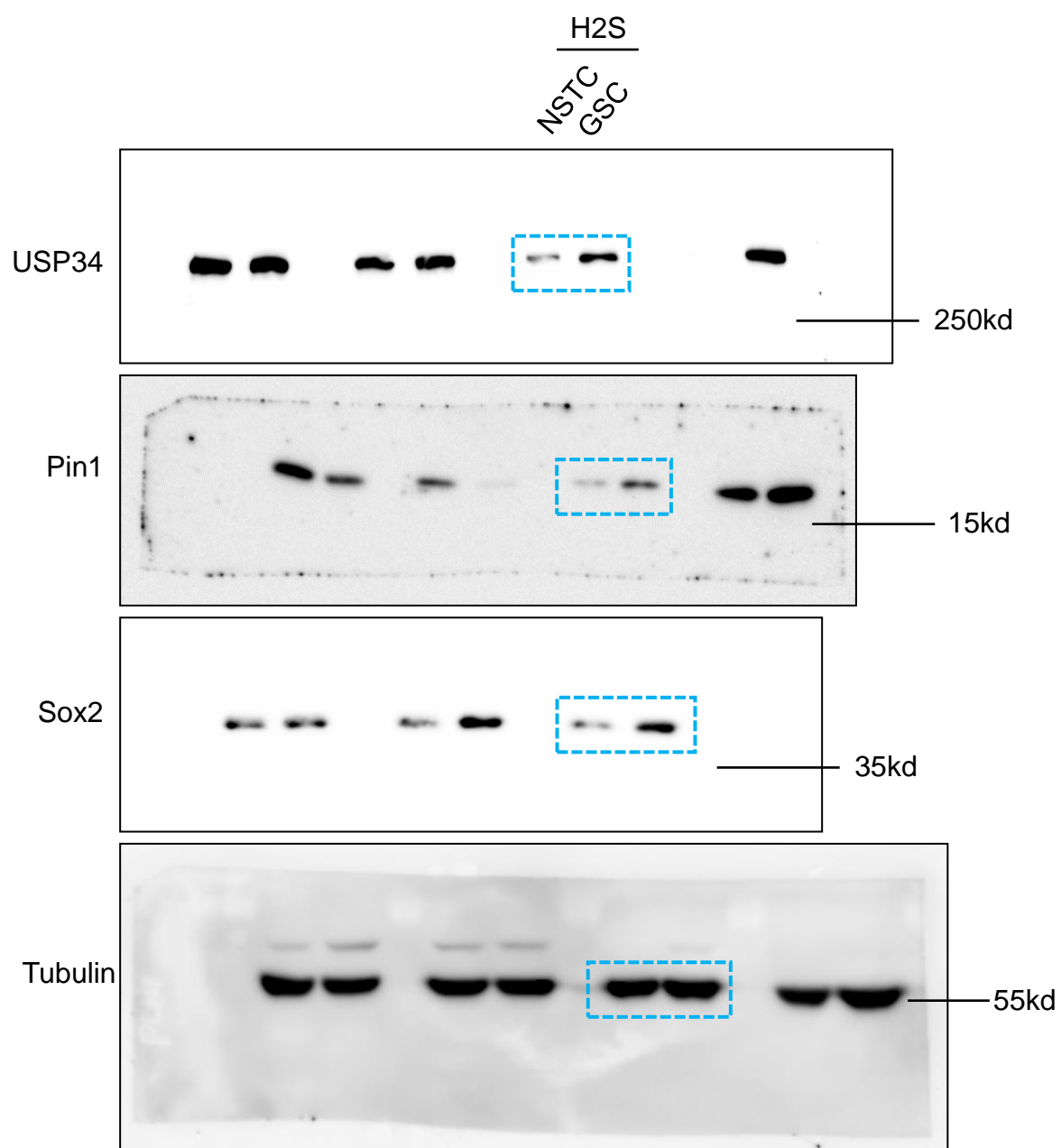**d**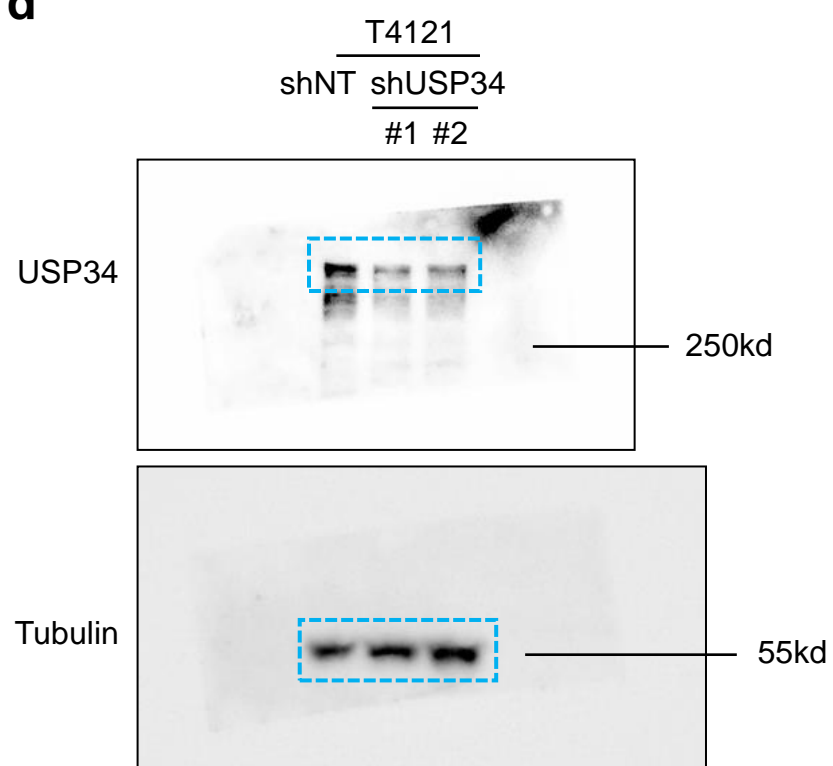

**a**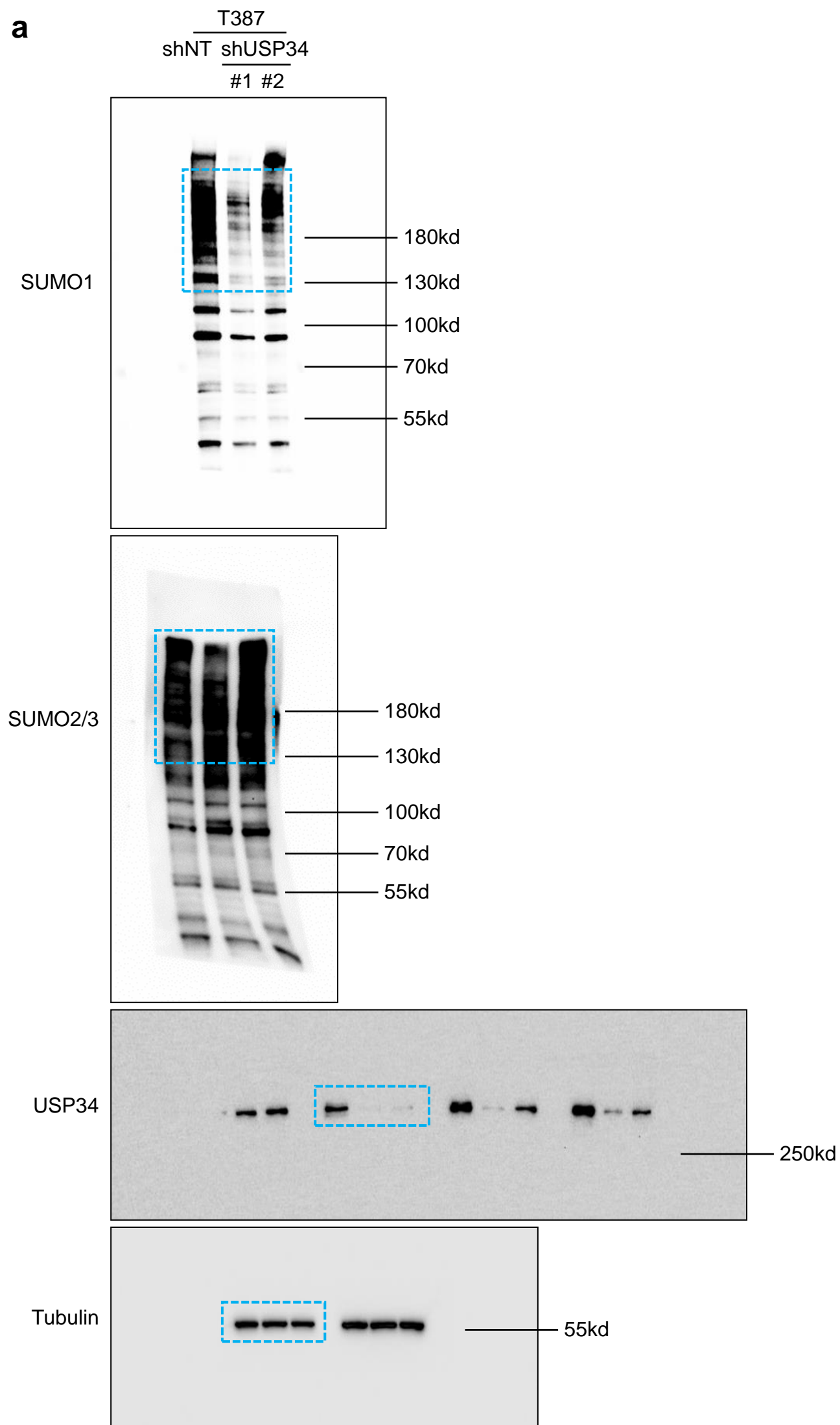

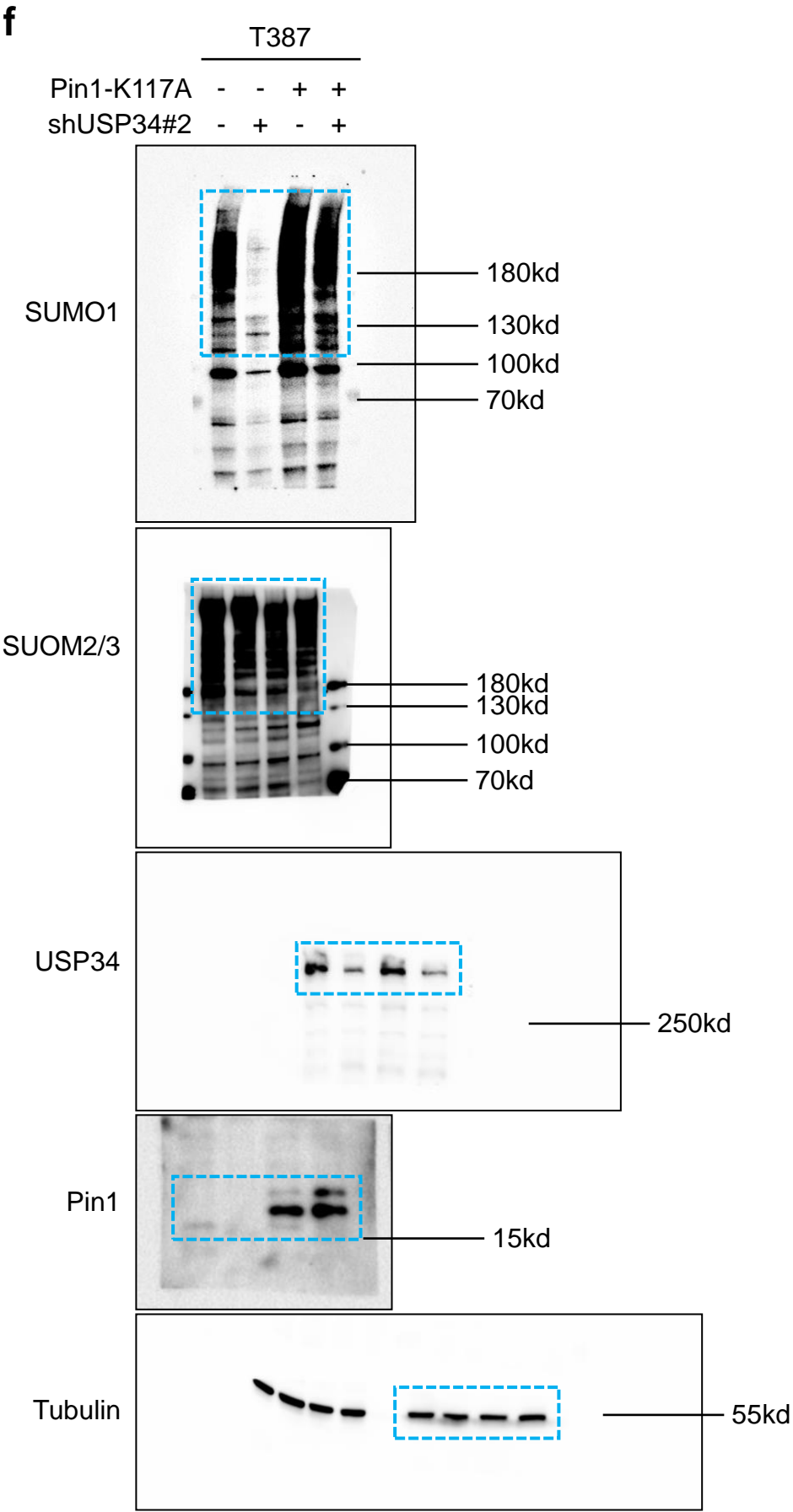

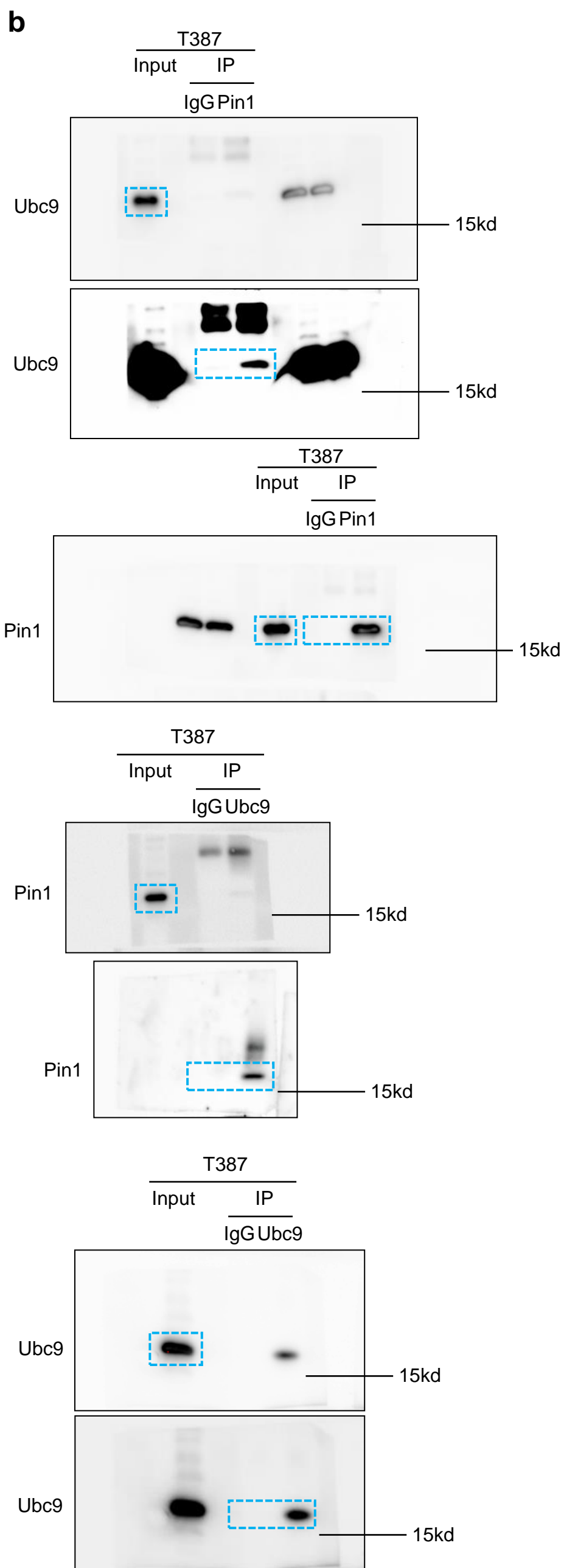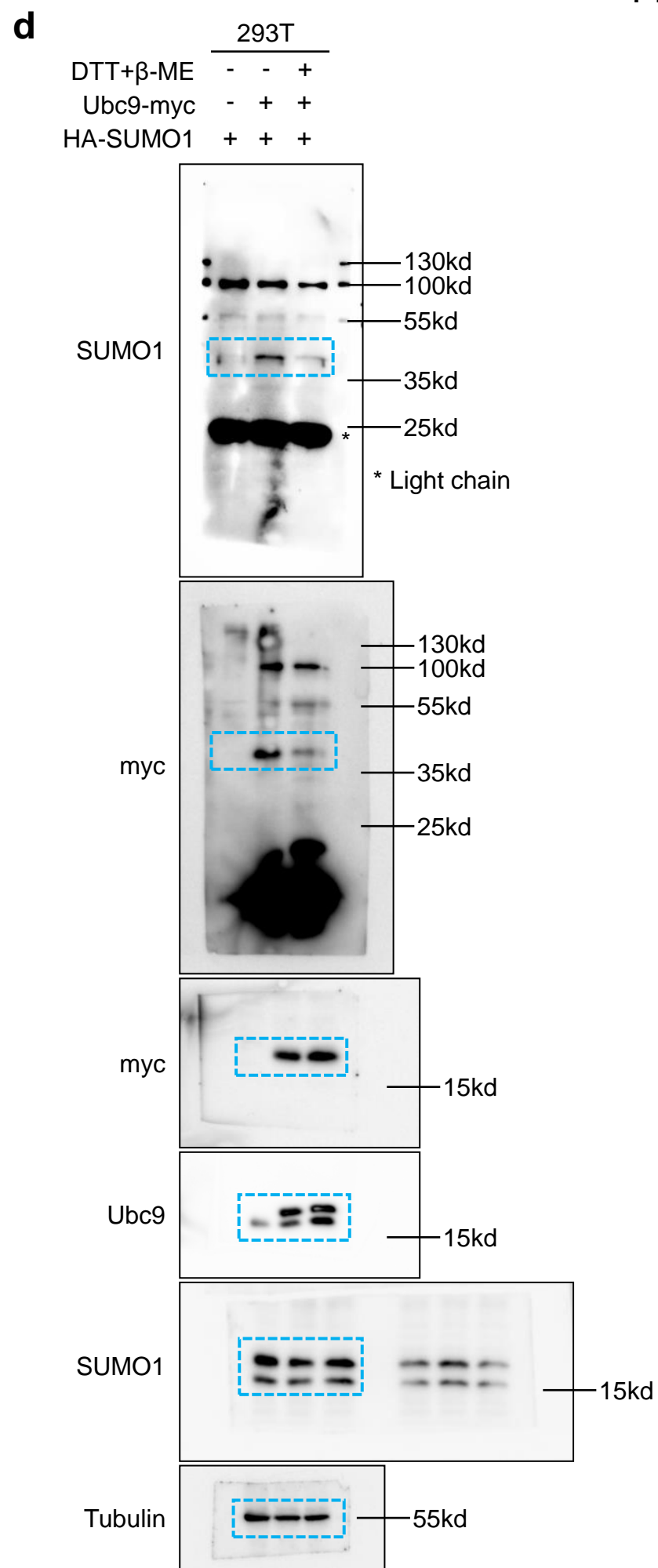

**e**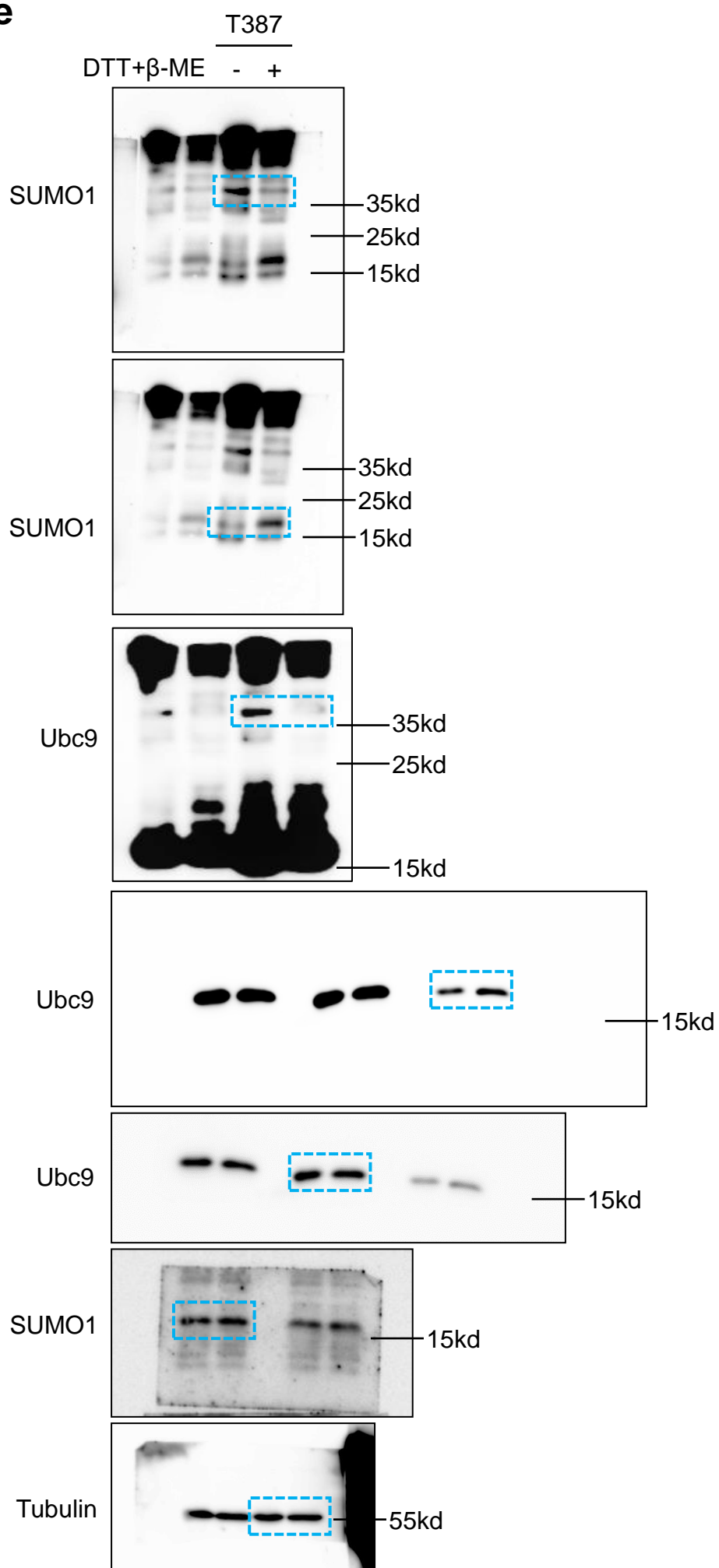**f**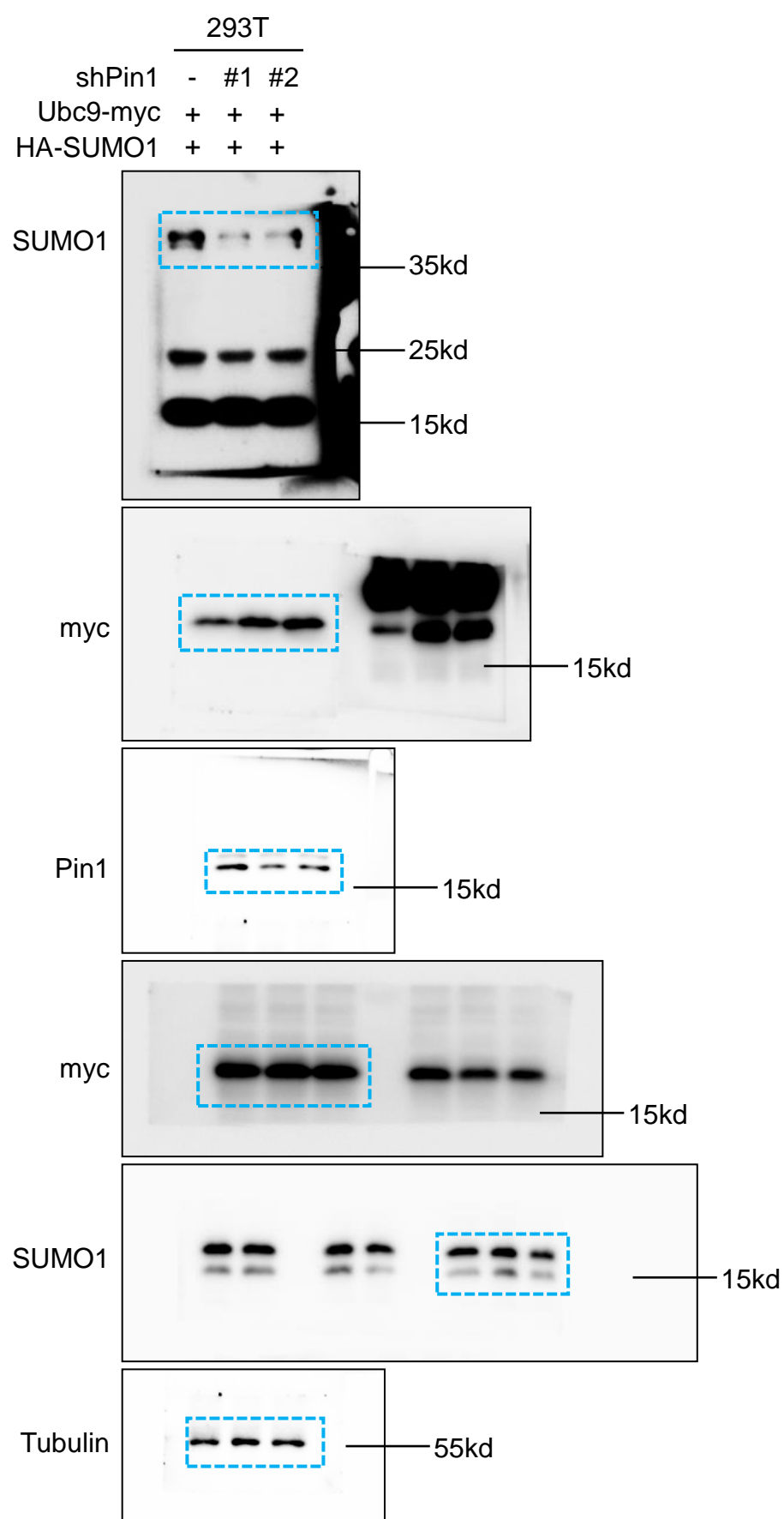

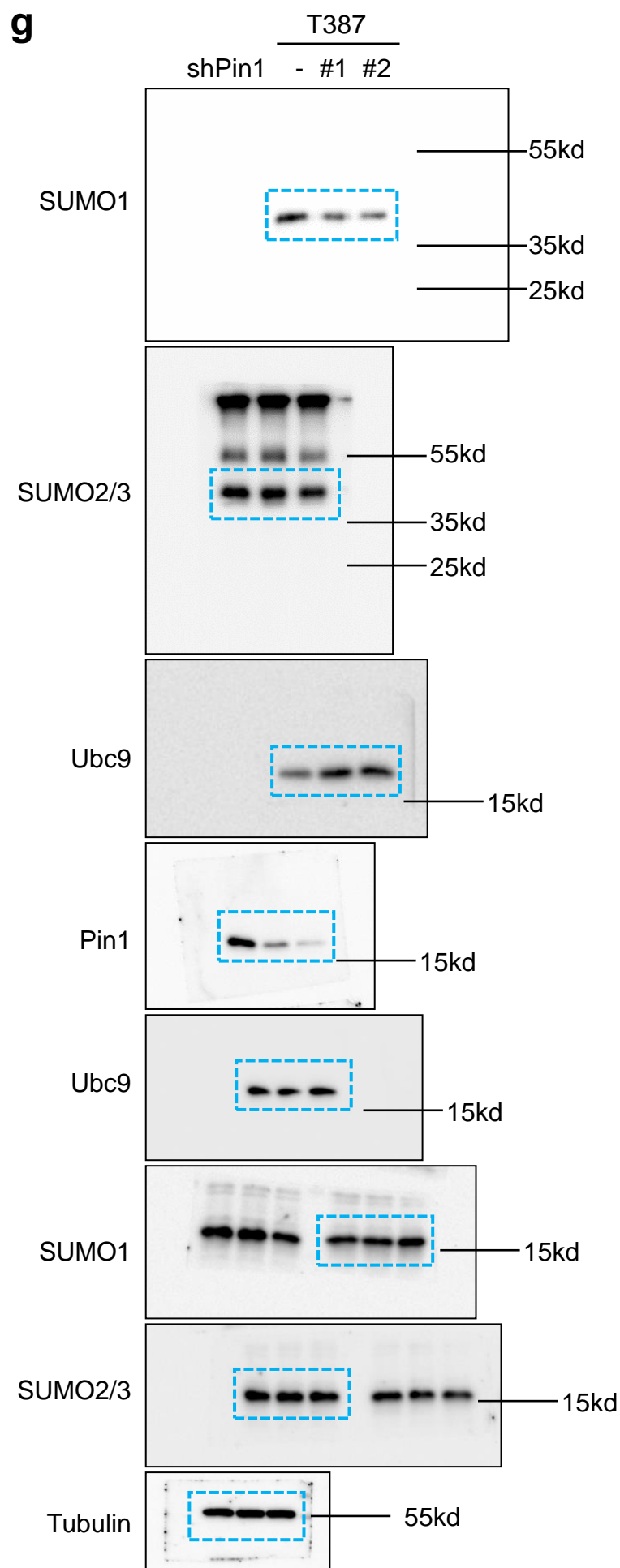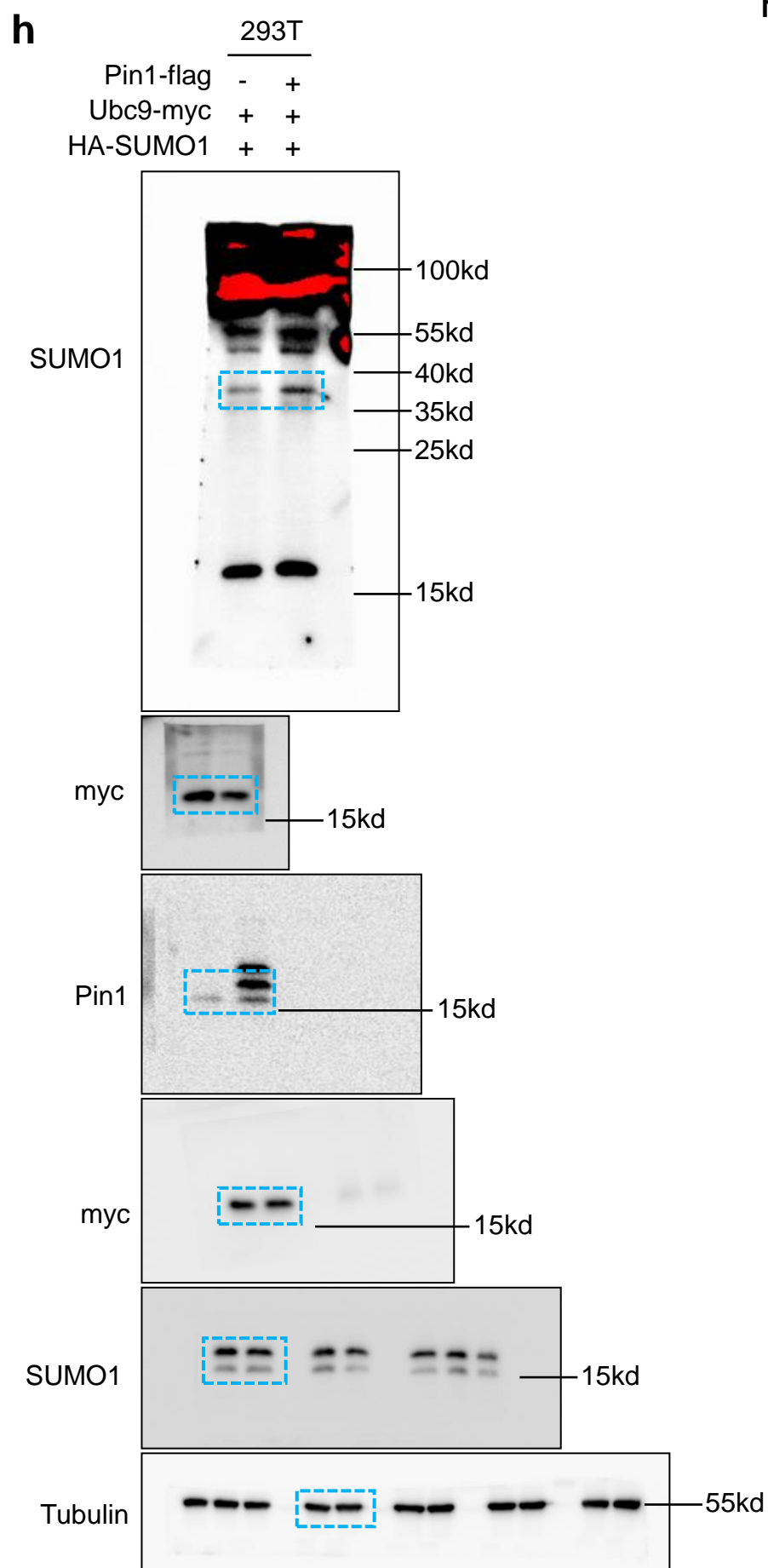

i

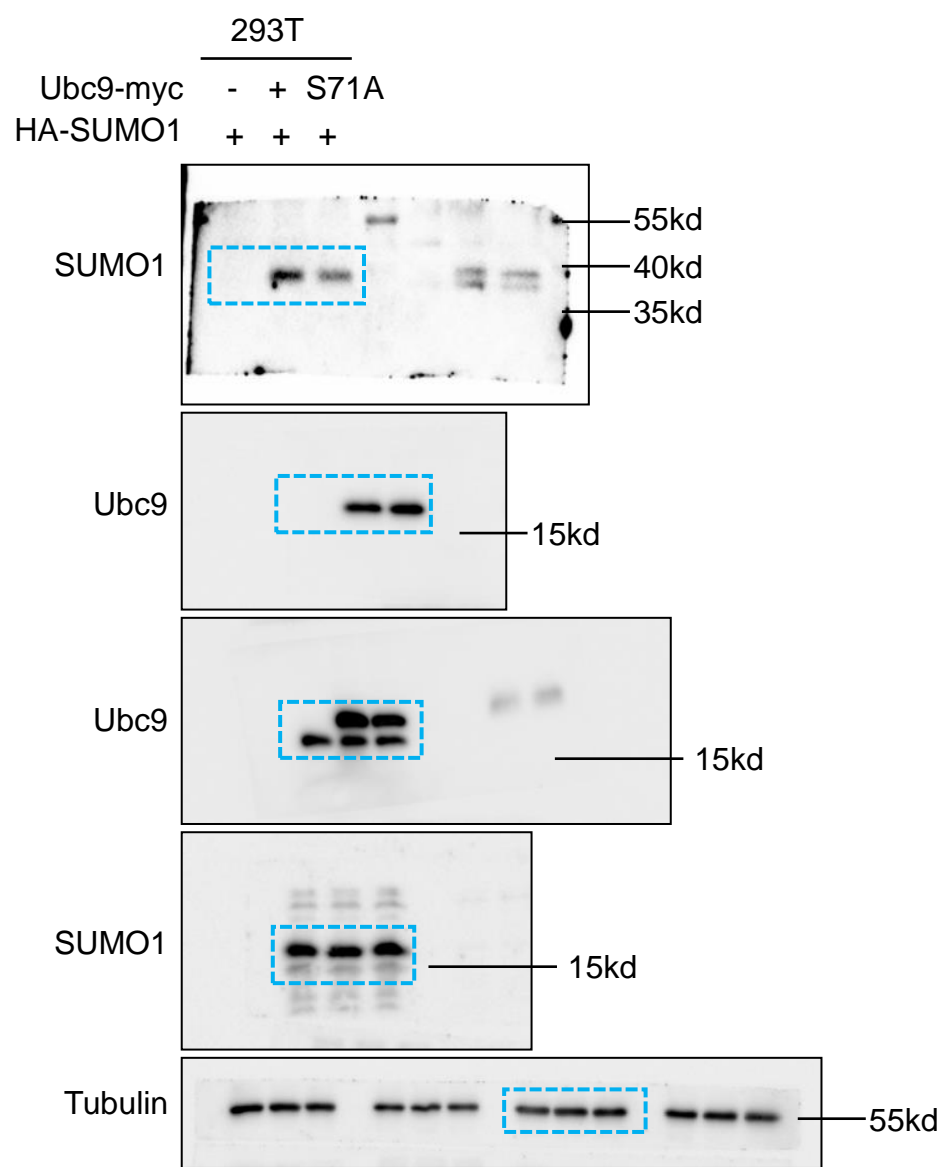

j

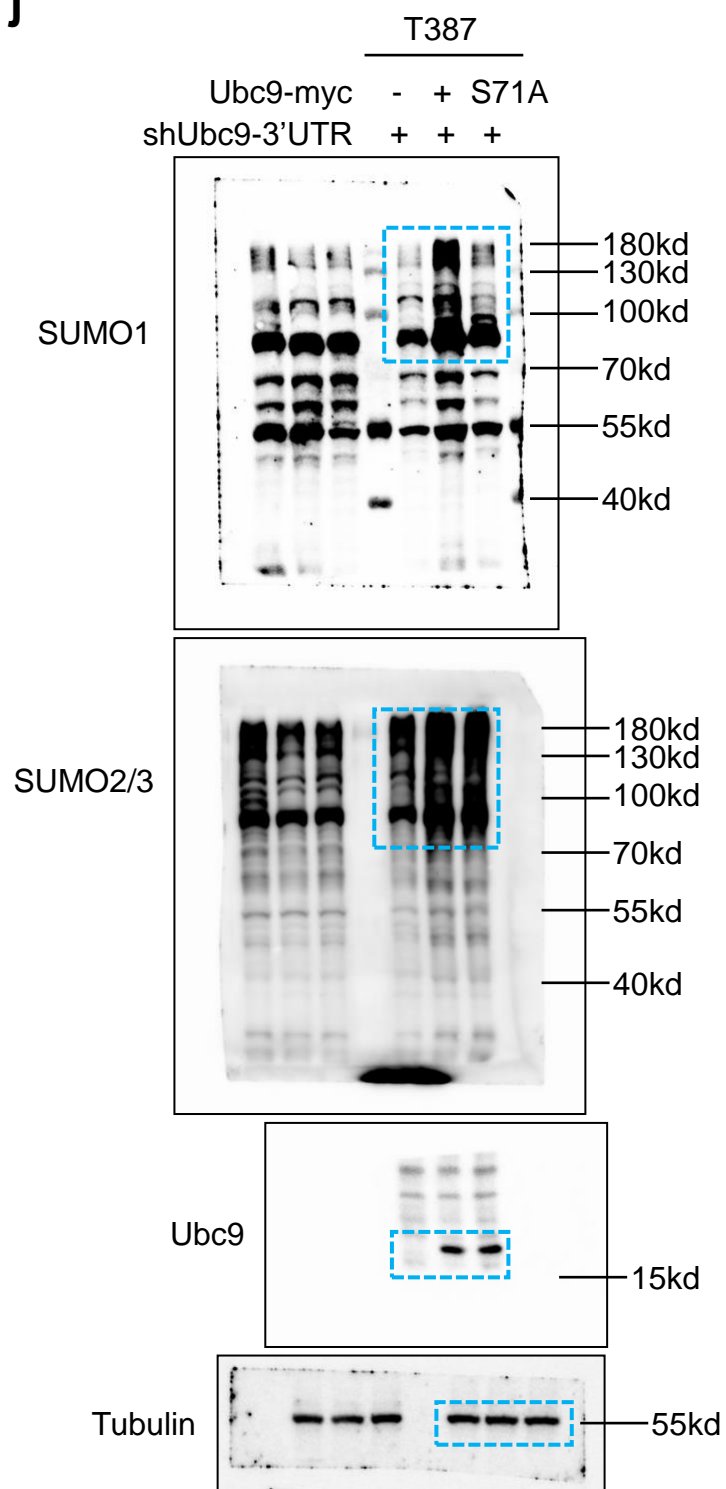

**a**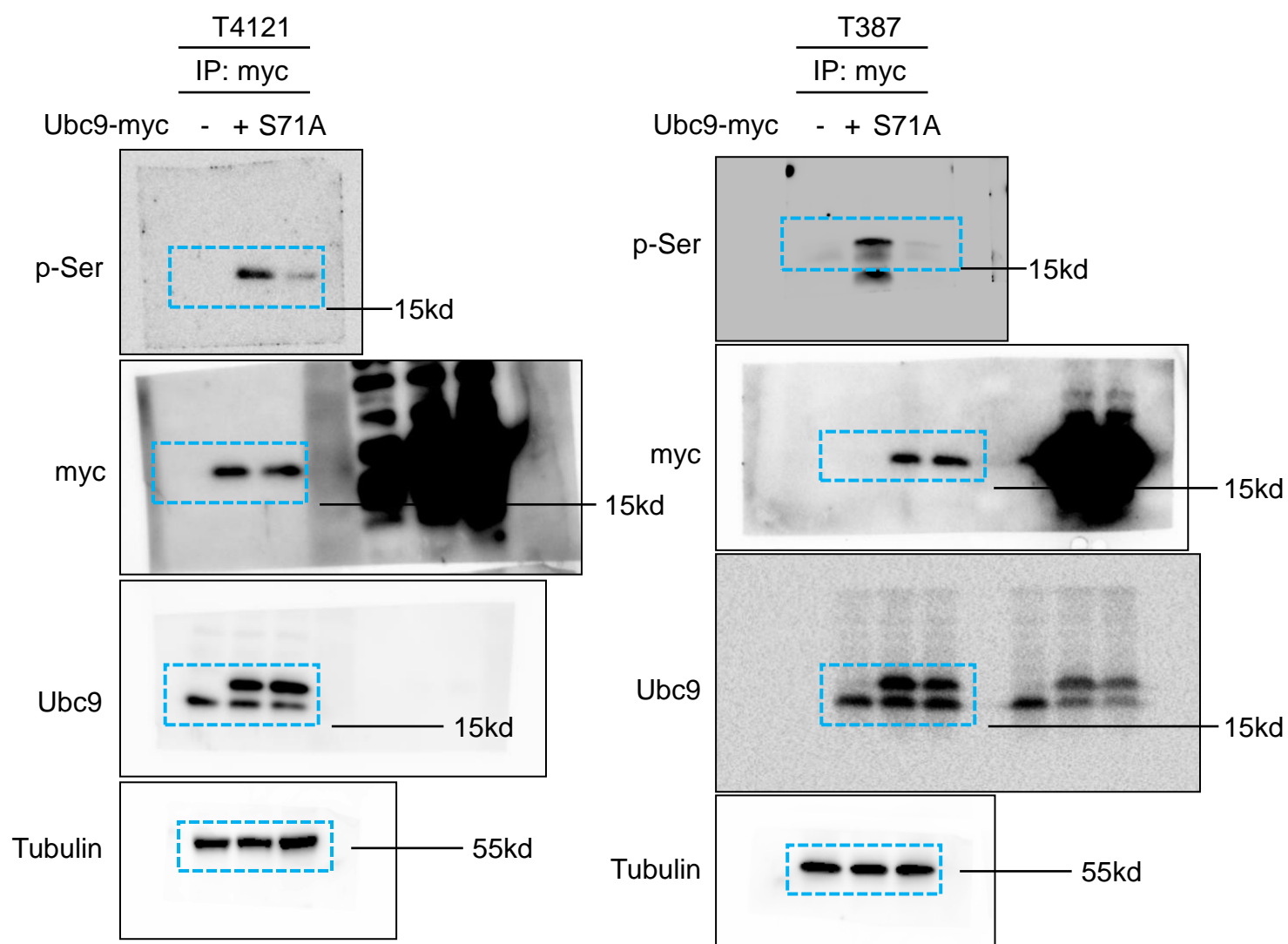**b**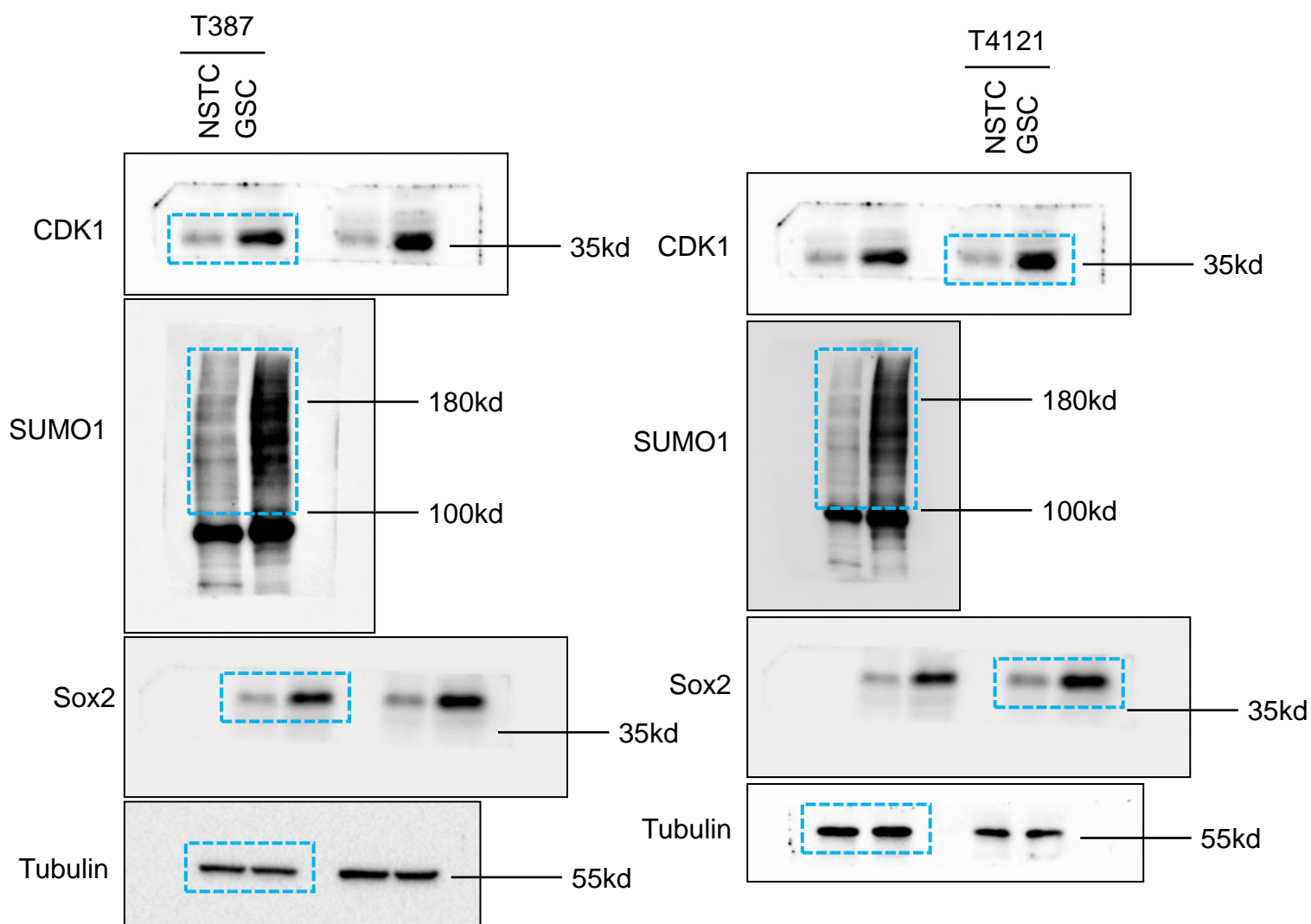

**C**

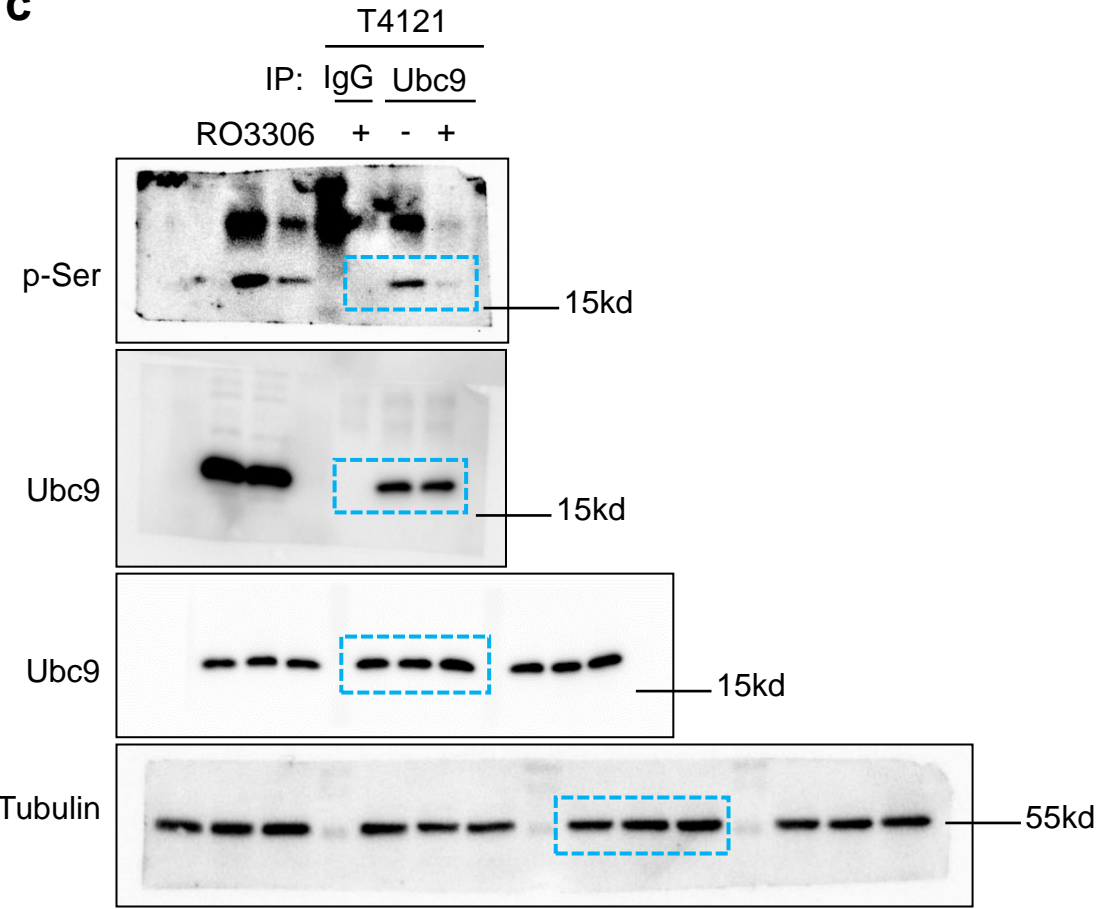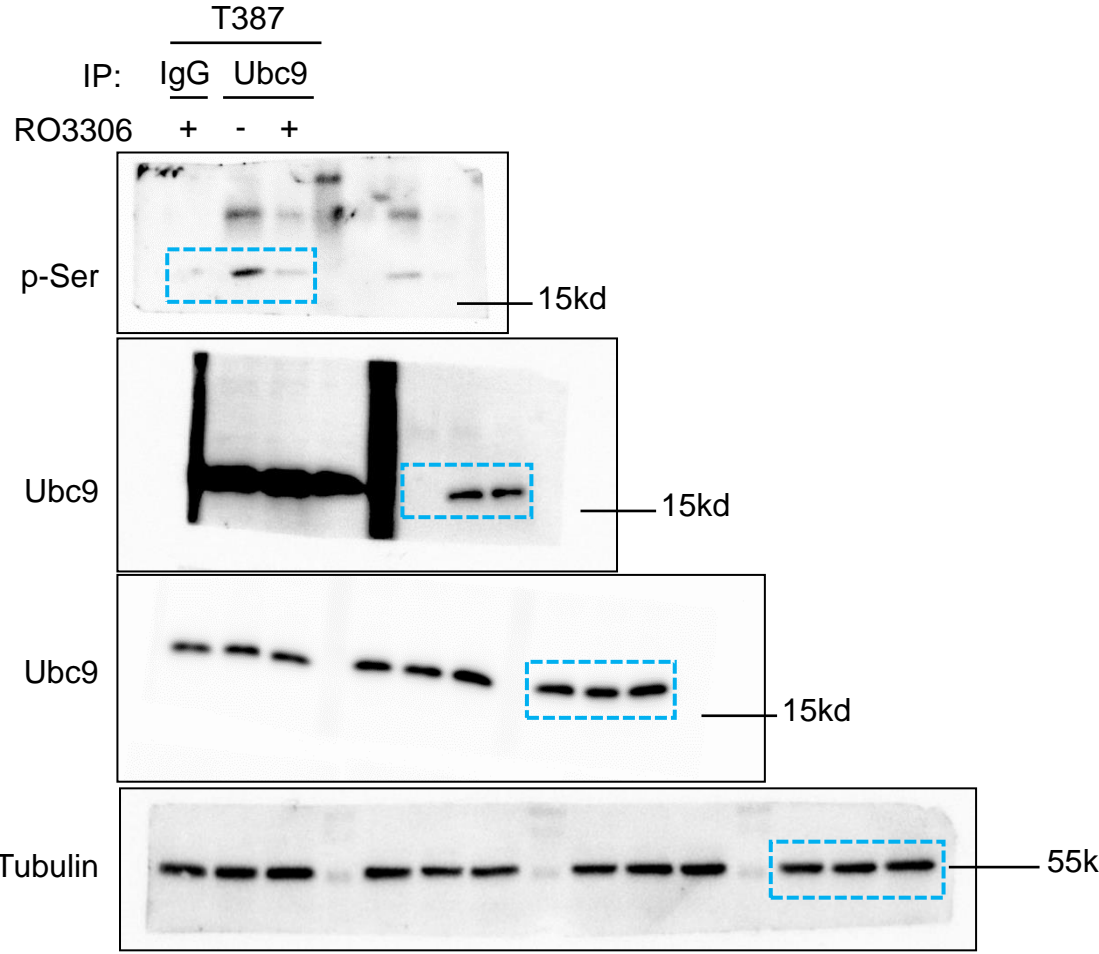

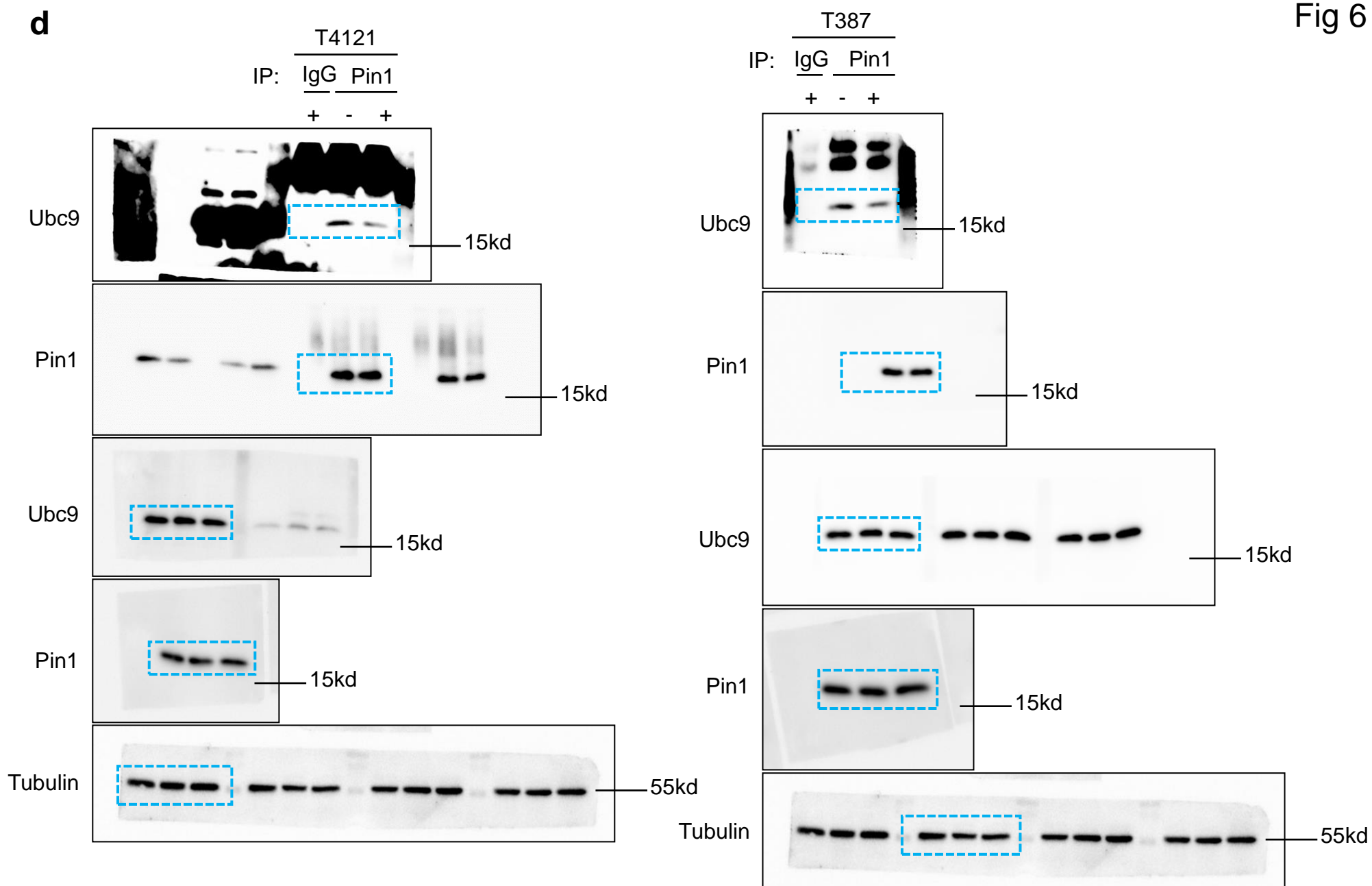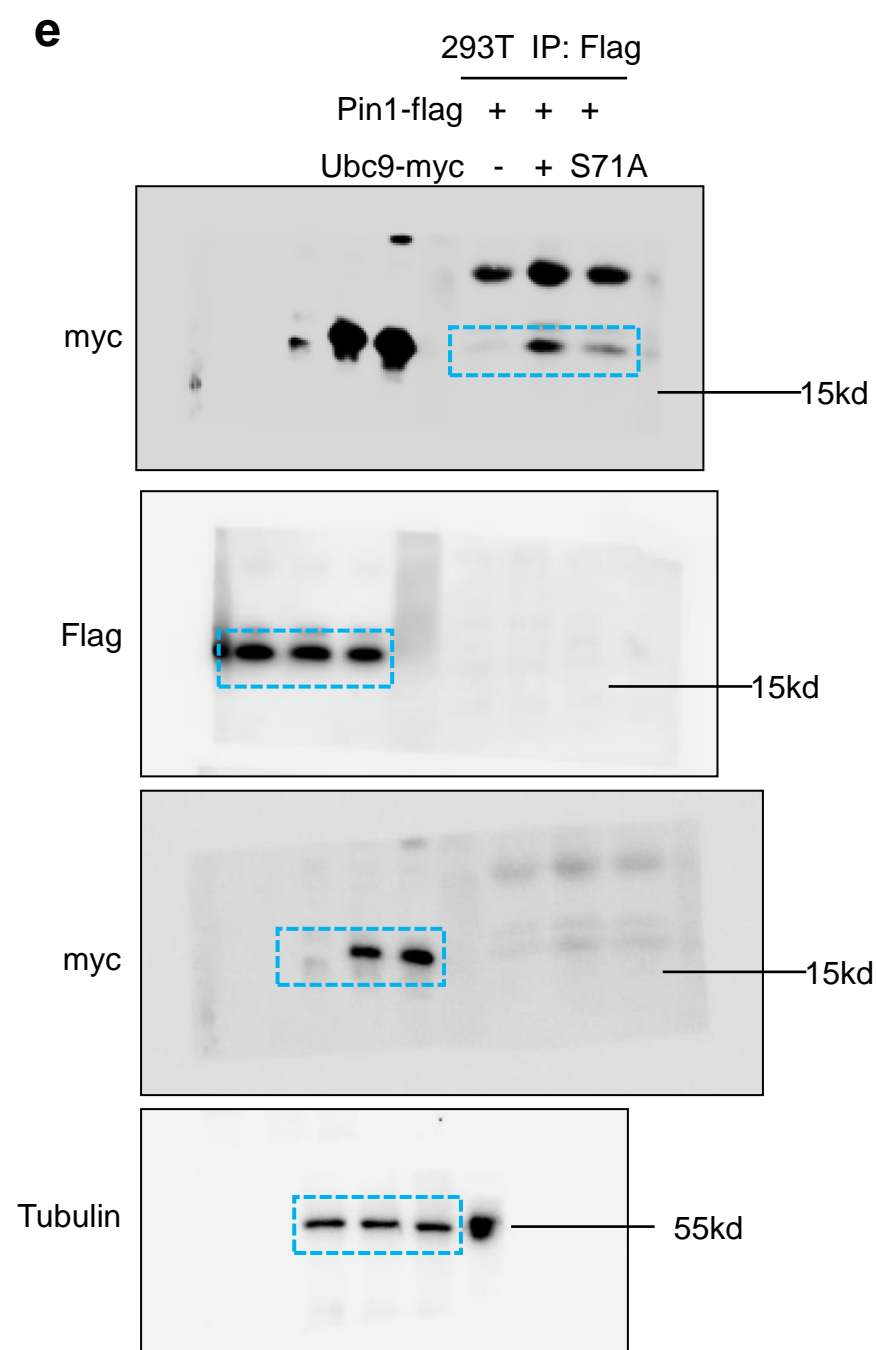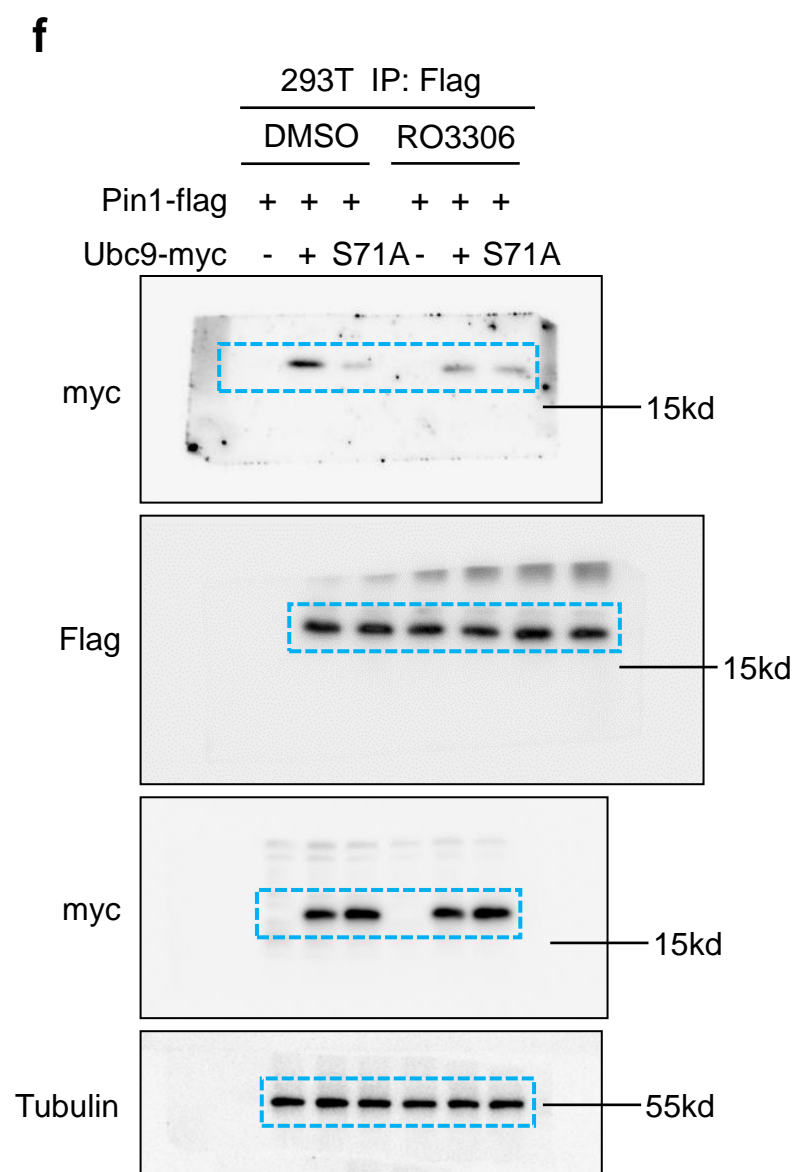

**g**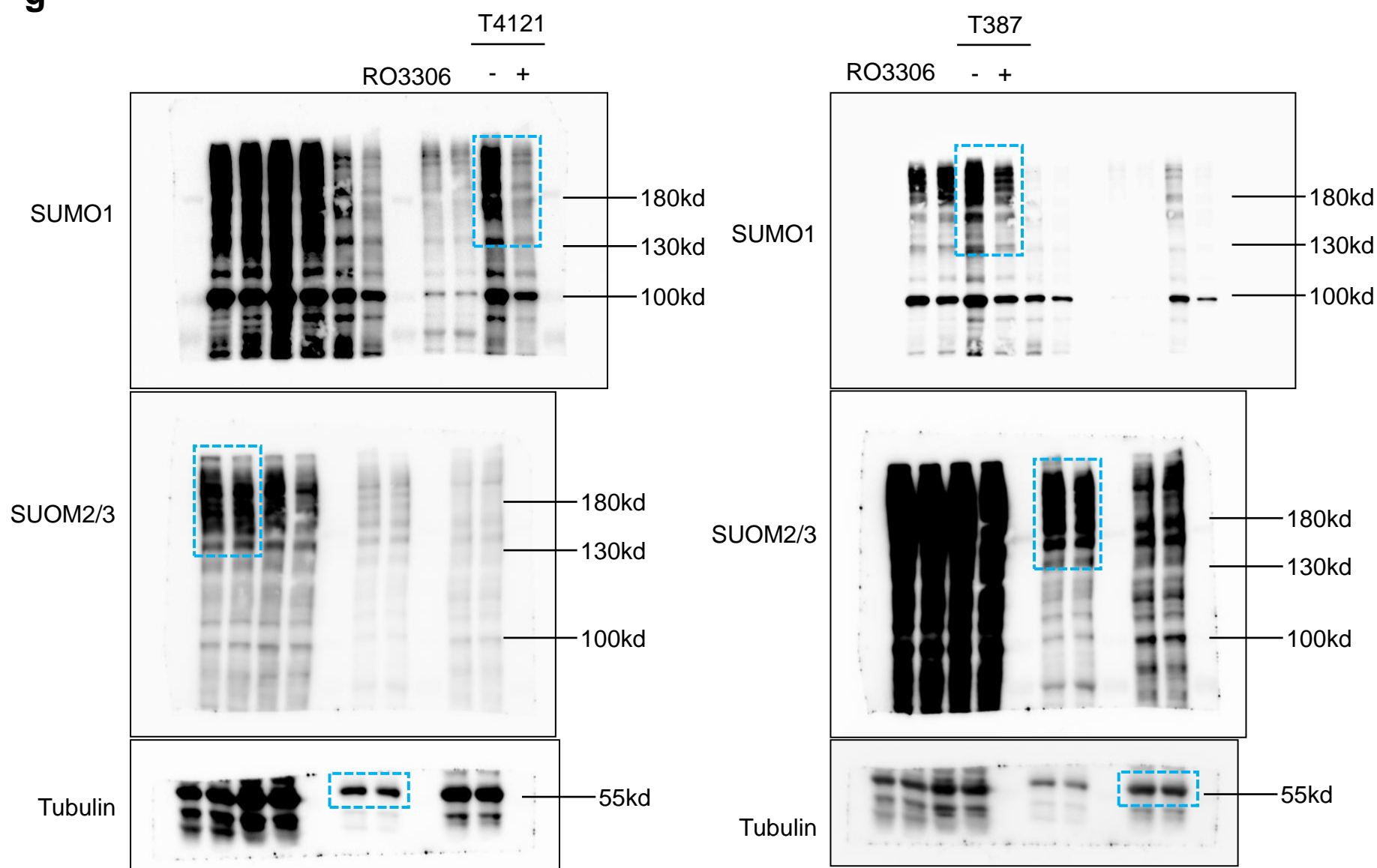

**b**

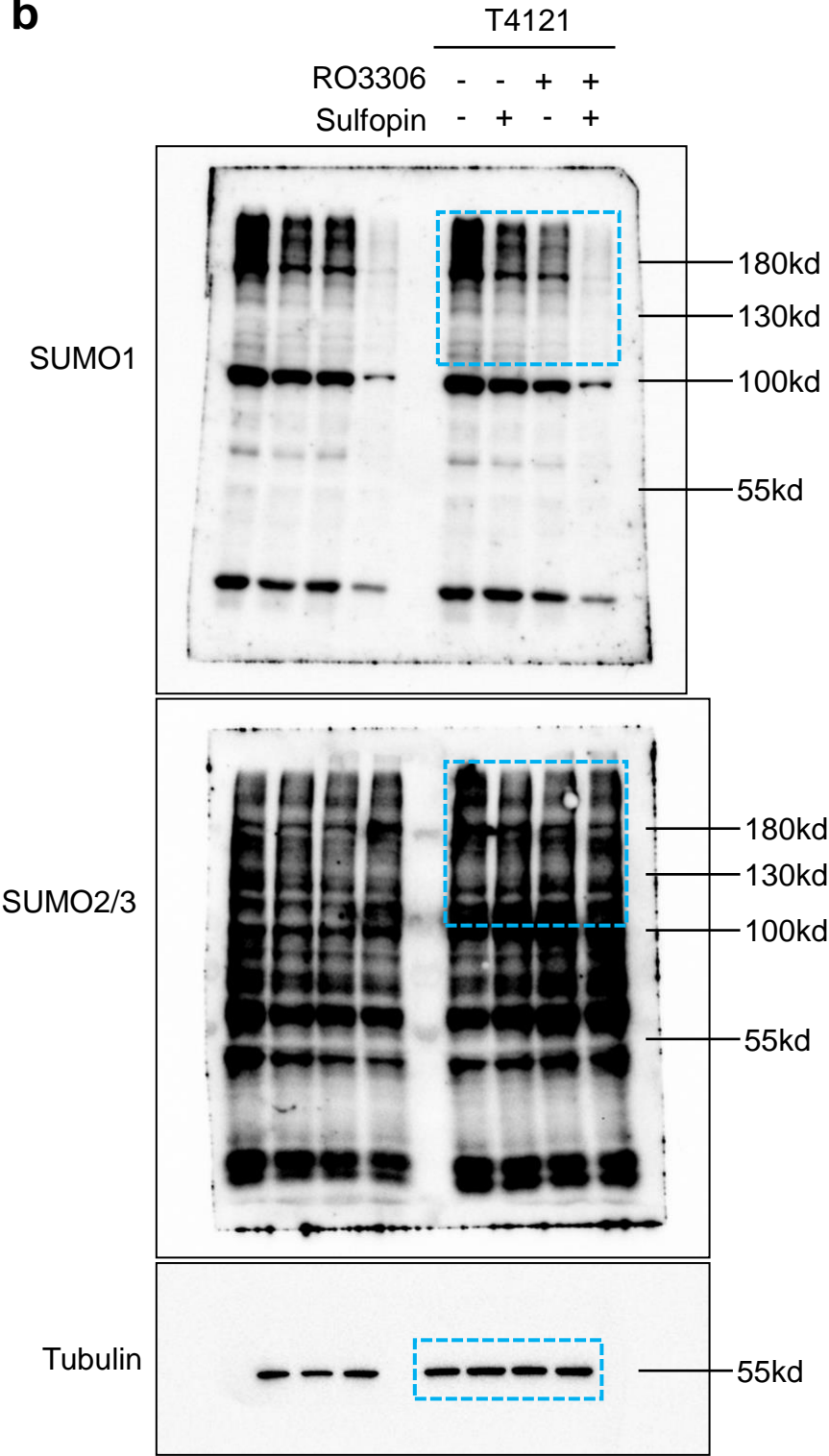

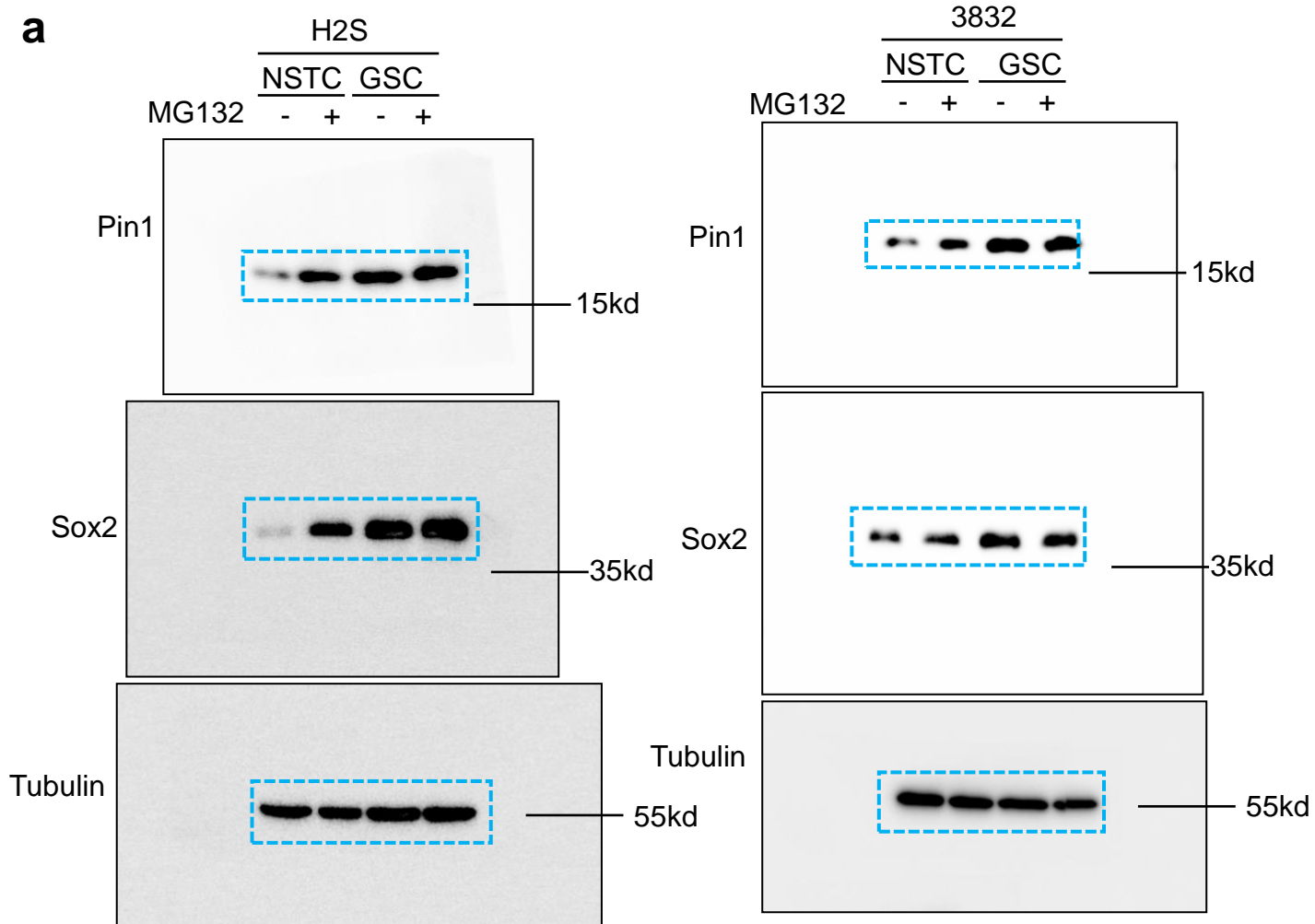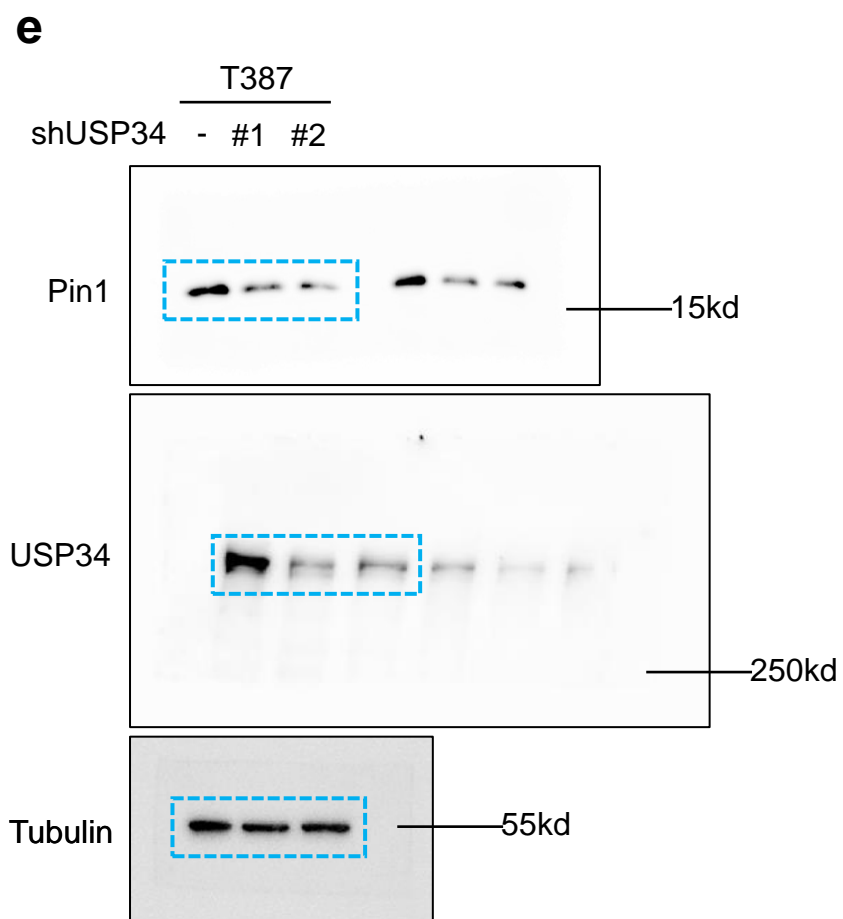

**f**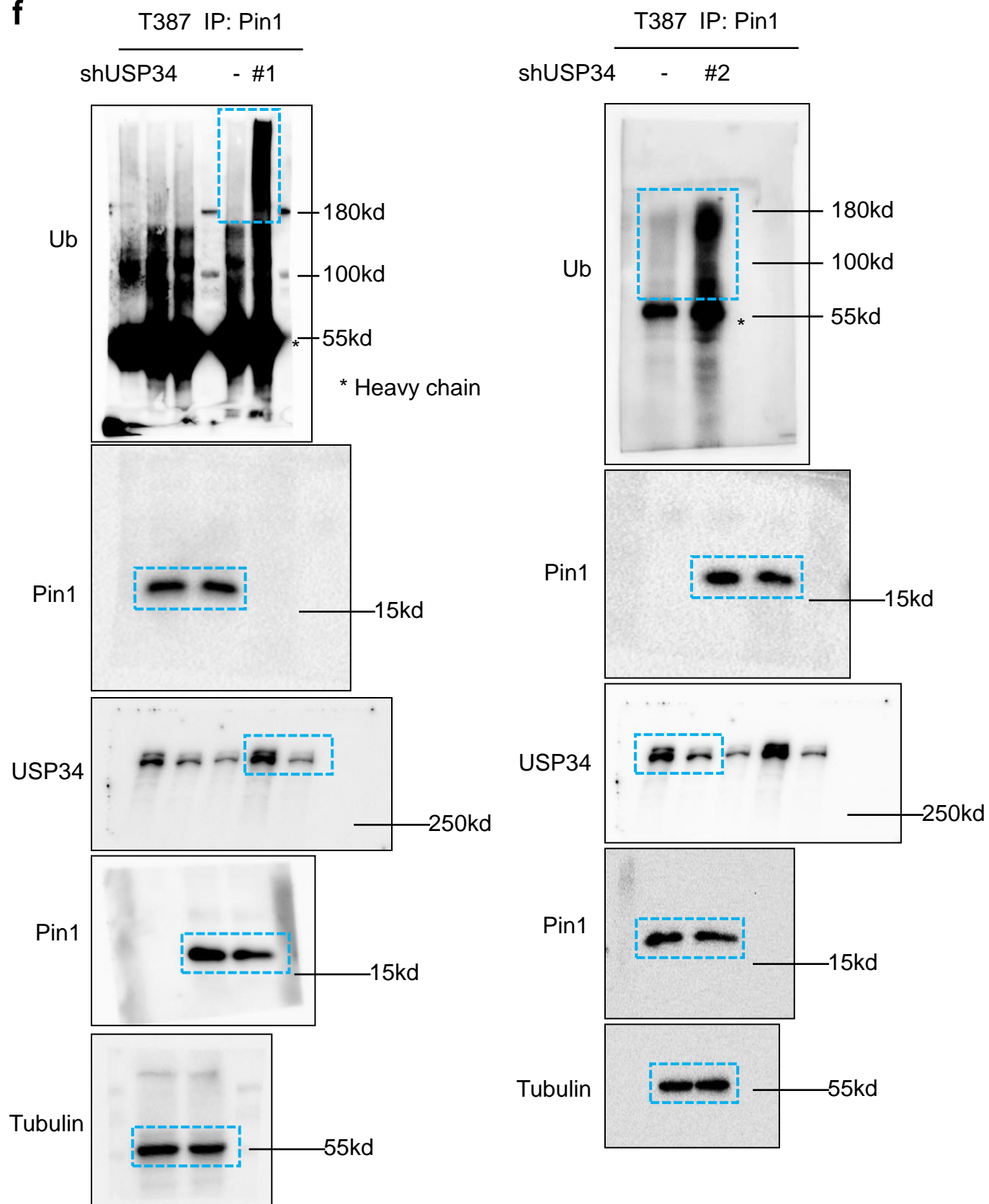**g**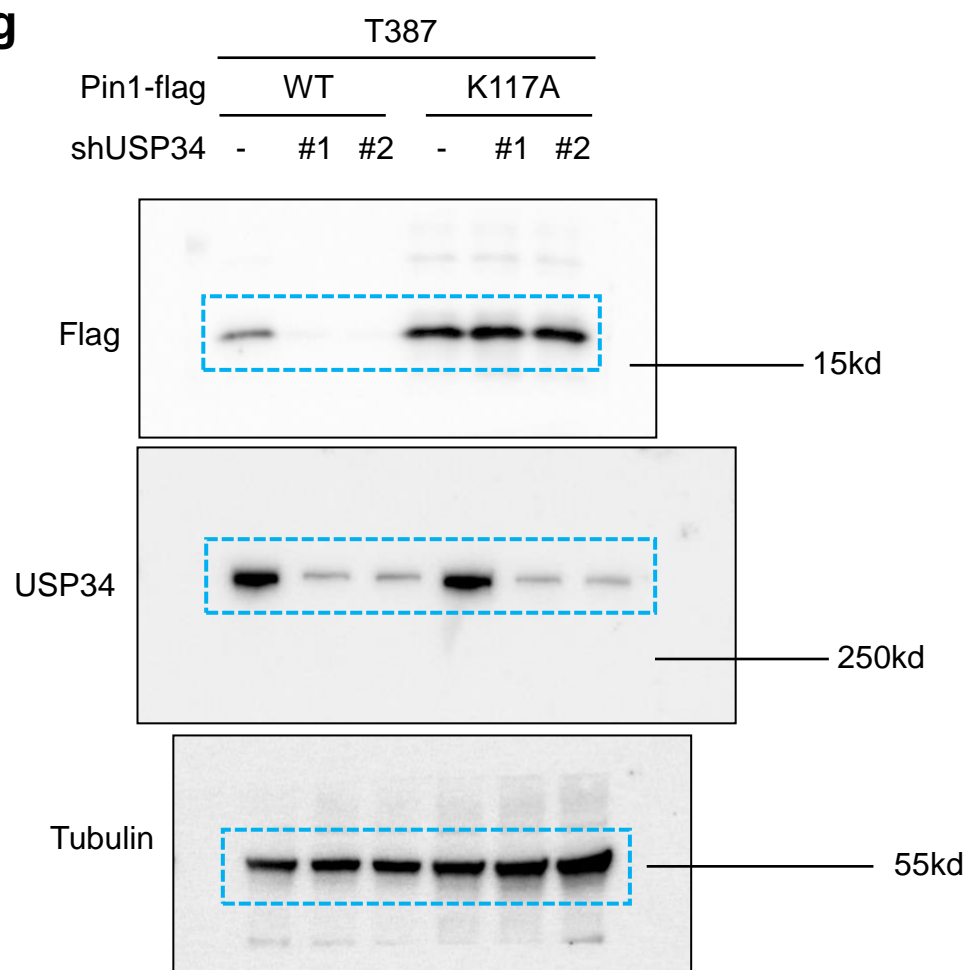

**d**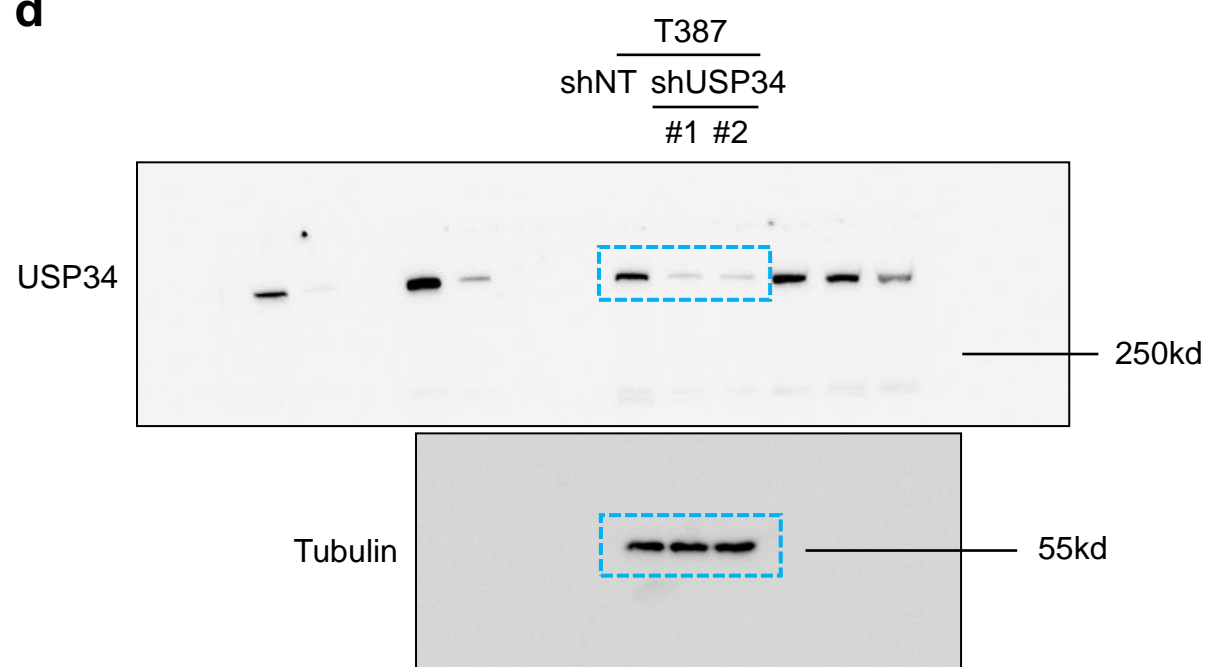

**a**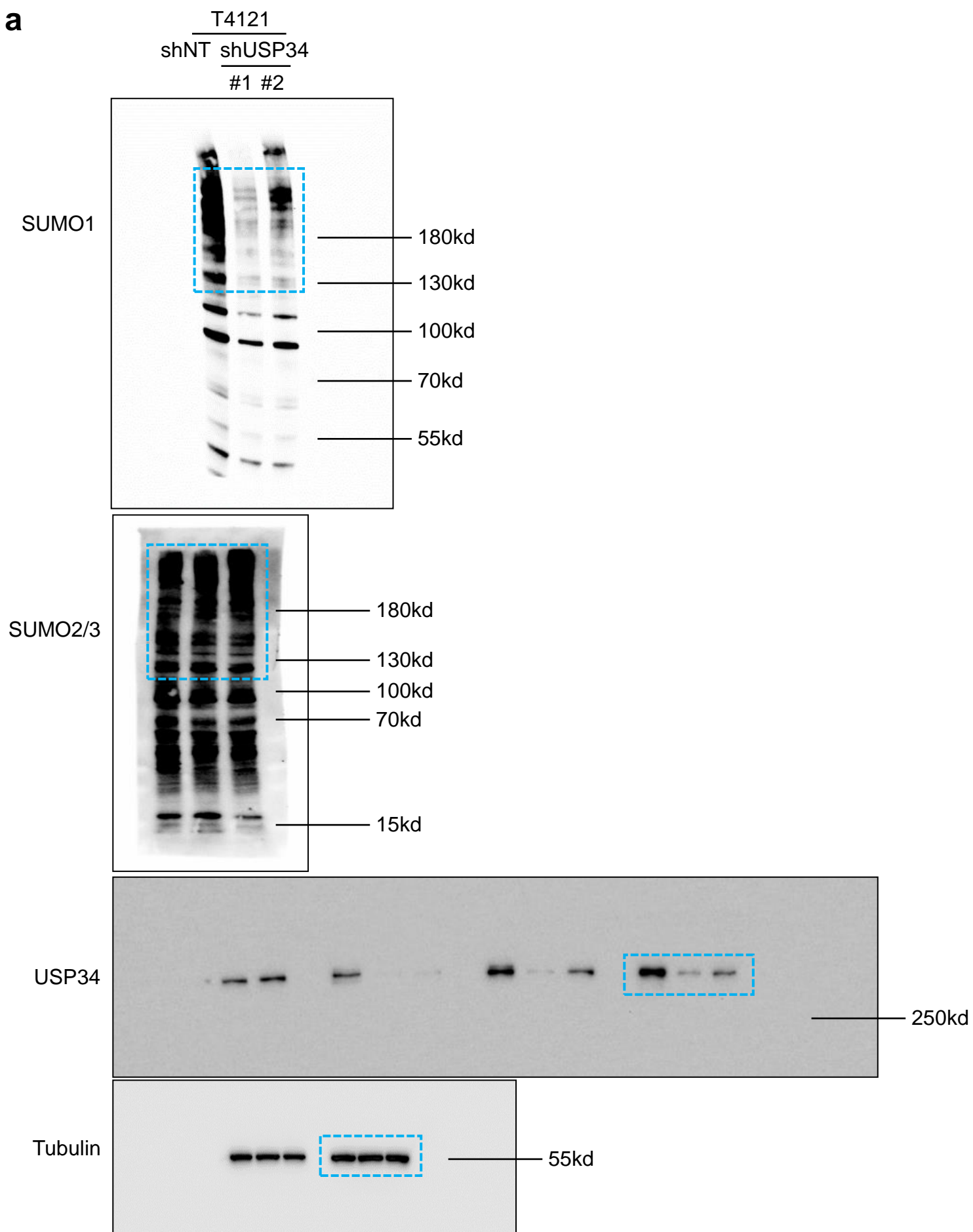

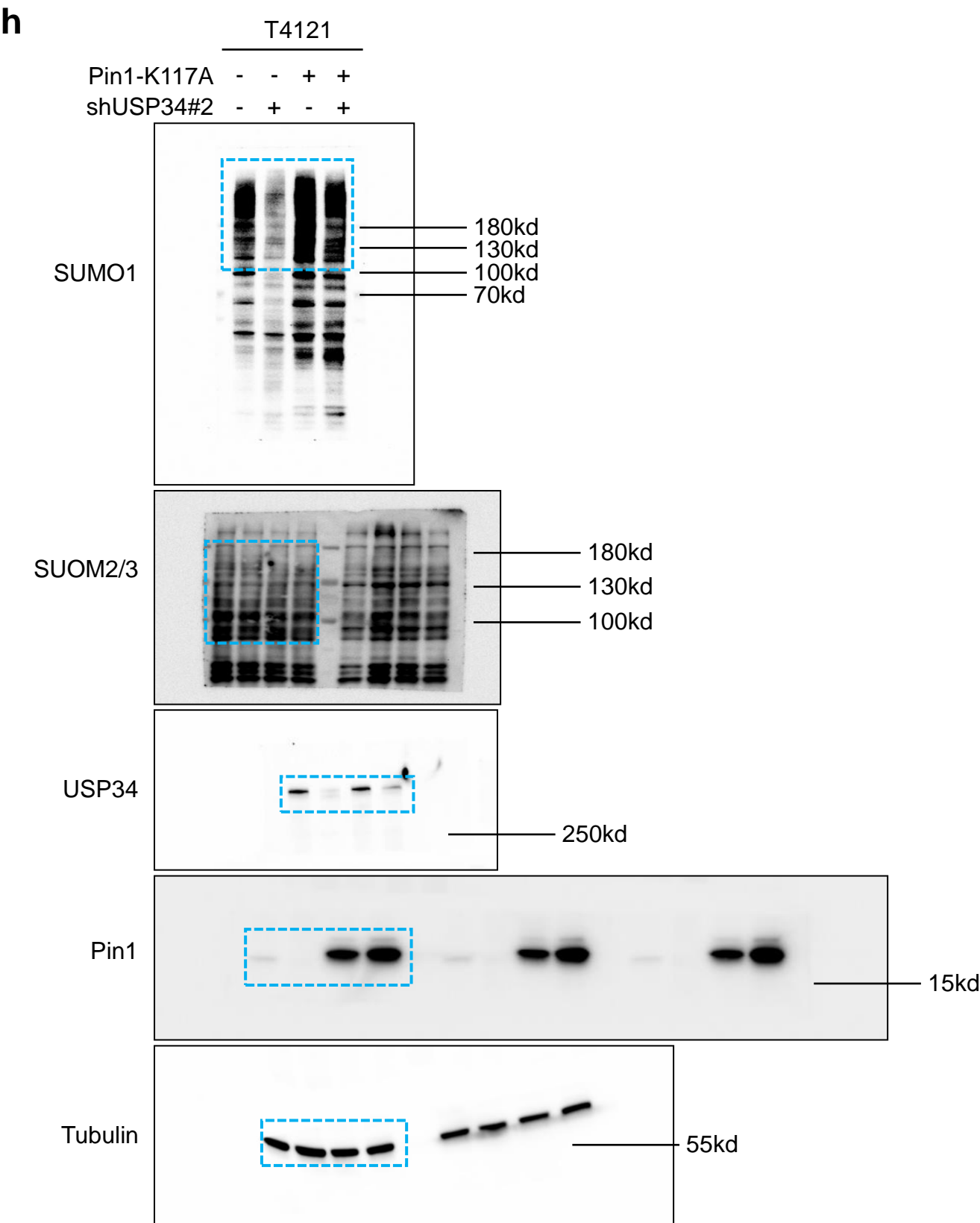

**a**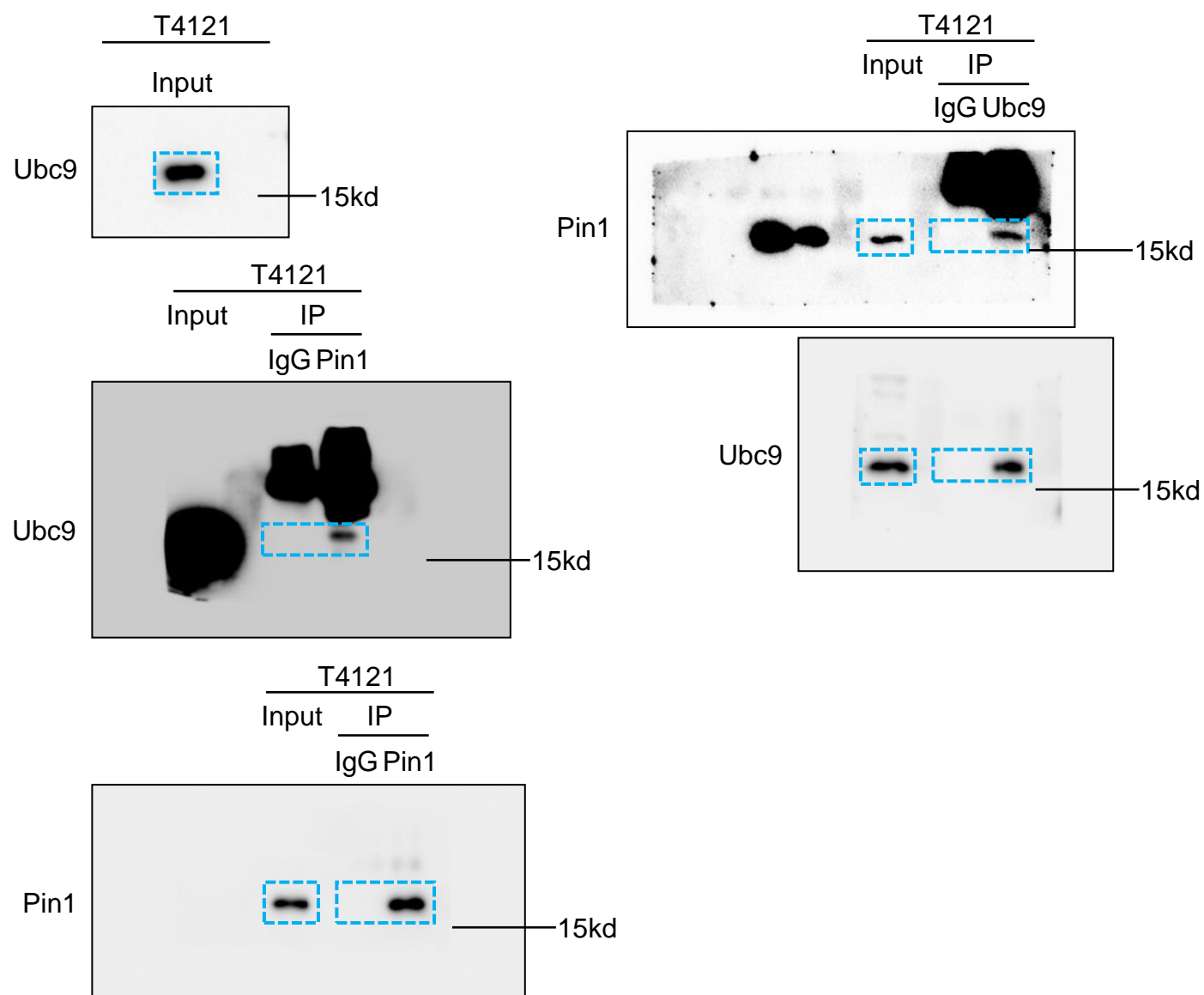**d**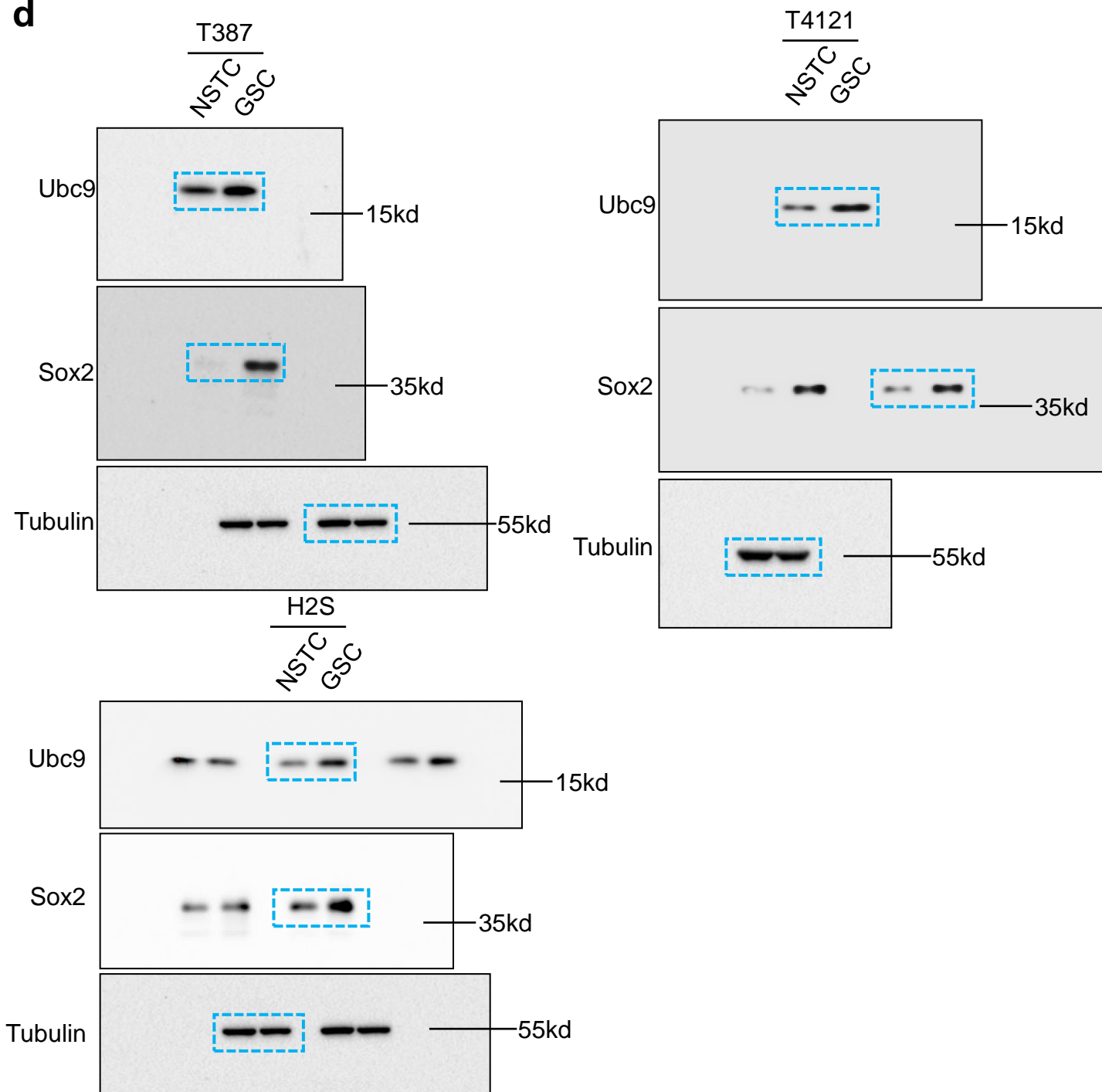

**e**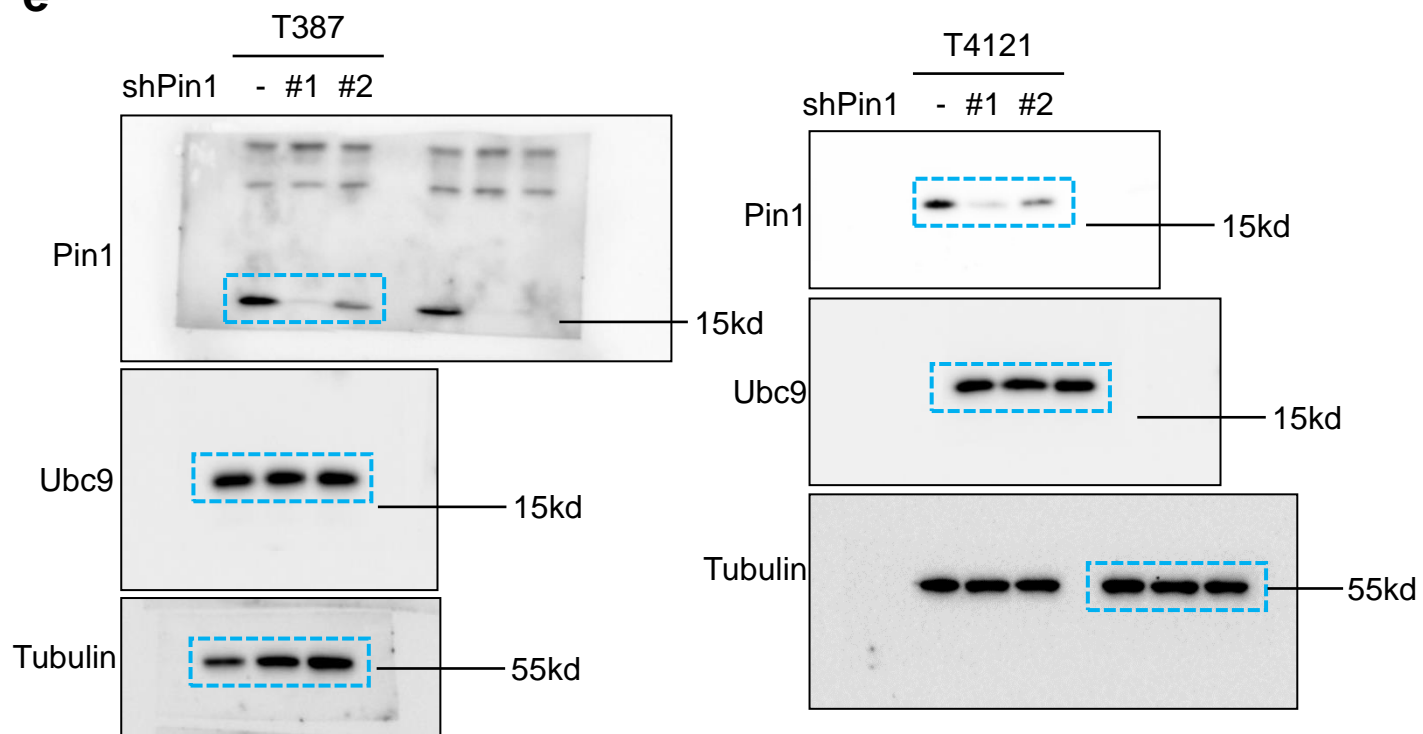**f**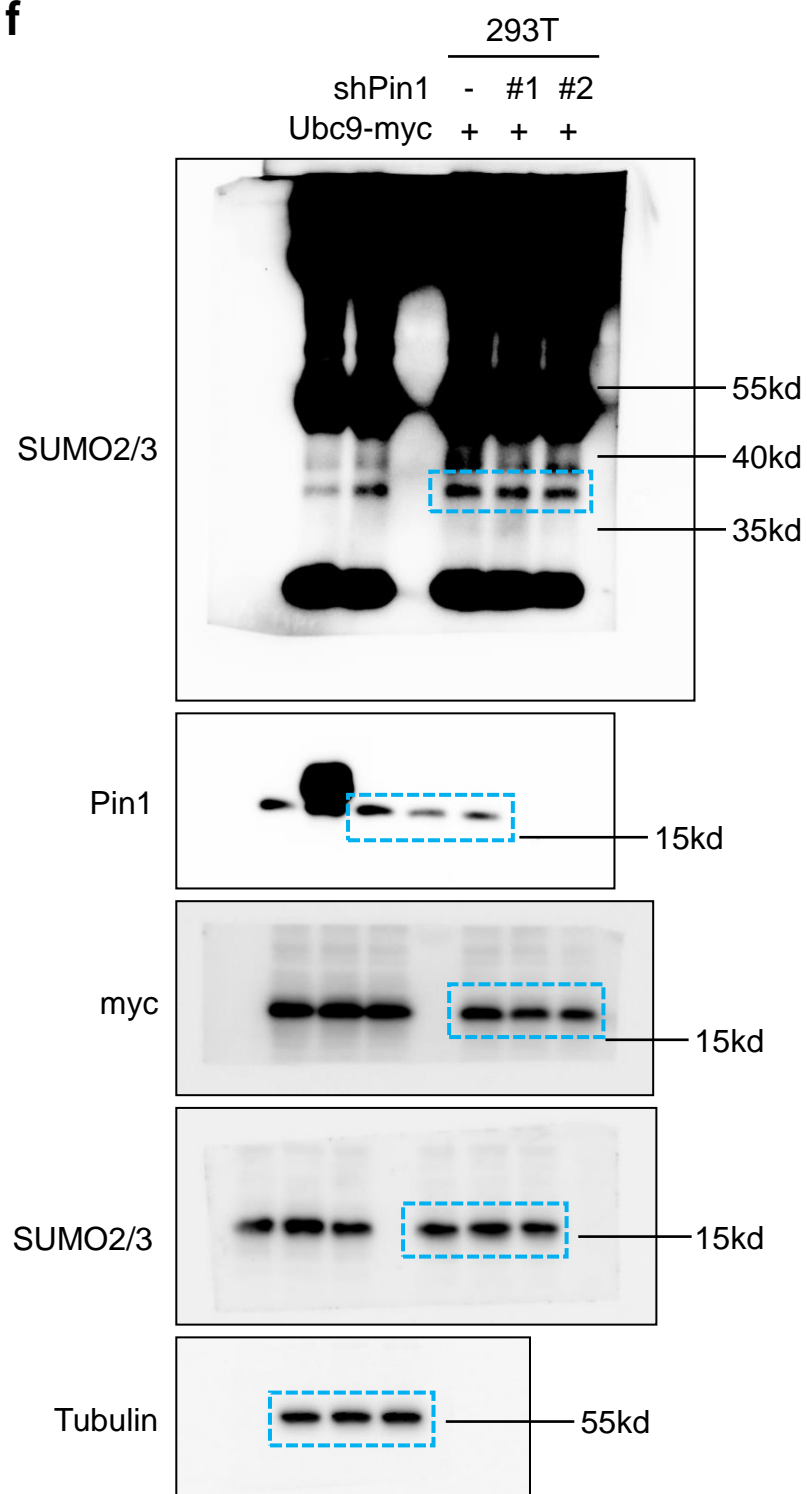

**g**

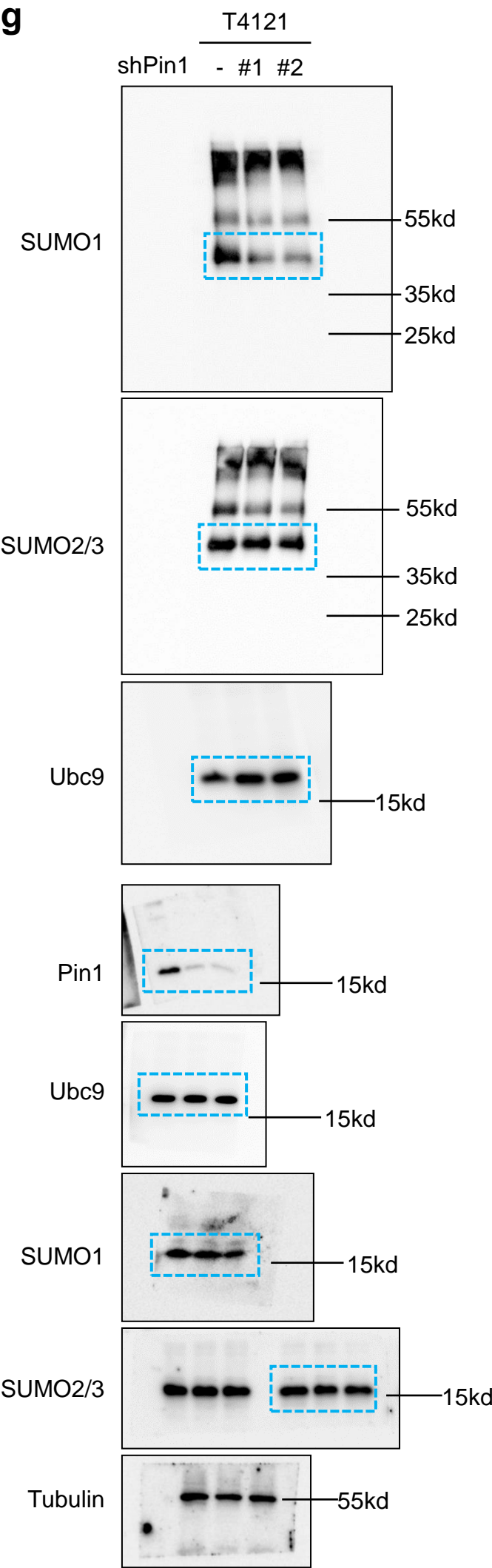

**h**

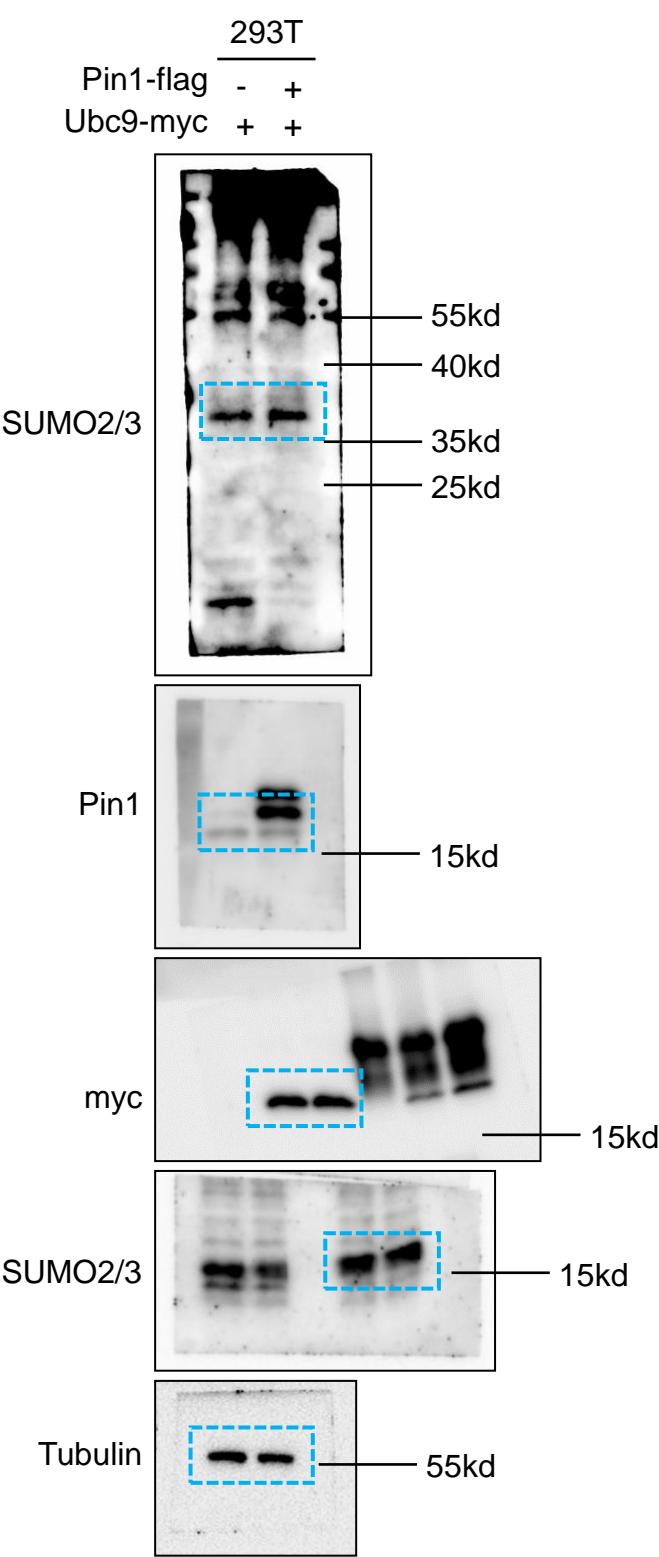

i

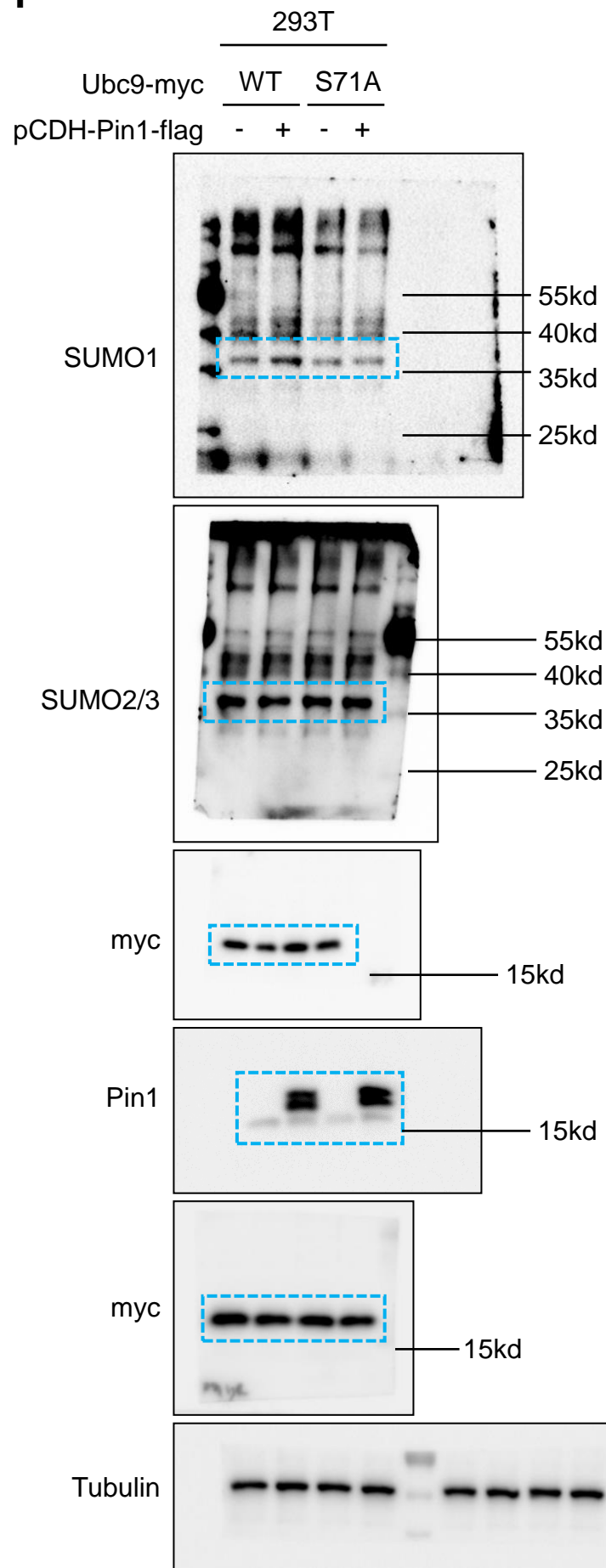

j

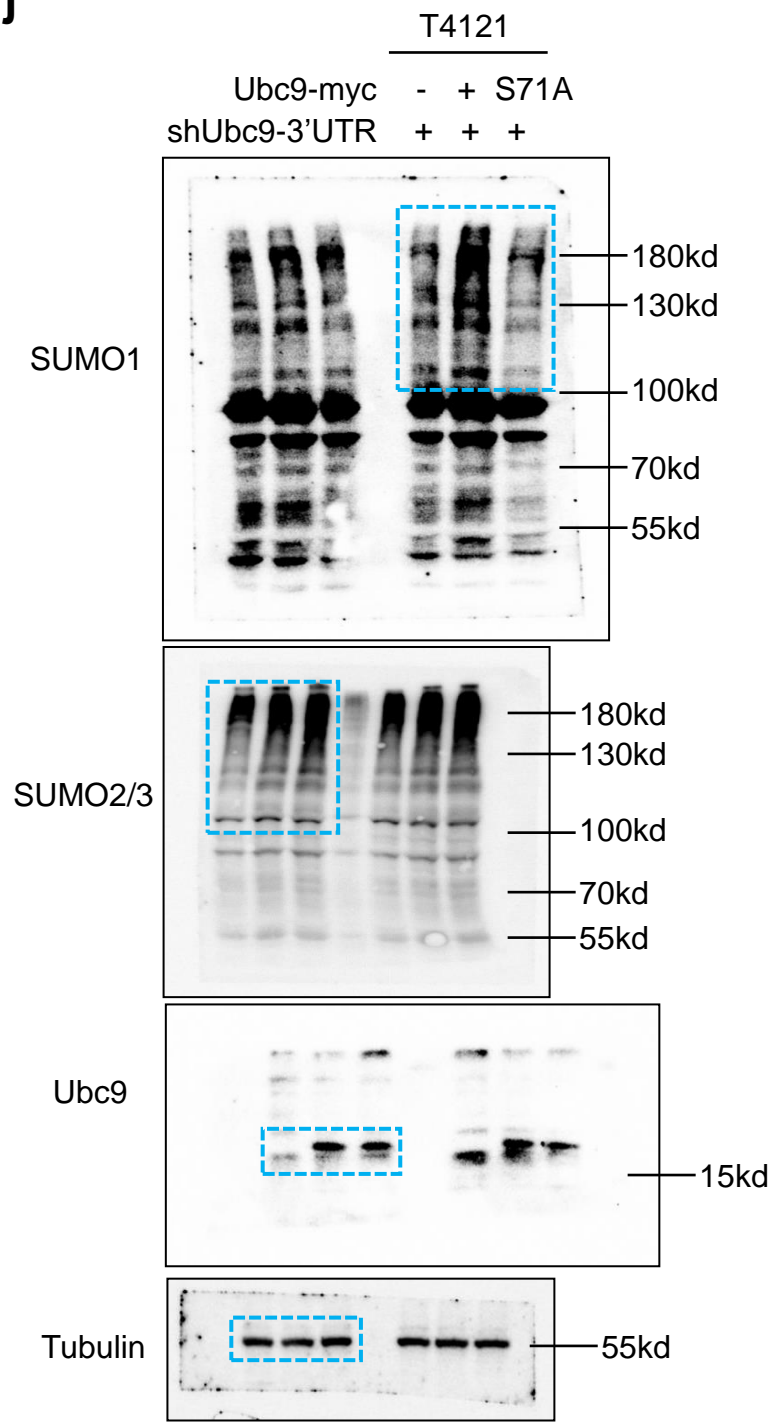

**b**

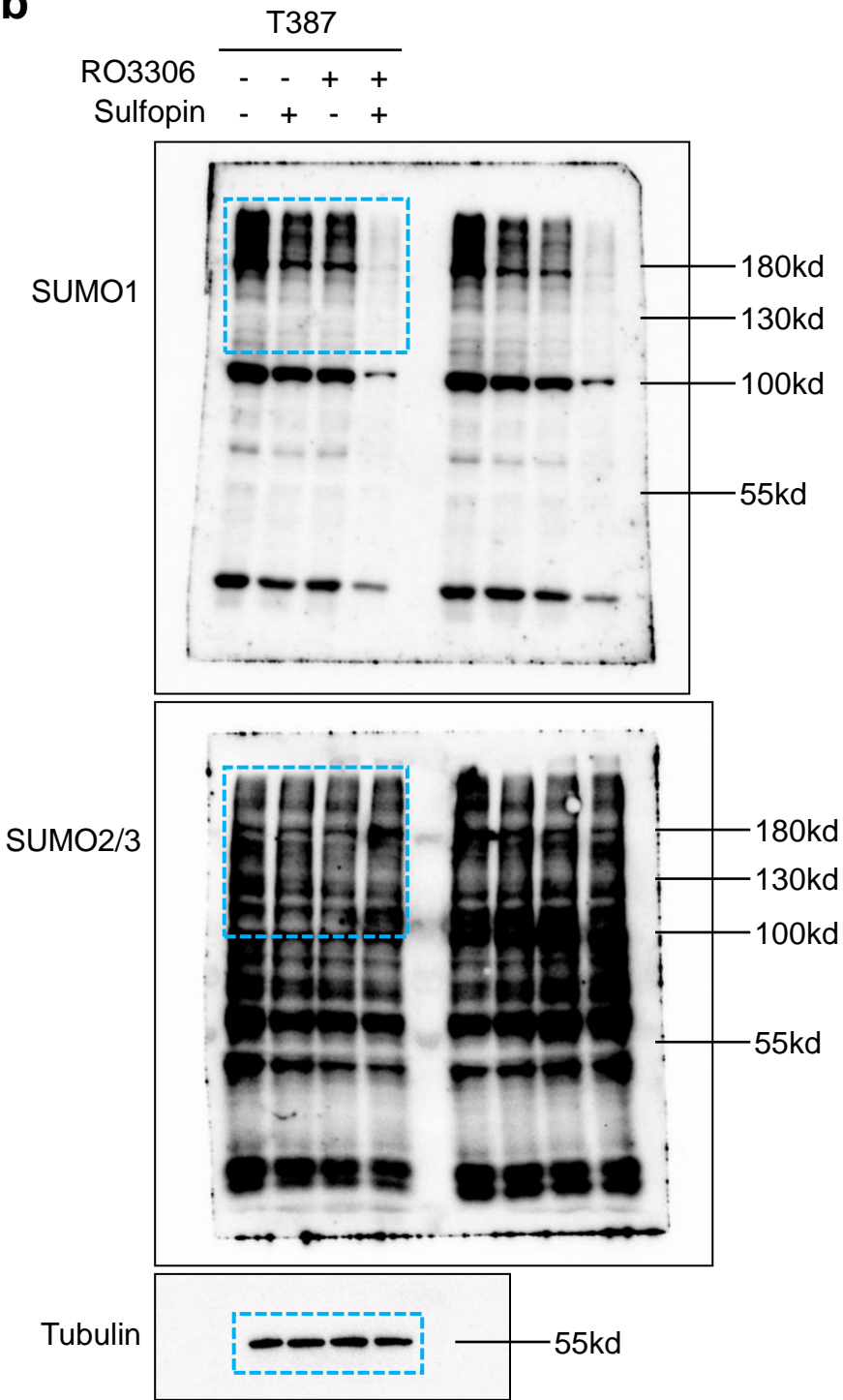

**a**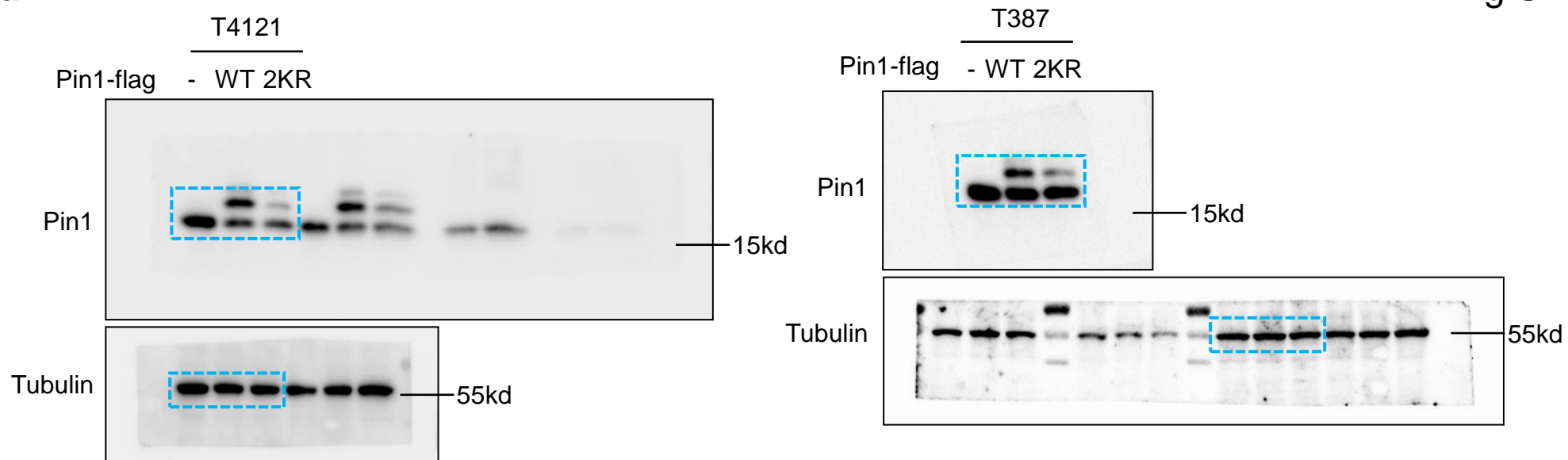**b**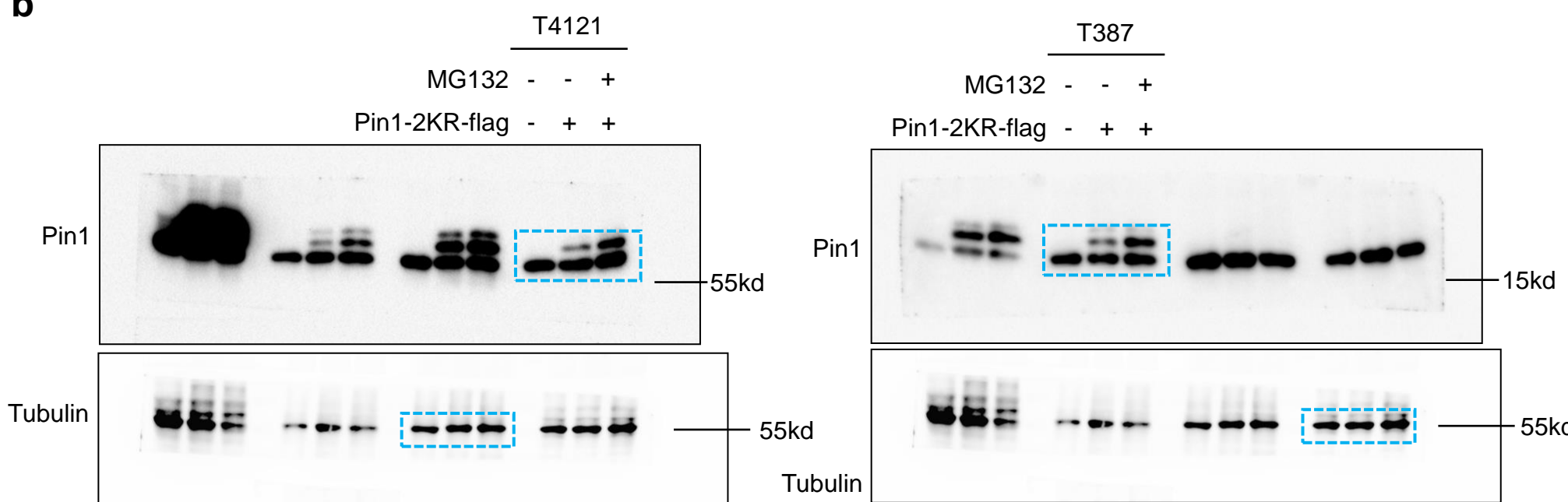**c**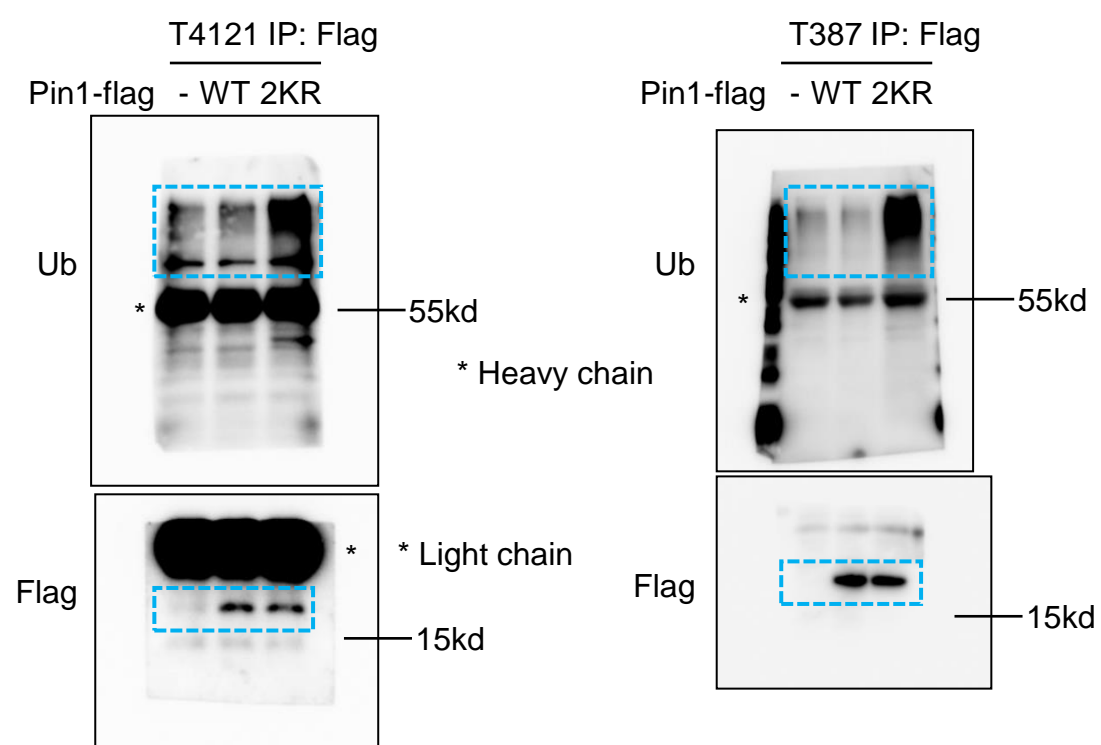

**d**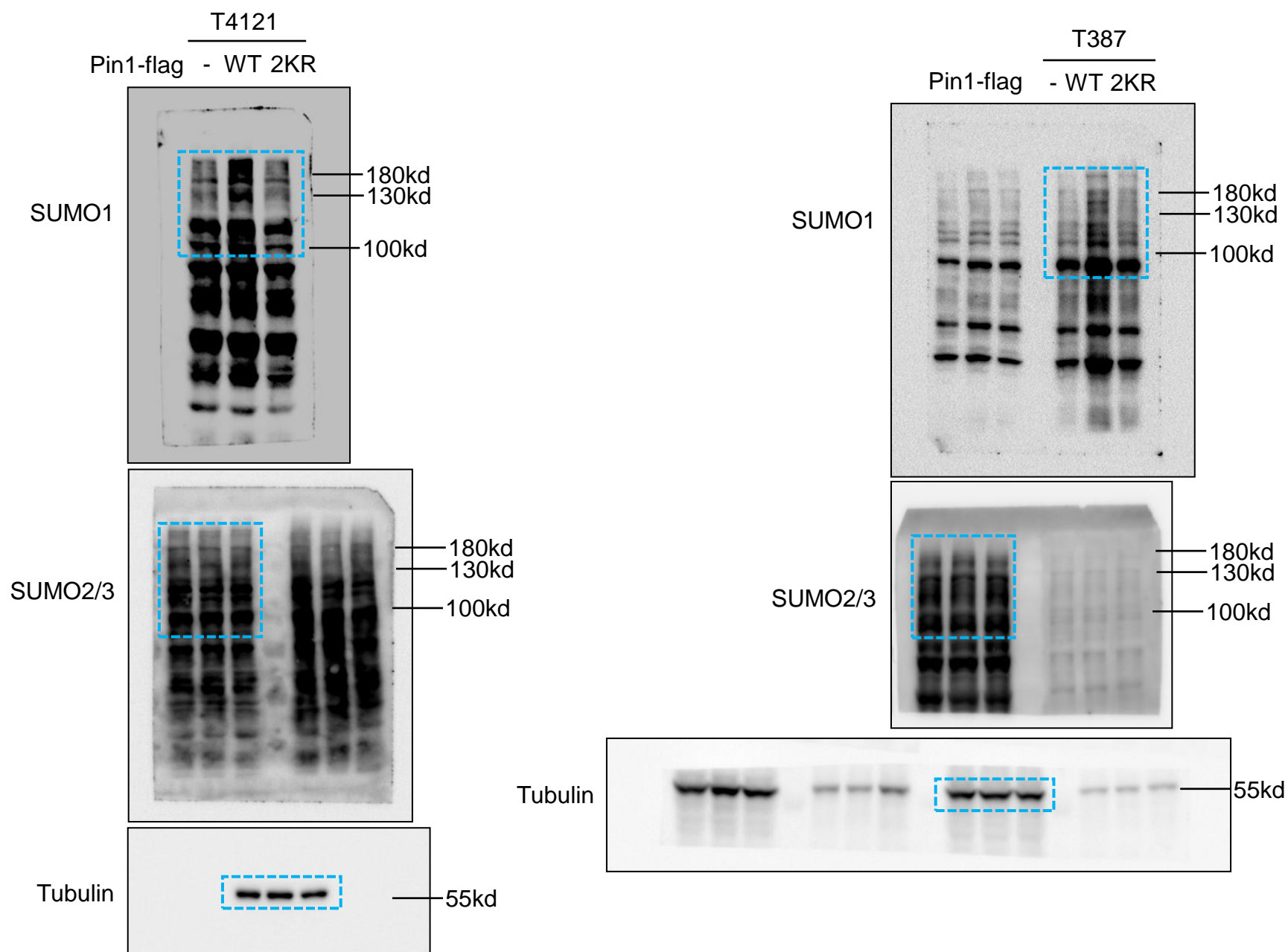**e**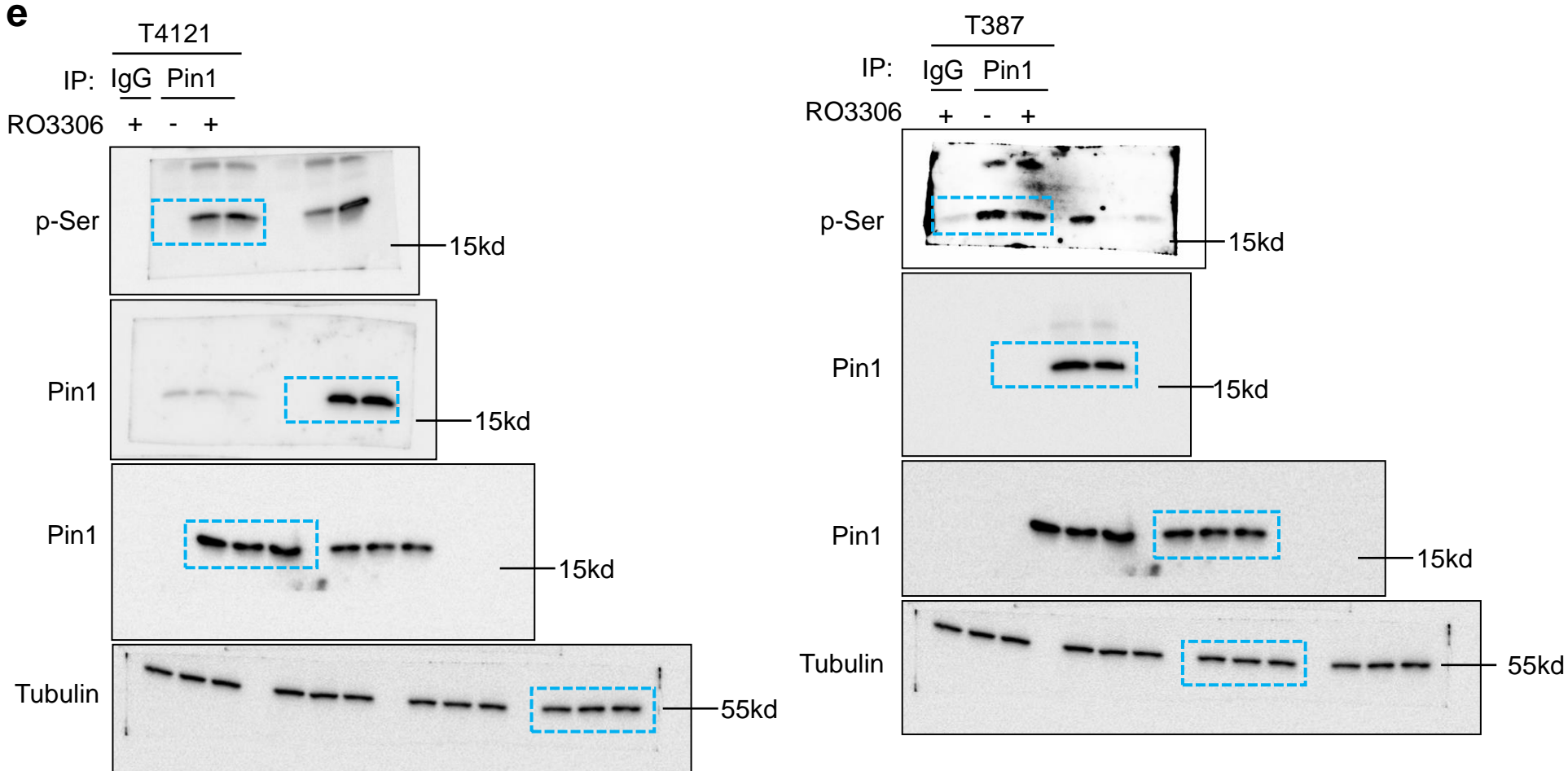

**f**

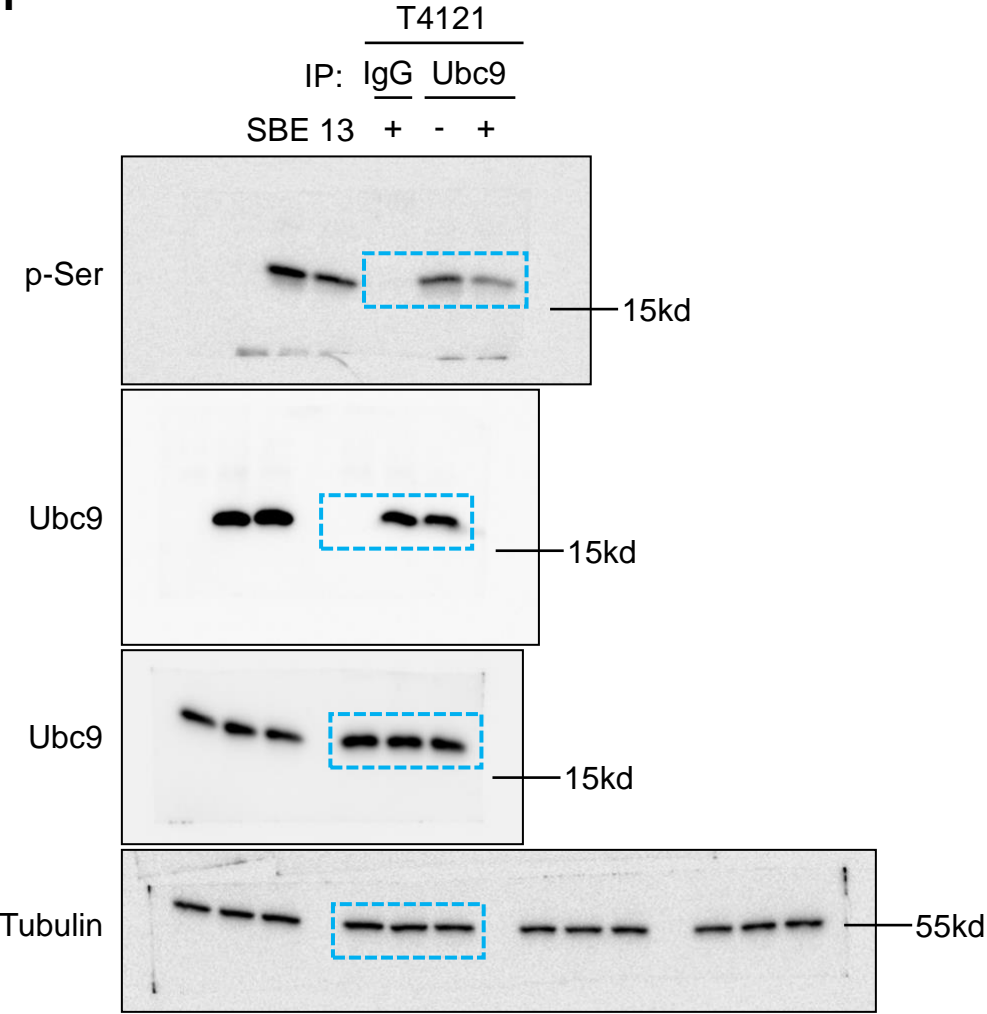

**g**

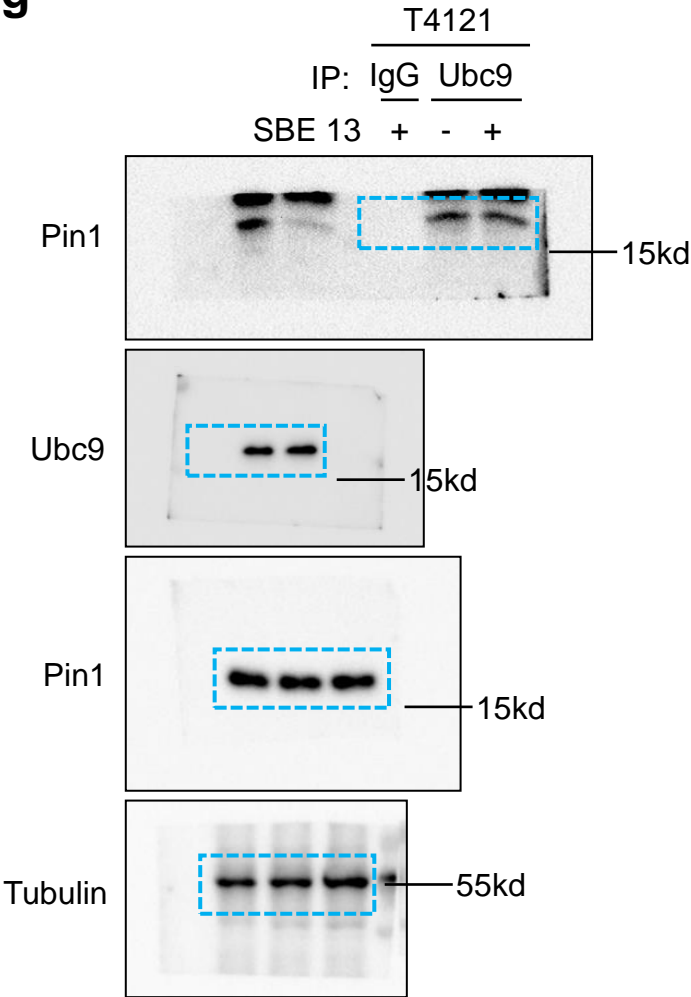

Supplement: Supplementary file 4 — Source Data [file 41467_2023_44349_MOESM4_ESM.zip › Raw Immunoblot data.pdf]
